# A critical review: developing a birth integrity framework for epidemiological studies through meta-ethnography

**DOI:** 10.1186/s12905-023-02670-z

**Published:** 2023-10-10

**Authors:** Stephanie Batram-Zantvoort, Lisa Wandschneider, Oliver Razum, Céline Miani

**Affiliations:** 1 Department of Epidemiology and International Public Health, School of Public Health, Bielefeld University, Bielefeld, Germany; 2 Sexual and Reproductive Health and Rights Research Unit, Institut National d’Études Démographiques (Ined), Aubervilliers, France

**Keywords:** Disrespect and abuse, Respectful maternity care, Maternal health, Childbirth experiences, Mistreatment, Obstetric violence, Birth integrity framework, Meta-ethnography

## Abstract

Over the past decade, there has been growing evidence that women worldwide experience sub-standard care during facility-based childbirth. With this critical review, we synthesize concepts and measurement approaches used to assess maternity care conditions and provision, birth experiences and perceptions in epidemiological, quantitative research studies (e.g., obstetric violence, maternal satisfaction, disrespect or mistreatment during childbirth, person-centered care), aiming to propose an umbrella concept and framework under which the existing and future research strands can be situated. On the 82 studies included, we conduct a meta-ethnography (ME) using reciprocal translation, in-line argumentation, and higher-level synthesis to propose the birth integrity multilevel framework. We perform ME steps for the conceptual level and the measurement level. At the conceptual level, we organize the studies according to the similarity of approaches into clusters and derive key concepts (definitions). Then, we ‘translate’ the clusters into one another by elaborating each approach’s specific angle and pointing out the affinities and differences between the clusters. Finally, we present an in-line argumentation that prepares ground for the synthesis. At the measurement level, we identify themes from items through content analysis, then organize themes into 14 categories and subthemes. Finally, we synthesize our result to the six-field, macro-to-micro level birth integrity framework that helps to analytically distinguish between the interwoven contributing factors that influence the birth situation as such and the integrity of those giving birth. The framework can guide survey development, interviews, or interventional studies.

**Supplementary Information:**

The online version contains supplementary material available at 10.1186/s12905-023-02670-z.

## Introduction

In the past decade, health research on maternal and newborn health has undergone a transformative process. The former focus on birth outcomes (e.g., mortality, morbidity, cesarean section rate) expanded to take into consideration how health systems conditions and care processes impact maternal and newborn outcomes, now resulting in a more comprehensive set of indicators [1]. The emergence of maternal health metrics has shifted attention to how the provision of maternal healthcare is experienced from the parturients’ view. This broadening of perspective developed into a research stream that deals with a women-centered evaluation of maternity care provision, ranging from studies on maternal satisfaction [2] to childbirth experiences [3] and person-centred care [4]. The starting point for investigating how to humanize the conditions of childbirth was set in 1985 by a group of interdisciplinary experts [5, 6]. Research then intensified as a consequence of the landscape analysis by Bowser and Hill in 2010, who were the first to provide evidence on disrespect and abuse in facility-based childbirth [7, 8]. The research on substandard or even violent care provision was further developed ever since, e.g., through the framework of obstetric violence [9]. In 2014, the initial evidence on women facing mistreatment during childbirth generated a response from the World Health Organization (WHO), calling for intensified research and action to prevent and eliminate disrespect and abuse toward women in childbirth [10]. Since then, WHO has supported the enhancement of maternity care provision and strengthening maternal rights, as exemplified with recommendations on intrapartum care for a positive childbirth experience in 2018 [11, 12]. In parallel, birthrights organizations have put effort into developing the Respectful Maternity Care Charter [13], which has been taken up in numerous studies and has broadened the research field [14, 15].

Yet, the beforementioned concepts and frameworks (and related approaches) differ in part significantly from each other when it comes to the operationalization and measurement of the concepts’ main themes (e.g., consented care, respectful care) [16] and to the definitional outreach of the concept itself [17]. Researchers interested in designing quantitative studies on the conditions and provision of maternity care and on birth experiences face the challenge of identifying a conceptual model that fits their aims and captures the dimensions of relevance from a multitude of existing (and varying) pool of measures and items.

Coming from an epidemiological, quantitative, and sociological perspective, we are interested in the conceptual approaches and themes that have been measured in relation to maternity care provision and birth experiences. More precisely, we aim to gather a comprehensive picture of the studied dimensions that constitute respectful, humanized, and dignified care and, vice versa, disrespectful, abusive, or violent care. Our overall research aim is to propose an umbrella concept and framework under which the existing and future research strands can be situated. Therefore, our research objectives are threefold:

- First, to identify and delimit the existing research lenses (frameworks, concepts, terminologies) that are concerned with maternity care provision and the experience thereof (Explanatory model).
- Second, to assess the themes that have been quantitatively measured in research on maternity care provision and birth experiences (Operationalization).
- Third, to synthesize the explanatory models and operationalizations into a new framework that conceptualizes the interwovenness between the provision of maternity care and articulates them as determinants of birth perceptions (Synthesis).

## Materials and methods

We conducted a critical review, which allows to step beyond the literature’s mere description and seeks to include a certain degree of conceptual innovation, often transitioning into a new model [18, 19]. For data analysis, we used a meta-ethnographic approach that serves to produce new insights upon a topic by comparing, pooling, and analyzing studies through qualitative meta-synthesis [20–22]. Meta-ethnography is suited for analytical (rather than descriptive) findings as the reviewer re-interprets each study’s ‘conceptual data’ and transcends them into ‘higher-order themes’. Meta-ethnographic studies are usually based on qualitative data. As our focus is exclusively directed on quantitative studies, we treated the definitions and measures (and the single items that compose them) as equivalent to qualitative data. We consider meta-ethnography to be the most appropriate methodology to meet our research aim, i.e., a conceptual contribution. Meta-ethnography consists of three phases and seven steps that are iterating and overlap circularly until analytical saturation is reached [23].The eMERGE reporting guidance for meta-ethnography [24] can be found in Additional file 2***.***

### Phase I

The first three steps are equivalent to other review methodologies, and can be described according to the PRISMA guidelines and flowchart for reporting systematic reviews of qualitative and quantitative evidence [25]: (1) Identifying and defining the topic and purpose of the review, (2) Deciding what is relevant to the initial interest (3) Reading the studies. The search streategy was developed according to the PICO format [26] using Boolean operator. For P (Population), we subdivided the search terms into birth (e.g. “birth”, “delivery”, “labour”) and setting (e.g. “maternity ward”, “obstetric care”, “birth clinic”) and connected them by OR. We connected I (Intervention) and C (Comparison) by OR as well, listing search terms for the violations of maternal rights or deficient care (e.g. “birth violence”, “discrimination”, “disrespect”) and the protection of maternal rights or respectful maternal care provision (e.g. respectful maternity care, self-determination, autonomy, informed consent). Electronic searches were conducted in PubMed, PsychInfo, CINAHL and Embase. Studies that we considered eligible for inclusion researched aspects directly relevant to the quantitative measurement of maternal care conditions or provision, birth experiences or the perception of birth. We excluded studies assessing maternity care’s general quality, access to and utilization of maternity health care services or pregnancy care, and studies on violence outside the context of labour and birth. Setting-wise, we included studies on facility-based childbirth (e.g. hospitals, obstetric clinics, birthing centres) and excluded studies on assisted and non-assisted home birth. Concerning the population, we included people during childbirth and early postpartum phase and attendees of the birthing context (e.g. health care professionals, doulas, partners). We excluded studies on people using reproductive health services other than those revolving around childbirth (e.g. fertility treatment, abortion). Primary quantitative studies of different types were included (e.g. cross-sectional, cohort-studies), whereas qualitative and mixed-methods studies and secondary research, reviews, editorials, commentaries or conceptual articles were excluded. The scope was limited to studies published between 2010–2020 since the thematic framework proposed by Bowser and Hill in 2010 [8] led to a significant increase in research.

After having included and read all eligible studies, we extracted all relevant information into a pre-structured data extraction table. The table comprised general study information (e.g., country, population, study aim), the definitional scope of the frameworks, concepts or terms, and the items used for measurement. For more information on search strategy, data extraction, and a comprehensive overview of the studies’ characteristics, see: Additional files 1, 3, and 4*.*

### Phase II

Determining how the studies are related (4), Translation of the studies into one another (5), and Synthesizing translations (‘in-line-argumentation’) (6). For this phase, we distinguished between two levels of analysis: the conceptual and measurement levels. The ‘data’ for the conceptual level refers to the lens through which the studies approach their research (e.g., terminologies, concepts, framework). We first grouped studies that showed similarities in their approaches into conceptual clusters. Then, essential terms, frameworks, or underlying perspectives were derived to create a shared definition for each conceptual cluster. Subsequently, we compared the metaphors and concepts of one article within clusters with those in others. We elaborated each clusters’ primary focus, distinguishing from each other the different approaches while at the same time indicating affinities between the research strands. Then, we performed an in-line argumentation that prepared ground for further conceptual development, including a differentiation between measurement components. At the measurement level, the ‘data’ is constituted of the individual items of each study’s data collection instrument. All items of each study were tagged with an underlying theme by using content analysis (e.g., “Some health workers shouted at me because I haven’t done what I was told to do” →  verbal abuse). Then, we compared each article’s items-themes with those in others to condense and merge (‘translate’) themes into one another. Themes were grouped by similarity, and we again used content analysis to create specific, but not redundant, overarching categories and subthemes.

### Phase III

Express the synthesis (7). Syntheses can be expressed by designing a framework, model, hypothesis, or theory that is supported by findings from the previous steps. Driving from the conceptual level analysis, we will introduce the concept of birth integrity. Based on this, and on the results of the measurement level, we present a six-field framework in which all categories identified at the measurement level were placed.

## Results

**Fig. 1:**
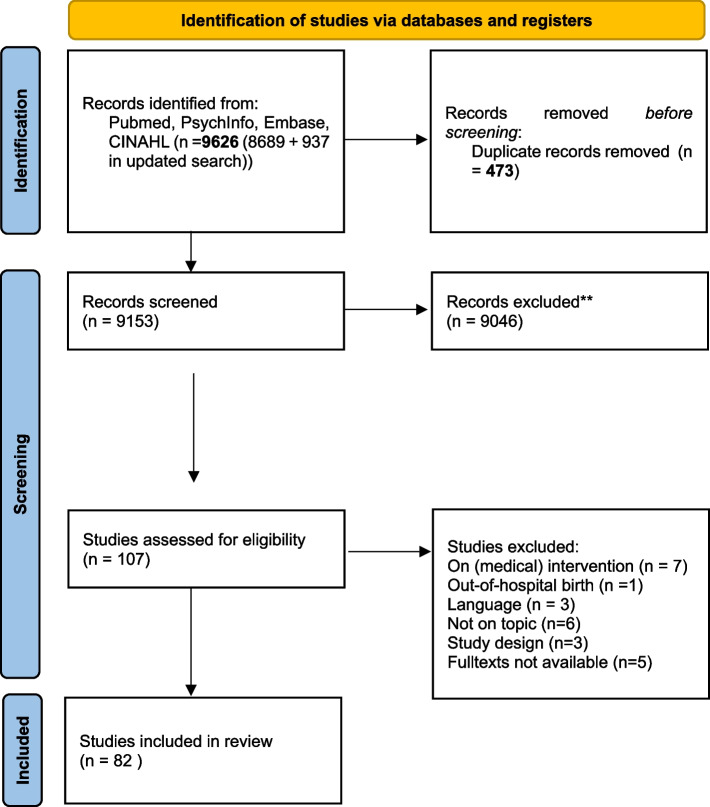
PRISMA 2020 flow diagram

Our searches yielded 8689 hits in April 2019 and 937 hits in March 2020 (update conducted in PubMed only). After removing duplicates, we screened 9153 records based on the title and abstract. This led to the exclusion of 9046 records (Fig. 1). We read in full text 107 publications, of which 82 studies met the predefined inclusion criteria. All relevant information was extracted in a data extraction table (Additional file 4).

### Conceptual approaches to measuring care provision and birth experiences in health research

To meet our first research objective, we identified and delimited from each other the existing frameworks and concepts through which research maternity care provision and birth experiences were researched. We arrived at 6 main conceptual clusters, namely: Disrespect and Abuse/ Mistreatment during facility-based childbirth (D&A/ MISC) [27–56], Respectful maternity care (RMC) [57–63], Obstetric violence (OV) [64–70], Person-centered care (PCC) [71–75], Childbirth experiences (CE) [76–82], Maternal satisfaction (MS) [83–98]. We grouped concepts that were marginally used in a separate cluster named “Other” [99–107]. The results are presented in Table 1. The key concepts that were built therefore reflect the ‘shared quintessence’ of each cluster. An overview of the process of extracting key concepts can be found in Additional file 5.

**Table 1:** Key concepts for each cluster of studies

Conceptual cluster	Key concept
**D&A, MISC**	Disrespect and abuse reflect any form of inhumane treatment or uncaring behavior towards a woman during labor and birth. D&A represents a fundamental violation of women’s human rights and undermines the safety and effectiveness of health systems, e.g., through non-dignified care, non-consented care, neglect or abandonment, or lack of privacy. Mistreatment (MISC) in childbirth describes childbirth-related mistreatment at an interpersonal but also at the health-system level and comprises seven domains: 1. physical abuse, 2. sexual abuse, 3. verbal abuse, 4. stigma and discrimination, 5. failure to meet professional standards, 6. poor rapport between women and providers 7. health system conditions and constraints. Drivers of D&A/MISC can include systemic failures, such as overwhelmed health care administration, poor staffing, and inadequate infrastructure
**RMC**	RMC is a universal human right due to every childbearing woman in every health system around the world in which maternity care goes beyond the prevention of morbidity or mortality to encompass respect for women’s basic human rights. Components of RMC are freedom from harm and ill-treatment; Right to information, informed consent and refusal, and respect for choices and preferences, including the right to companionship of choice whenever possible; Confidentiality, privacy; Dignity, respect; Equality, freedom from discrimination, equitable care; Right to timely health care and to the highest attainable level of health; and Liberty, autonomy, self-determination, and freedom from coercion
**OV**	OV addresses facets of dehumanized care and any action or omission by both health personnel and the health care system that physically or psychologically damaged or denigrated a woman. OV includes medical negligence, improper medication, pathologizing of/inconsideration for natural processes of childbirth, postpartum and female reproductive processes, and forced sterilization. OV links to the concepts of structural and gender violence. Structural violence includes the lack of access to health care services and any kind of health discrimination due to a woman’s education, poverty, ethnicity, or other social vulnerabilities
**PCC**	Person-centered care is respectful of and responsive to individual patient preferences and needs, ensuring that the patients’ values guide all clinical decisions. Elements of PCC are 1. treating the patient with respect, 2. providing care in a non-threatening manner, 3. working in collaboration as equal partners, and 4. giving priority to the patient’s preferences over that of the healthcare provider
**CE**	Childbirth experiences and especially a woman’s relationship with her health care providers in maternity settings significantly impact her health. It has long-term implications for her future emotional, physical, and reproductive health and wellbeing. Negative CE increases the risk for postpartum depression, secondary fear of childbirth, and post-traumatic stress disorder
**MS**	Maternal satisfaction refers to a woman’s subjective and dynamic evaluation of her birth experience. This multifaceted construct includes elements of perceived quality of care, coping efficacy, and reflections of the birth experience as a whole and in context. Low MS can affect the mother’s and infant’s health. Low levels of MS are associated with greater odds of postnatal depression, post-traumatic stress disorder, requests for future elective cesarean section, sterilization, and abortion
**Others**	
Medical ethics **(ME)**	Medical ethics comprises the four ethical principles patient autonomy, nonmaleficence, justice, and beneficence
Patient’s verbal participation** (PVP)**	A patient’s verbal participation influences the quality of care, which is, in turn, related to health outcomes. Predisposing factors influence how a person communicates with a health provider. Enabling factors affect communication participation levels. Communication by the health care provider is the final factor that influences the ways and extent to which patients participate
Informed consent **(IC)**	Informed consent plays a vital role in clinical decision-making. It is a basis of self-determination in health care. In ideal situations, health care professionals inform their patients about all relevant aspects of care and alternative care options, map the value system of the patients, and adjust the information process accordingly. Patients and health care professionals have shared responsibility in the process
Self-efficacy, control **(SEC)**	Self-efficacy during birth is associated with less anxiety and a greater perception of control during birth. Support from healthcare professionals is more important than the event of birth
Responsive-ness **(RESP)**	Responsiveness addresses non-clinical aspects of health service quality relevant regardless of provider, country, health system, or health condition. It comprises factors related to health system interactions and health system environments, e.g., respect for human dignity and client orientation
Support and Control **(SC)**	Caregivers must be supportive and create an atmosphere that allows a woman to gain autonomy over birth. Supportive care helps women obtain their control and enhances dignity during childbirth
Maternal welfare **(MW)**	Maternal welfare includes six domains: Quality of relationship during care, self-care, and comfort, conditions that allow contact between mother and child, personalized care, continuous participation of the family, and timely and respectful care
Mothers on Respect **(MOR)**	Mothers on respect captures the mother’s sense of disrespect and dismissal, especially when engaging in conversations with providers. This concept is closely related to autonomy and informed consent

The cluster D&A/MISC shows a strong focus on care interactions between healthcare professionals and the parturient and specifically emphasizes experiences of poor maternal care. In contrast to D&A/MISC, RMC takes a right-based and ethical perspective in claiming universal and fundamental rights for every woman in childbirth. While many of the rights correspond to D&A/MISC in building the desirable opposite, it is important to note that RMC highlights specific rights in childbirth that reach beyond the mere absence of abusive or disrespectful acts. OV shows high similarity to D&A/MISC and additionally points to the wider societal dimensions of structural and gender-based violence and addresses structural, cultural, and healthcare-related drivers that favor violative conditions, processes, and behaviors in obstetric care. PCC shows affinities with RMC and highlight that maternity care should be respectful of and responsive to the parturients’ preferences and needs, ensuring that the parturients’ values guide all clinical decisions. (CE) takes the individual’s evaluation of their birth as their starting point, claiming that the subjective experience of childbirth and the quality of relationship with the healthcare professional impact postpartum health and wellbeing. A similar focus is visible in the studies in the MS cluster: here, the impetus lies within the parturient’s expectations towards and feelings during birth, the satisfaction with the care provided, and how this impacts post-partum health.

### Categories identified through meta-ethnography

With our second objective, we aimed to depict how the quality of maternity care provision and birth experiences have been operationalized and measured in quantitative studies. In all, we identified 72 themes and subsequently organized these by defining 14 overarching categories. We briefly introduce the categories and their related subthemes. An extensive overview of all underlying analytical steps (items, content analysis, juxtaposition of themes) and a presentation on the final themes organized by research strand (e.g., D&A, OV, RMC) is available in Additional file 6.

1. *Health service capacity*: The health service capacity concerns the availability of a healthcare facility nearby the parturient’s residence. Closely linked is the accessibility of the facility in relation to the environment and the infrastructure (e.g., public transport). Last, this category includes the availability of specialized obstetric services and trained maternal and newborn care staff in the health facility.
2. *Societal discriminatory norms*: Societal discourses on gender norms and racism generate inequalities of treatment between women with different social identities and positions. This category encompasses attitudes towards gender norms or the perception about whether racism comes into play in labor room interactions.
3. *Facility*: includes a health facilities’ basic equipment (beds, rooms, running water and electricity) and supplies (basic drugs and medication, postpartum supply, food), hygiene conditions, and equipment to protect privacy and enable comfort (e.g., partition walls, cleanliness of bathrooms, comfortability, and condition of rooms).
4. *Professional care*: includes the quality of care provision, including the competency of the healthcare provider (e.g., well-trained midwives and obstetricians), adherence to medical guidelines and evidence-based care standards, the implementation of hygienic practices to ensure the provision of safe medical care healthcare.
5. *Organization of care and management of the facility*: a health facilities’ care capacity (staffing capacity, timely care, continuity and choice of the care provider, room planning), the information management, and the duration of hospitalization (appropriate lengths of stay) relates to a facility’s ability to organize and manage the maternity care provision. Detention in the facility against the mothers’ will (e.g., due to unpaid bills) or a delay of discharge are manifestations of harmful care conditions.
6. *Dignified care*: Dignified care manifests in the way healthcare professionals approach the parturient, more specifically through respectful communication (appropriate language, calling the woman by preferred name, or talking calmly, approaching her kindly and in a culturally sensitive manner) and respectful treatment (appropriate and careful examinations). Non-dignified care includes verbal (shouting, insulting), emotional (threatening to withhold treatment, negative comments, blaming), physical (e.g., pushing, beating, slapping, pinching), or sexual abuse (e.g., inappropriate sexual conduct, harassment, rape).
7. *Information, explanation, consent*: refers to the comprehensibility and accessibility of information (e.g., provision of multilingual information sheets and, if needed, translators to obtain informed consent). It additionally includes an informative- and consent-seeking care culture that follows medical ethical principles of obtaining informed consent before conducting examinations, administering medication, or deciding on medical procedures (e.g., information on individual proceeding and diagnosis, information on choices, consistency of information, encouragement to ask questions, effective communication). Non-consented care and coercion to procedures reflect a restriction of self-determination and autonomy.
8. *Supportive care*: reflected through a person-centered care approach that prioritizes wellbeing and comfort throughout labor and birth. Supportive practices include pain management, assistance, and (physical) support (e.g., offer breathing techniques, encouragement to mobility, food- and drink-intake, asking for a companion of choice). A supportive attitude is shown through emotional support, engagement, empathy, and encouragement and by actively involving and empowering the parturient through shared decision-making.
9. *Birth accompaniment*: refers to an accompanying care culture, including the sufficient presence of health professionals during labor, (e.g., through frequent presence and attention, regular visits, and, if possible, continuous and 1-to-1 care). The opposite of presence at birth is a negligent care culture, including abandonment (e.g., leaving women alone during labor and birth). Birth accompaniment also includes cooperative and effective collaboration between care providers (e.g., respectful communication, respecting professional opinions and decisions) and the responsiveness and adequacy of reactions of healthcare providers towards the parturient, including the quick and proper feedback to requests (e.g., requested assistance, support, or administration of pain medication). Non-responsive care is reflected by denial or ignorance of the parturients’ requests.
10. * Confidential care*: reflects a culture of confidentiality, which includes confidential handling of sensitive data (e.g., health information), visual (curtains, physical barriers, closed doors), auditory (no bystander in provider-patient conversations) and situational privacy (e.g., no people coming in and out of the delivery room unnecessarily).
11. * Personal rights, ethics, and equity*: relates to fundamental maternal rights, including wishes and preferences for birth, companionship of choice, self-determination, autonomy (e.g., freedom of movement and birth position), and parental rights (e.g., decisions upon newborn’s health and nutrition). Discriminating or privileging the parturient based upon attributes or identities (e.g., race, sexual or gender, appearance), disagreement (e.g., different views upon healthcare), socio-economic background (financial status), or health insurance (e.g., denial of needed care) reflects non-equitable care, whereas requests for bribes or informal payment conflict with ethical care.
12. * Attitudes and expectations towards birth*: includes the attitudes towards childbirth (e.g., prior knowledge and birth experiences, wishes on low-intervention birth, fear of childbirth), childbirth efficacy (e.g., expectation towards own ability to manage labor pain, expectations about own capability at birth), and expectations of control.
13. * Childbirth perception and feelings*: the actual perception of one’s birth is measured through different aspects such the conformity of childbirth expectations and experiences, the feelings, emotions, and thoughts experienced during birth, both internal and self-reference (internal control, labour agentry, stress, anxiety, fear, satisfaction with self) and external reference (external control, feelings of security, safety, trust, being seen, feeling dignified and respectful). The perception of having experienced violence or abuse during childbirth makes another subtheme.
14. * Health consequences of violated birth experiences*: encompasses potential impairments of mental and physical health or wellbeing following a violent birth. This includes (posttraumatic) stress symptoms, birth trauma, postpartum depression, anxiety disorder or bonding and attachment, postpartum depression or disturbed mother-child-bonding, attachment, or lactation.

### Synthesis: The multilevel birth integrity framework

For the meta-ethnographic synthesis, we first derive from the conceptual analysis four principles that will guide the conceptual synthesis:

- Propose a concept that includes the care conditions (like D&A/MISC, RMC, OV, and PCC) and the individual’s experiences and perception thereof (like MS and CE);
- Include within this concept the desirable and functionable expressions of ‘good’ care conditions and the poor and non-functionable expressions of care;
- Reflect and contextualize birthing within its structural and gender dimensions;
- Justify the concept through a fundamental rights and ethical perspective.

We additionally see three components that need to be reflected in the operationalization of studies on maternity care provision and maternal experiences of birth:

- Determinants of birth experiences, e.g., drivers that shape the care condition, care culture or the provision of care, and the parturient’s subjective expectations towards giving birth.
- As childbirth is experienced differently by everyone, we propose a subjective outcome measure that reflects how the birth experience is individually perceived.
- Potential health and wellbeing consequences of one’s birth perception.

**Fig. 2:**
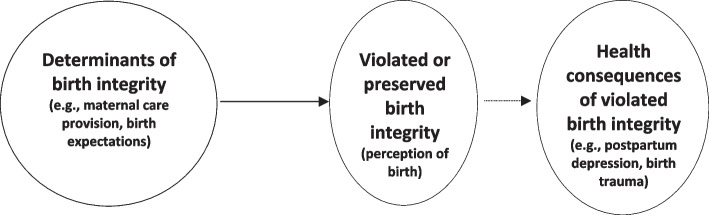
Conceptual model of birth integrity

We suggest birth integrity as an umbrella concept that includes the four principles and helps to distinguish between the three components outlined before (Fig. 2:

- Determinants of birth integrity,
- Birth integrity itself, and
- (potential) consequences of violated birth integrity.

Building upon the conceptual model of birth integrity, we make use of a multilevel framework that has initially been developed by sociologist Ritzer [108] for the social analysis of societies and distinguishes between a macro-and microlevel and a manifest and latent side. Manifest refers to anything objectifiable (e.g., directly observable, or assessable like language, laws, and interaction), whereas latent comprises of anything underlying or not directly assessable like culture, discourses, perceptions, or beliefs. We have adapted this framework by adding a third level of analysis (mesolevel) and applied it to our topic. The macro-manifest side e.g., comprises of structural factors such as the health systems (policies, coverage, legislation), whereas the macro-latent side includes a societies’ dominant discourse. On meso-level, the manifest side describes aspects related to institutions or organizations structures and processes, and the meso-latent level covers organizational cultures (shared norms, patterns of action). The individuals and their interactions with the environment reflect the micro-manifest level. Last, on the micro-latent level, the individuals’ expectations, and patterns of thought and action are organized.

Elsewhere, we theoretically underpin the multilevel birth integrity framework by outlining the concept within medicalization and risk theory, intersectionality, and embodiment theory [109]. By doing so, we open opportunities for further development in two directions: on the one hand, the multilevel birth integrity framework reflects the status quo of birth integrity-related measurements. Synchronizing the identified categories (and subtopics) with theoretical considerations demonstrate the gaps of current measurements. On the other hand, the evidence given by the categories identified in the studies’ measures (e.g., on non-consented care or non-evidence-based practices) can be taken up from theoretical (and health policy) positions and incorporated into existing discourses and policies.

In each of the six fields, we can place at least one category that reflects determinant of birth integrity.

On the macro-level, we assign in the manifest expression the category ‘Health service capacity’ and in the latent expression the category ‘Societal discriminatory norms’. We have matched most categories on the meso-level. On the meso-manifest side, we locate the categories: ‘Facility’, ‘Organization of care and management of the facility’, ‘Professional care’ and certain aspects of ‘Information, explanation, consent’. On the latent side, we assign the categories, ‘Dignified care’, ‘Birth accompaniment’, ‘Confidential care’, ‘Supportive care’ and ‘Information, explanation, consent’. On the macro-manifest side, we place the categories ‘Personal rights, ethics, and equity’ and on the macro-latent side category ‘Attitudes and expectations towards birth’.

Besides these categories identified as determinants, we characterized one category each for the subjective outcome of birth integrity (Childbirth perception and feelings) and the objective outcome (Health-consequence of violative birth experiences). Table 2 gives an overview of the multilevel birth integrity framework including the categories and subthemes of the macro-to-micro level in manifest and latent expression.

**Table 2:**
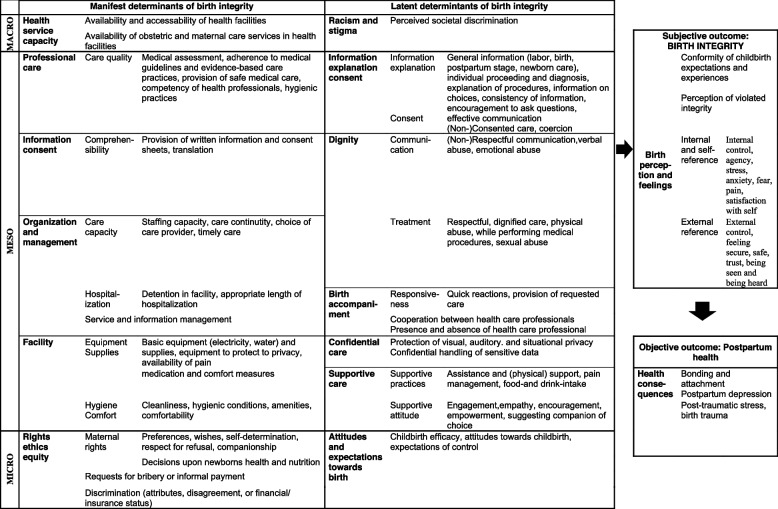
The multilevel birth integrity framework

## Discussion

In this critical literature review applying a ME approach, we first aimed to identify and delimit from each other recent research lenses on maternity care conditions and provision, birthing experiences, and maternal perception of birth. We met this aim by creating study clusters, providing shared definitions for each cluster, and elaborating each clusters’ specific focus upon the phenomenon. Based upon the reciprocal translation of the clustered studies, we deduced an in-line-argumentation resulting in key considerations that prepared ground for the pending conceptual synthesis.

Our second aim was to assess the operationalization and quantitative measurement of maternity care conditions, care provision, and maternal birth experiences. From all studies, we extracted items, labeled each item with a theme, and merged these themes into higher-order categories and subthemes. In all, we identified 14 categories that arise from and are related in different contexts: While twelve categories correspond to being a driver of maternity care provision or a determinant of how maternity care is experienced, one category refers to the perception of birth as violated or preserved birth integrity and another category to the potential health consequences of birth integrity violations. The concept and framework of birth integrity suitably distinguish from one another the determinants of birth integrity, birth integrity itself and potential adverse health consequences of violated integrity during birth.

Recurring to the variety of themes, we considered it useful to introduce a macro-to microlevel framework to differentiate between a manifest and a latent expression. Into the six fields, we placed all categories identified as determinants of birth integrity. Hence, the underlying (social) context of each dimension is obvious at first sight, e.g., staffing capacity of the facility at the meso-manifest level, a maternity care institution’s care culture at the meso-latent level, or one’s predispositions at the micro-latent level. In this way, the causes and expressions of poor and abusive conditions and maternity care provision can be more accurately identified and tackled through targeted interventions.

### Expanding the field: Determinants of birth integrity

Current epidemiological research reflects and integrates a multitude of birth integrity determinants into quantitative study designs. Most measures refer to the meso- and microlevel as they put the institution and the individuals involved in childbirth in focus (health facility, healthcare professionals, person who gives birth). We propose to consider to a greater extent the macro-level driver(s) that potentially determine how maternal care is executed. This implies a stronger focus on structural forms of disrespect towards women that have manifested in a lack of resources (human, equipment, infrastructure) in maternity care settings [110]. Thereby we have in mind the financing of obstetrics (e.g., remuneration system, insurance coverage), policies and laws concerning obstetric and midwifery care (e.g., on staff sub-limits, ensuring sufficient staff and bed availability to prevent overcrowding, laws addressing obstetrical violence), the availability of maternity clinics close to residence (in remote areas), free choice of birth location (e.g., midwifery-led clinics or labor rooms for low-risk-pregnancies, specified resourced facilities for high-risk pregnancies), the existence of routes to report experiences or observations of violations against fundamental rights in childbirth, and abusive events during obstetric or midwifery care [111]. We also consider it relevant to integrate macro-level indices of discrimination as potential determinants of birth integrity, e.g., gender equality indices or racial equity indices [112].

A society’s gender relations, practices, and norms have cultivated dominant narratives that are mirrored in expectations, ideas, semantics, and actions around childbirth (e.g., on femininity and motherhood). Research on birth integrity should include measures on a society’s dominant gender roles, on narratives of childbirth medicalization, or reflect on how childbirth is negotiated as ‘risky’ at the macro-latent level. By considering medicalization and risk discourses, light is shed on different knowledge and normative patterns that highly determine how practices around birth are organized (e.g. ‘biomedical’/ ‘technocratic’ vs. ‘women-centered’/ ‘humanized' paradigm of birth [113]).

Additional to the manifold determinants identified at the meso-manifest level, we propose to examine in more detail how a facilities’ amenities impact options for coping with labor progress or pains, e.g., availability of warm-watered tubs or space for motion to find relaxing poses or favor the progress of birth [114, 115]. Technical equipment (e.g., wireless electronic fetal monitor) can enable movement. Similarly, posters that illustrate body postures beneficial to labor progress may positively affect the course of birth. One of the most discussed and obvious support while birthing is the continuous, 1-to-1 support, usually provided by a midwife or doula [116, 117]. (Epidemiological) research on birth integrity should in more detail evaluate how continuous support impacts birth integrity.

At the meso-latent level, we identified a strong emphasis on how the person in labor and birth is approached in terms of communicative skills (e.g., effective communication, respectful tone). As language matters, a rather passive/ passivating or active/activating wording implicitly creates certain expectations and perceptions about one´s birthing capabilities and a reflection of (dis)respecting a childbearing individual’s autonomy. For example, while the phrase ‘a baby is delivered by the doctor’ terminologically puts the parturient into a waiting and passive position, the term ‘giving birth’ connotes the actively involved childbearing person [118].

At the micro-manifest level, more emphasis could be directed to birth-related discrimination. We think it is useful to integrate intersectional theory [119] into measurement endeavors, as it highlights how intersecting lines of difference along with race, gender, sexuality, ability, religion or class create privileged and marginalized positions that result in advantages and disadvantages. Taking an intersectional lens on care provision (e.g., upon frequency and duration of care attendance during birth) or more birth-related outcomes (e.g. scrutinizing racial disparities in maternal and newborn mortality [120]) might be fruitful for research on the determinants of birth integrity.

### Violated and preserved birth integrity

For (violated and preserved) birth integrity, we found in the studies validated scales that capture different aspects of birth perception, e.g., on agentry and control [121] or satisfaction [91, 122]. Yet, we consider it highly relevant, and will address in our future research, to develop and validate a specific scale that measures whether a person’s birth integrity felt violated or protected during birth. Components of this measure could range from feeling embarrassed, scared, anxious, sad, out of control, ignored, vulnerable, dissociated, dehumanized, or traumatized to feeling seen, supported, and respected, deemed important, taken seriously, braced, empowered, confident, energetic, comfortable, and sheltered.

### Potential (mental) health effects of violated birth integrity

Supposing that preserved birth integrity builds a vital resilience for maternal health, simultaneously implicates that a violation of birth integrity may entail poor health outcomes in the short- or long run. As mentioned earlier, we did not integrate all the possible adverse health effects of birth integrity violations in our search. Nevertheless, we identified a few measures linking negative childbirth experiences to postpartum mental health conditions. These initial linkages between birth integrity violations and existing knowledge on traumatic birth experiences need to be systematically deepened. Research on traumatic birth experiences has so far focused on medically complicated deliveries or undesired health outcomes. We consider birth integrity violations as a critical vulnerability factor for developing birth-related post-traumatic stress disorders, postpartum depressions, or challenges in bonding and attachment of mother and child [123]. Mental or physical distress, a highly interventionist birth, or an unexpected course of childbirth can similarly cause trauma. Additionally, we see the necessity to research the physical and social consequences possibly deriving from birth integrity violations, like avoidance of subsequent pregnancies or impairments of sexual functions. Recently, a specific scale on postpartum, birth-related PTSD has been developed [124]. While there exist studies that demonstrate a significant association between birth experiences and postpartum depression [125], there is, to our knowledge, no scale that has operationalized a linkage between one’s birth perception (birth integrity) and postpartum depression. Understanding better if a violation of birth integrity can cause postpartum or birth-experience-related depression marks an important step towards recognizing, addressing, and improving mental postpartum health. Similarly, future research needs to better understand if and how violated birth integrity affects the bonding and attachment between the mother/parent and the child, the partnership, or negatively impacts the body functions.

### Implications for future research on birth integrity

The findings of this critical review (especially on the determinants of birth integrity) are closely related to the WHO recommendations on intrapartum care for positive childbirth experience [12]. However, both the WHO recommendations and the review results show that there is a lack of recognition that gender and power relations underlie the determinants of birth integrity (e.g., medical paternalism), birth integrity itself (e.g., gendered shame[126] or a normalization of violent acts against birthing bodies[127]), and are intertwined with the negative (mental) health consequences of motherhood myths (e.g., maternal guilt [128]). Conceptualizing research on birth integrity through a feminist research lens and methodology would not only make visible, analyze and tackle (gendered) health inequalities, but also contribute to a less hierarchical and more inclusive research process [129–131]. To do justice to the complexity and multidimensionality of the birth integrity framework in methodological terms, mixed-methods appear to be an appropriate approach, allowing a combination of positivistic and interpretivist concepts as well as triangulation of quantitative and qualitative data [132]. One way to prioritize feminist ethics at all stages of research is to adopt a participatory action approach [133].

### Strengths and limitations

This review constitutes a critical appraisal of quantitative epidemiological research conducted in the field of maternal care conditions, maternal care provision, childbirth experiences, and perceptions of birth. We developed a new multilevel birth integrity framework that not only presents an overview on what is currently included in the studies’ concepts and measurement (determinants, birth perception, consequence of birth integrity violations) but additionally identifies gaps that may be addressed in future studies. Gaining a fuller picture of the reasons that evoke suboptimal treatment and birth integrity violations becomes relevant when planning interventions or health policies.

Despite a thoughtful and transparent review process, some measures might have been missed. Also, we did not appraise the studies’ quality since we were not focused on the study findings. After reading all the full texts, we decided to exclude research on Quality of Care since these studies mainly assessed how maternity care was rated through rating scales (e.g., “How would you rate the care you received? – “Excellent”, “good”, etc.). To identify determinants of birth integrity, we found these kinds of survey questions less insightful. Considering the high volume of quality-of-care studies, we decided to focus on the remaining 82 studies more relevant to our review aims.

## Conclusions

The protection of birth integrity is an essential step towards respecting human rights in maternal health facilities globally. To ensure long-term maternal and postpartum health, additional interdisciplinary research, and various actors’ (practitioners, policymakers, legislation) collaborative engagement is needed. The multilevel birth integrity framework is a tool to analytically separate the complex and interwoven factors that can influence the birth situation and birth integrity. It can guide the development of survey instruments, qualitative interviews, or interventional studies.

Current epidemiological research on birth integrity measures many determinants related to the health system, the care culture, and rights in childbirth, mainly located on both meso-fields and the micro-manifest field. In this respect, the remaining macro-level fields seem under-studied and not sufficiently incorporated into quantitative health research. The inclusion of both macro-level measures and theoretical contributions, as well as qualitative insights from multidisciplinary perspectives (e.g., medical ethics, medical anthropology, psychology, sociology, gender studies, philosophy, or health economics) would allow to expand and enhance the multilevel birth integrity framework. To this end, a feminist research lens and mixed-method approach seem appropriate.

### Supplementary Information

**Additional file 1.** Search strategy, inclusion and exclusion criteria, and data extraction.

**Additional file 2.** eMERGE meta-ethnography reporting guidance.

**Additional file 3.** PRISMA 2020 flow diagram and adapted PRISMA for reporting systematic reviews reporting of qualitative and quantitative evidence.

**Additional file 4.** Overview on included studies’ characteristics.

**Additional file 5:** Process of identifying key concepts in conceptual clusters. **Table S1.** Disrespect and abuse (D&A), Mistreatment during facility-based childbirth (MisC). **Table S2.** Respectful maternity care (RMC). **Table S3.** Childbirth experiences (CE). **Table S4.** Maternal satisfaction (MS). **Table S5.** Obstetric violence. **Table S6.** Person-centered care (PCC).

**Additional file 6.** Measurement level: Process of identifying themes.

## Data Availability

All data generated or analysed during this study are included in this published article [and its supplementary information files].

## Abbreviations

WHO: World Health Organization
RMC: Respectful maternity care
D&A/MISC: Disrespect and abuse, mistreatment during facility-based childbirth
OV: Obstetric violence
PCC: Person-centered care
CE: Childbirth experiences
MS: Maternal satisfaction
