# Supplementary material for: A critical review: developing a birth integrity framework for epidemiological studies through meta-ethnography
Source: BMC Womens Health. 2023 Oct 10;23:530. doi: 10.1186/s12905-023-02670-z (PMC10565979; doi:10.1186/s12905-023-02670-z)
Supplement: Supplementary file 1 — Additional file 1. Search strategy, inclusion and exclusion criteria, and data extraction. [file 12905_2023_2670_MOESM1_ESM.docx]

## Additional file 1: Search strategy, inclusion and exclusion criteria, and data extraction

**Search strategy**

We developed the search strategy according to a modified PICO scheme (population, intervention, comparison and outcome). For P (Population), we subdivide the search terms into birth (e.g. “birth”, “delivery”, “labour”) and setting (e.g. “maternity ward”, “obstetric care”, “birth clinic”) and connected them by OR. We connected I (Intervention) and C (Comparison) by OR as well, containing search terms for the violations of maternal rights or deficient care (e.g. “birth violence”, “discrimination”, “disrespect”) and the protection of maternal rights or respectful maternal care provision (e.g. respectful maternity care, self-determination, autonomy, informed consent). Electronic searches were conducted in PubMed, PsychInfo, CINAHL and Embase. Studies we considered eligible for inclusion researched aspects directly relevant to the measurement of maternal care conditions, maternal care provision, birth experiences and perception of birthWe excluded studies assessing maternity care’s general quality, access to and utilization of maternity health care services or pregnancy care, and studies on violence outside the context of labour and birth (e.g. sexual abuse, domestic violence). Setting-wise, we included studies on facility-based childbirth (e.g. hospitals, obstetric clinics, birthing centres) and excluded studies on assisted and non-assisted home birth.

Concerning the population, we included people during childbirth and early postpartum phase and attendees of the birthing context (e.g. health care professionals, doulas, partners). We excluded studies on people using reproductive health services other than childbirth (e.g. fertility treatment, abortion). Primary quantitative studies of different types were included (e.g. cross-sectional, cohort-studies). In contrast, we excluded qualitative and mixed-methods studies and secondary research, reviews, editorials, commentaries or conceptual articles. The focus on quantitative studies serves the purpose to comprehend how maternal care provision, birth experiences the perception of birth has been operationalized and measured. We included studies aiming to validate instruments since these study types contain a new set of items. We excluded interventional studies since they build upon a prior research study using an equivalent set of items. We limited our scope on studies published between 2010-2020 since the thematic framework proposed by Bowser and Hill in 2010^[^[^19^](#_ENREF_19)^]^ led to a significant increase in research.

| Connector | Category | Connector | Sub-category | Terms |
| --- | --- | --- | --- | --- |
|  | Population |  | Birth | “birth” OR “childbirth” OR “delivery” OR “labor” OR “labor” |
|  |  | AND | Setting | “facility” OR “facilities” OR “clinic” OR “hospital” OR “maternity ward” OR “maternity care” OR “assisted delivery” OR “birthing center” OR “birth center” OR “birthing centre” OR “birth centre” OR “gynaecology” OR “gynecology” OR “obstetrics ward” OR “obstetric ward” “obstetric” OR “labor ward” OR “labor ward” OR “delivery room” OR “obstetric care” OR “obstetric delivery” |
| AND | Intervention/ Comparison |  |  |  |
|  |  |  | Violation of maternal rights | (“violence” OR “violation” OR “violate” OR “violating” OR “physical constraint” OR “moral constraint” OR “psychological constraint” OR “physical pressure” OR “moral pressure” OR “psychological pressure” OR “medicalization” OR “medicalisation” OR “discrimination” OR “disrespect” OR “disrespectful” OR “lack of respect” OR “marginalisation” OR “marginalization” OR “defamation” OR “abuse” OR “degrade” OR “harass” OR “harassment” OR “lack of privacy” OR “sexism” OR “racism” OR “classism” OR “bodism” OR “ageism” OR “stigmatization” OR “stigmatisation” OR “lack of privacy” OR “abandon” OR “over-use” OR “under-use” OR “instrumentalization” OR “instrumentalisation” OR “mistreatment” OR “dehumanisation” OR “dehumanization” OR “over-medicalization” OR “over-medicalisation” OR “under-medicalization” OR “under-medicalisation” OR “violence against woman” OR “obstetric violence” |
|  |  | OR | Maintainence of maternal rights | “informed consent” OR “respectful maternity care” OR “reproductive rights” OR “maternal rights” OR “bioethics” OR “human rights” OR “maternal treatment” OR “self-determination” OR “autonomy” OR “integrity” OR “dignity” OR “attentiveness” OR “empathy” OR “person-centered” OR “patient-oriented” OR “decision-making” OR “respectful” OR “quality of care” OR “maternal health” |

*Exemplary search strategy for pubmed.*

|  | Inclusion | Exclusion |
| --- | --- | --- |
| Terms and concepts | Epidemiological, quantitative studies on maternal care conditions, maternal care provision, birth experiences and perception of birth (common terms and concepts for this phenomenon are:  Obstetric violence, mistreatment, disrespect or abuse during childbirth, dehumanized childbirth, autonomy  Respectful maternity care, humanized childbirth, human rights in childbirth | Studies on violence, mistreatment or similar terms non-related to childbirth (e.g. domestic violence, sexual abuse, genital mutilation).  Studies on quality of care  Studies on particular interventions (e.g. epidural) or procedures (episiotomy), |
| Setting | Studies on facility-based childbirth in obstetric clinics or midwifery-guided birthing centers. | Studies on assisted or non-assisted homebirth. |
| Range of coverage | global |  |
| Population | People/ women during childbirth and postpartum phase | Woman using reproductive health services other than childbirth |
| Study types | Primary observational quantitative research studies (cross-sectional studies, cohort-studies, case-control-studies, ecological/correlation studies) | Primary research studies consisting of qualitative data or mixed method data. Secondary research, reviews, commentaries, editorials, conceptual articles, interventional studies, non-scientific literature- |
| Date | 2010-2020 | |

Inclusion and exclusion criteria

**Data extraction**

Pilot screening and pilot data extraction was conducted independently by three researchers on a sample of 10% of the included studies. CM and LW extracted 15 studies each for the final data extraction, SBZ extracted all studies. We extracted a variety of general information and information specifically relevant to the review's aim, e.g., terminology or concepts, definitional scope, theoretical reference, operationalization and measurement tools (including items) on maternal care provision, birth experiences and perceptions of birth. When the items were not reported, we contacted the authors and asked to send us the instruments they used (full data extraction format: example study).
