# Supplementary material for: A critical review: developing a birth integrity framework for epidemiological studies through meta-ethnography
Source: BMC Womens Health. 2023 Oct 10;23:530. doi: 10.1186/s12905-023-02670-z (PMC10565979; doi:10.1186/s12905-023-02670-z)
Supplement: Supplementary file 2 — Additional file 2. eMERGE meta-ethnography reporting guidance. [file 12905_2023_2670_MOESM2_ESM.docx]

**Additional file 2: eMERGE meta-ethnography reporting guidance**

| **The eMERGe meta‐ethnography reporting guidance (France et al., 2019) (where applicable)** | | | |
| --- | --- | --- | --- |
|  | Criteria Headings | Reporting criteria | Where to find: |
| **Phase 1—Selecting meta-ethnography and getting started** | | | |
| **Introduction** | | | |
| 1 | Rationale and context for the meta‐ethnography | Describe the gap in research or knowledge to be filled by the meta‐ethnography, and the wider context of the meta‐ethnography. | Page 3-4 |
| 2 | Aim(s) of the meta-ethnography | Describe the meta-ethnography aim(s). | Pages 5-6 |
| 3 | Focus of the meta‐ethnography | Describe the meta‐ethnography review question(s) (or objectives) | Page 4 |
| 4 | Rationale for using meta‐ethnography | Explain why meta‐ethnography was considered the most appropriate qualitative synthesis methodology | Page 5 |
| **Phase 2 – Deciding what is relevant** | | | |
| **Methods** | | | |
| 5 | Search strategy | Describe the rationale for the literature search strategy | Page 4-5 |
| 6 | Search process | Describe how the literature searching was carried out and by whom | Page 4-5, Appendix file A |
| 7 | Selecting primary studies | Describe the process of study screening and selection, and who was involved | Page 4-5, Appendix file A |
| **Findings** | | | |
| 8 | Outcome of study selection | Describe the results of study searches and screening | Page 6, Appendix file A + C |
| **Phase 3- Reading included studies** | | | |
| **Methods** | | | |
| 9 | Reading and data extraction approach | Describe the reading and data extraction method and processes | Appendix file A |
| **Findings** | | | |
| 10 | Presenting characteristics of included studies | Describe the characteristics of the included studies | Appendix file D |
| **Phase 4 – determining how studies are related** | | | |
| **Methods** | | | |
| 11 | Process for determining how studies are related | Describe the methods and processes for determining how the included studies are related: -Which aspects of studies were compared  **AND**  -How the studies were compared | Page 7, Table 1 |
| **Findings** | | | |
| 12 | Outcome of relating studies | Describe how studies relate to each other | Pages 7-9 |
| **Phase 5- Translating studies into one another** | | | |
| **Methods** | | | |
| 13 | Process of translating studies | Describe the methods of translation:   - describe the steps taken to preserve the context and meaning of the relationship between concepts within and across studies - describe how the reciprocal and refutional translations were conducted - Describe how potential alternative interpretations or explanations were consideres in the translations. | 9-12, Suppl. files E and F |
| **Findings** | | | |
| 14 | Outcome of translation | Describe the interpretative findings of the translation | 7-12 |
| **Phase 6 – Synthesizing translations** | | | |
| **Methods** | | | |
| 15 | Synthesis process | Describe the methods used to develop overarching concepts (“synthesised translations”).  Describe how potential alternative interpretations or explanations were considered in the synthesis | 12-15 |
| **Findings** | | | |
| 16 | Outcome of synthesis process | Describe the new theory, conceptual framework, model, configuration, or interpretation of data developed from the synthesis | 12-15 |
| **Phase 7 – Expressing the synthesis** | | | |
| **Discussion** | | | |
| 17 | Summary of findings | Summarize the main interpretative findings of the translation and the synthesis and compare them to existing literature. | Pages 16-19 |
| 18 | Strengths, limitations and reflexivity | Reflect on and describe the strengths and limitations of the synthesis:   - Methodological aspects— for example, describe how the synthesis findings were influenced by the nature of the included studies and how the meta-ethnography was conducted. - Reflexivity- for example, the impact of the research team on the synthesis findings | Page 19-20 |
| 19 | Recommendations and conclusions | Describe the implications of the synthesis. | Page 20 |

France, E.F., Cunningham, M., Ring, N., Uny, I., Duncan, E.A., Jepson, R.G., et al. (2019). Improving reporting of meta-ethnography: The eMERGe reporting guidance. *J Adv Nurs,* 75, 1126-1139.
