# Supplementary material for: A critical review: developing a birth integrity framework for epidemiological studies through meta-ethnography
Source: BMC Womens Health. 2023 Oct 10;23:530. doi: 10.1186/s12905-023-02670-z (PMC10565979; doi:10.1186/s12905-023-02670-z)
Supplement: Supplementary file 4 — Additional file 4. Overview on included studies’ characteristics. [file 12905_2023_2670_MOESM4_ESM.docx]

**Additional file 4: Overview on included studies’ characteristics**

| **Ref.** | **Title** | **Region** | **Study objective** | **Sample** | **Population** | **(Main) conceptual approach** |
| --- | --- | --- | --- | --- | --- | --- |
| (1) | Measuring mistreatment of women throughout the birthing process: implications for quality of care assessments | Kenya, Africa | To "describe how mistreatment of women and clinical process of care vary throughout the birthing process from admission, delivery and immediate post-partum care". | 677 | Women aged 15-45. | „Measures of mistreatment for this assessment were collected during the birthing process and categorised using the WHO typology at the analysis (…) stage. For example, three second order themes were assessed during admission: harsh language, lack of informed consent and lack of privacy. During delivery, four second order themes were examined: harsh language, use of force, unhygienic conditions (these were defined as the basic requirement a provider must adhere to as part of broader infection control practices regard- less of level of care), and lack of privacy. During immediate postpartum care, three second order themes were assessed: unhygienic conditions, lack of privacy and lack of informed consent”. |
| **Items**: 1. During your time in the health facility did the doctors, nurses, or other health care providers introduce themselves to you when they first came to see you? 2. Did the doctors, nurses, or other health care providers call you by your name? 3. Did the doctors, nurses, or other staff at the facility treat you with respect?4. Did the doctors, nurses, and other staff at the facility treat you in a friendly manner? 5. Did you feel the doctors, nurses, or other health providers shouted at you, scolded, insulted, threatened, or talked to you rudely? 6. Did you feel like you were treated roughly like pushed, beaten, slapped, pinched, physically restrained, or gagged? 7. During examinations in the labor room, were you covered up with a cloth or blanket or screened with a curtain so that you did not feel exposed? 8. Do you feel like your health information was or will be kept confidential at this facility? 9. Did you feel like the doctors, nurses or other staff at the facility involved you in decisions about your care? 10. Did the doctors, nurses or other staff at the facility ask your permission/consent before doing procedures on you? 11. During the delivery, do you feel like you were able to be in the position of your choice? 12. Did the doctors, nurses or other staff at the facility speak to you in a language you could understand? 13. Did the doctors and nurses explain to you why they were doing examinations or procedures on you? 14. Did the doctors and nurses explain to you why they were giving you any medicine? 15. Did you feel you could ask the doctors, nurses or other staff at the facility any questions you had? 16. Did the doctors and nurses at the facility talk to you about how you were feeling? 17. Did the doctors, nurses or other staff at the facility try to understand your anxieties? 18. When you needed help, did you feel the doctors, nurses or other staff at the facility paid attention? 19. Do you feel the doctors or nurses did everything they could to help control your pain? 20. Were you allowed to have someone you wanted (outside of staff at the facility, such as family or friends) to stay with you during labor? 21. Were you allowed to have someone you wanted to stay with you during delivery? 22. Do you think there was enough health staff in the facility to care for you? 23. Did you feel the doctors, nurses or other staff at the facility took the best care of you? 24. Did you feel you could completely trust the doctors, nurses or other staff at the facility with regards to your care? 25. Thinking about the labor and postnatal wards, did you feel the health facility was croweded? 26. Thinking about the wards, washrooms and the general environment of the health facility, will you say the facility was very clean, clean, dirty, or very dirty? 27. Was there water in the facility? 28. Was there electricity in the facility? 29. In general, did you feel safe in the health facility? 30. How did you feel about the amount of time you waited? | | | | | | |
| (2) | Predictors of person-centered maternity care: the role of socioeconomic status, empowerment, and facility type | Kenya, Africa | To " to examine factors associated with PCMC, particularly the role of household SES, personal empowerment, and type of delivery facility". | 877 | Women aged 15-49 (up to 9 weeks postpartum) | “Person-centered maternity care (PCMC) refers to “maternity care that is respectful of and responsive to individual women and their families’ preferences, needs, and values” (…). The WHO recommendations highlight respectful maternity care, effective communication, and companionship during labor and childbirth as key dimensions of PCMC that should be provided to every women throughout labor and birth (…).” |
| (3) | Validation of the person-centered maternity care scale in India | India, Asia | To "present the results of the psychometric analysis of the same tool validated by Afulani et al. 2017 in Kenya in an Indian population". | 2018 | Women aged 18 to 46 up to 48 h postpartum. |  |
| **Items:** 1. During your time in the health facility did the doctors, nurses, or other health care providers introduce themselves to you when they first came to see you? 2. Did the doctors, nurses, or other health care providers call you by your name? 3. Did the doctors, nurses, or other staff at the facility treat you with respect?4. Did the doctors, nurses, and other staff at the facility treat you in a friendly manner? 5. Did you feel the doctors, nurses, or other health providers shouted at you, scolded, insulted, threatened, or talked to you rudely? 6. Did you feel like you were treated roughly like pushed, beaten, slapped, pinched, physically restrained, or gagged? 7. During examinations in the labor room, were you covered up with a cloth or blanket or screened with a curtain so that you did not feel exposed? 8. Do you feel like your health information was or will be kept confidential at this facility? 9. Did you feel like the doctors, nurses or other staff at the facility involved you in decisions about your care? 10. Did the doctors, nurses or other staff at the facility ask your permission/consent before doing procedures on you? 11. During the delivery, do you feel like you were able to be in the position of your choice? 12. Did the doctors, nurses or other staff at the facility speak to you in a language you could understand? 13. Did the doctors and nurses explain to you why they were doing examinations or procedures on you? 14. Did the doctors and nurses explain to you why they were giving you any medicine? 15. Did you feel you could ask the doctors, nurses or other staff at the facility any questions you had? 16. Did the doctors and nurses at the facility talk to you about how you were feeling? 17. Did the doctors, nurses or other staff at the facility try to understand your anxieties? 18. When you needed help, did you feel the doctors, nurses or other staff at the facility paid attention? 19. Do you feel the doctors or nurses did everything they could to help control your pain? 20. Were you allowed to have someone you wanted (outside of staff at the facility, such as family or friends) to stay with you during labor? 21. Were you allowed to have someone you wanted to stay with you during delivery? 22. Do you think there was enough health staff in the facility to care for you? 23. Did you feel the doctors, nurses or other staff at the facility took the best care of you? 24. Did you feel you could completely trust the doctors, nurses or other staff at the facility with regards to your care? 25. Thinking about the labor and postnatal wards, did you feel the health facility was croweded? 26. Thinking about the wards, washrooms and the general environment of the health facility, will you say the facility was very clean, clean, dirty, or very dirty? 27. Was there water in the facility? 28. Was there electricity in the facility? 29. In general, did you feel safe in the health facility? 30. How did you feel about the amount of time you waited? | | | | | | |
| (4) | Toward the development of a short multi-country person-centered maternity care scale | Kenya, Ghana, Africa, India, Asia | To "develop a shorter, more simplified PCMC tool that could be applied by program implementers across multiple settings". | 3663 (1419 from Kenya, 226 from Ghana, 2018 from India) | Women aged 15–49 years, up to 9 weeks postpartum |  |
| **Items**: Short scale of PCMC: 1. Did the doctors, nurses or other healthcare providers call you by your name? 2. Did the doctors, nurses or other staff at the facility treat you with respect? 3. Did the doctors, nurses or other staff at the facility treat you in a friendly manner? 4. During examinations in the labor room, were you covered up with a cloth or blanket or screened with a curtain so that you did not feel exposed? 5. Did you feel like the doctors, nurses or other staff at the facility involved you in decisions about your care? 6. Did the doctors, nurses or other staff at the facility ask your permission/consent before carrying out procedures and examinations? 7. During the delivery, do you feel like you were able to be in the position of your choice? 8.Did the doctors and nurses explain to you why they were carrying out examinations or procedures? 9. Did the doctors and nurses explain to you why they were giving you any medicine? 10. Did you feel you could ask the doctors, nurses or other staff at the facility any questions you had? 11. Did the doctors and nurses at the facility talk to you about how you were feeling? 12. When you needed help, did you feel the doctors, nurses or other staff at the facility paid attention? 13. Did you feel the doctors, nurses or other staff at the facility took the best care of you? | | | | | | |
| Alvares et al. (2018) (5) | Humanized practices of obstetric nurses: contributions in maternal welfare | Brazil, South America | To "analyze the practice of obstetrical nurses operating in a prenatal/de- livery/postpartum unit of a university hospital in Mato Grosso and the maternal welfare resulted from the care provided in this scenario". | 104 | Women with vaginal deliveries, conducted by both a doctor and an ostetric midwife, 4 hours in delivery ward | Not applicable |
| **Items**: Domain I – Quality of relationship during care, Domain II – Self-care and comfort, Domain III – Conditions that allow the contact between mother and child, Domain IV - Depersonalized Care, Domain V – Continuous participation of the family, Domain VI – Timely and respectful care, Domain VII – Comfortable physical environment | | | | | | |
| (5) | Exposure to verbal abuse and neglect during childbirth among Jordanian women | Jordan, Asia | To "assess the prevalence of provider verbal abuse and neglect towards women during childbirth in Jordanian healthcare settings". | 390 | 390 women aged 18-45 who had given birth vaginally to a alive child within the last 3 months and were able to read and write arabic, no serious illness during pregnancy, no high-risk-pregnancies. | “Women's relationship with health care providers in maternity settings during childbirth significantly impacts their physical, psychological, and emotional health during childbirth. (…) An important, but little understood component of the poor quality of care experienced by women during childbirth in facilities is disrespectful and abusive behavior by health care professionals and other facility staff”. |
| **Items**: Childbirth verbal abuse and neglect scale (CVANS): 1. I received a safe care for me and my child, 2. The health team gave me all possible care and attention, 3. I felt that some medical mistakes occurred while providing care to me, 4. the health-care team was well trained to care for me, 5. the health-care team took into consideration my feelings and circumstances, 6. The health-care team took very good care of me and gave me all the attention I need during childbirth, 7. The health-care team did what they could to mediate my labor pain, 8. There were times that no one described what was happening to me, 9. I was provided with all new information about the development of my childbirth, 10. The health-care team stayed with me all the time and when I needed them, 11. I was able to see the doctor any time I needed him/her, , 12.The health-care team made this experience of childbirth a good one for me, 13. The health-care team protected my privacy during childbirth, 14. I received psychological and emotional support from the health-care team during labor, 15. I felt scared during the childbirth process , 16. The health-care team refused to give me pain killer, 17. I Have been well covered by sheets while in labor process especially during vaginal examination, 18. Some health care providers answered my questions angrily while in labor, 19. The health care provider refused to assist me during or after giving birth, 20. While still in the labor room and after I gave birth I received a medical checkup or health care assessment, 21. I was asked how I have been doing after childbirth by the health care providers in labor room, 22. I received pain killers during stitching, 23. I still could feel pain during stitching even though I was given pain killers / anesthesia. Verbal abuse scale: 1. Has any one of the health care team shouted at you while in labor?, 2. Did any one of the health care team use an aggressive tone of voice with you during childbirth? , 3. Has any one of the health care team used verbal threats such as (if you do not push I will give you a C- section, if you do not push good, I will make you a vaginal cut)., 4. Has any one of the health care team used swearing or harsh words while you were in labor such as (you are stupid, You know nothing about labor)., 5. Has any one of the health care team treated you roughly (rude/unkind) while you were in labor? , 6. Has any one of the health care team ridiculed you while were in labor such as (stop pretending you are in pain, I am not the one that made you pregnant)., 7. Has any one of the health care team gave you incorrect or withhold information about your medical condition during childbirth? 8. Has any one of the health care team called you names during childbirth? | | | | | | |
| (6) | Service providers' experiences of disrespectful and abusive behavior towards women during facility based childbirth in Addis Ababa, Ethiopia | Ethiopia, Africa | To "generate evidence on service providers’ experiences of D&A during childbirth". | 57 | Health professionals (midwives, clinical nurses, health officers, and medical doctors) working on the labor ward in one of the four health institutions. Minimum service of six consecutive months in the labor room (prior to data collection phase). | “(…) interactions or facility conditions that local consensus deems to be humiliating or undignified, and those interactions or conditions that are experienced as or intended to be humiliating or undignified (…)” |
| Service providers’ practices related to D&A in light of RMC: Do service providers, in this facility 1) Provide appropriate pain relief or comfort measures for laboring mothers? 2) Introduce themselves to laboring mothers? 3) Encourage a mother’s companion to remain with her, whenever possible? 4) Convey information to mothers at a language-level they can understand? 5) Encourage mothers to ask questions during labor? 6)Respond to mothers’ questions with promptness, politeness, and truthfulness? 7) Explain to mothers what is being done and what to expect throughout labor and birth? 8)Provide periodic updates on status and progress of labor to laboring mothers? 9) Allow mothers to move around during labor? 10) Allow mothers to assume the position of her choice during birth? 11) Obtain consent or permission of mothers prior to any procedure? 12) Use curtains or other visual barriers to protect mothers during exams, births and procedures? 13)Are mothers encouraged to call for help if they are in need? 14) Is it easy for service providers to respond to mothers’ calls for help? Service providers’ personal observations of disrespectful and abusive care during childbirth in health facilities): 1) Physical force or abrasive behavior with laboring mothers (for example slapping or hitting them), 2) Mothers have been separated from their baby unnecessarily, 3) Mothers have been denied foods or fluids unnecessarily, 4) Service providers have used insults, intimidation, threats, or coercion to mothers or their companion, 5) Service providers shown disrespect to mothers based on any specific attribute. (HIV status or ethnic group, for example), 6) Mothers have been left alone or unattended, 7) Mothers’ privacy during labor and delivery has not been protected, 8) Mothers have been detained at the facility, against their will, 9) In your own personal capacity have you ever done anything that made you feel you disrespected or abused women in childbirth?, 10) Service providers’ perceptions of the consequences of disrespectful and abusive care during childbirth, 11) Do you think you have ever been disrespected or abused at your work place by anyone? 12) Do you think lack of respectful care is a factor which discourages pregnant women from coming to health facilities for delivery? | | | | | | |
| (7) | Status of respectful and non-abusive care during facility-based childbirth in a hospital and health centers in Addis Ababa, Ethiopia | Ethiopia, Africa | To "quantitatively determine the level and types of disrespect and abuse women face during facility-based childbirth and report on subjective experiences of disrespect and abuse". | 173 | Women who had given birth vaginally. | “ (…) 7 categories of disrespect and abuse during childbirth: physical abuse, non-consented care, non-confidential care, non-dignified care, discrimination based on specific patient attributes, abandonment of care, and detention in facilities (…)”. |
| **Items:** (C) = Catgeory of D&A, (T)= Type of D&A: The woman is protected from physical harm or ill treatment (C): The provider used physical force /slapped me/hit me (T), I was physically restrained (T), I was separated from my baby without medical indication (T), I was denied food or fluid in labor unless medically necessitated (T).The woman’s right to information, informed consent, and choice/preferences is protected (C): The provider did not introduce himself/herself to me and my companion (T), The provider did not encourage me to ask questions (T), The provider did not respond to my questions with promptness, politeness, and truthfulness (T), The provider did not explain to me what is being done and what to expect throughout labor and birth (T), The provider did not give me periodic updates on status and progress of my labor (T), The provider did not allow me to move about during labor (T), The provider did not allow to assume position of choice during birth (T), The provider did not obtain my consent or permission prior to any procedure (T), I did not receive comfort/pain-relief as necessary (T), The providers did not demonstrate caring in a culturally appropriate way (T).The woman’s confidentiality and privacy is protected. (C): The provider did not use curtains or other visual barriers to protect me (T), The woman is treated with dignity and respect. (C), The provider did not speak to me politely (T), The provider made insults, intimidation, threats, or coerced me (T). The woman receives equitable care, free of discrimination (C): The provider spoke to me in a language and at a language-level that I cannot understand (T), The provider showed disrespect to me based on any specific attribute (T). The woman is never left without care/ attention (C): The provider did not encourage me to call if needed (T), The provider did not come quickly when I called him/her (T), The provider left me alone or unattended (T), The woman is never detained or confined against her will (C), I was detained in health facility against my will (T) | | | | | | |
| (8) | Patient-reported Communication Quality and Perceived Discrimination in Maternity Care | United States of America, North America | To "examine whether there are racial/ethnic disparities in patient-provider communication quality during prenatal care and perceived discrimination during birth hospitalizations in a national sample; and to explore potential racial/ethnic variation in the sociodemographic and health-related correlates of these outcomes". | 2400 | Women aged 18-45, who gave birth in a US hospital 2010 | “PCC, defined as ‘care that is respectful of and responsive to individual patient preferences, needs, and values and ensuring that patient values guide all clinical decisions’ (…)Aspects of patient-centered care such as patient-provider communication and patient involvement in decision making are associated with higher levels of patient satisfaction, more trust in the provider, and better treatment adherence (…); in some studies patient-centered care is also associated with better health outcomes (…)”. |
| **Items:** Domain: Perceived discrimination:During your hospital stay when you had your baby, how often were you treated poorly because of: Your race, ethnicity, cultural background or language?Your health insurance situation?A difference of opinion with your caregivers about the right care for yourself or your baby? | | | | | | |
| (9) | Declined care and discrimination during the childbirth hospitalization | United States of America, North America | To "investigate women's experiences of de- clining procedures in maternity care. Specifically, we examined the association between women's reports of declining medical procedures and perceived discrimination. Further, we assessed whether declining procedures was differently associated with perceived discrimination depending on the woman's race/ethnicity". | 2400 | Women aged 18-45, who gave birth in a US hospital 2010 | „ (…) patient-centered care has been held up as the ideal model of patient-provider interaction in all types of health- care; the implementation of patient-centered care is now recognized as an integral component of care quality (…). In an approach consistent with patient-centered care, clinicians respect and take into account individual patients’ preferences and values, and involve patients in decision-making (…). Along with this shift toward patient-centered care, patients are increasingly viewed as consumers (...). In this model, healthcare providers are charged with providing adequate information to patients to enable them to make decisions that best fit their preferences, while patients are charged with active involvement in making decisions about their treatment and following through to implement treatment plans (…)” |
| **Items:** Domain: Perceived discrimination:During your hospital stay when you had your baby, how often were you treated poorly because of: Your race, ethnicity, cultural background or language?Your health insurance situation?A difference of opinion with your caregivers about the right care for yourself or your baby? | | | | | | |
| (10) | Disrespect and abuse during childbirth in district Gujrat, Pakistan: A quest for respectful maternity care. | Pakistan, Asia | To "assess the prevalence and determinants of the disrespect and abuse during child birth in rural Gujrat, Pakistan". | 306 | Women aged 17-38 years having a life baby and a delivery without general anasthesia (who delivered during last two months) | Women experience ill treatment not only in violation of their autonomy and dignity but also as verbal insults, humiliation, discrimination, abandonment of care and physical assault during childbirth. (…) formally called these maltreatments Disrespect and Abuse (D&A) during childbirth and highlighted this as a main factor in the underutilization of health care facilities. Although an objective assessor reviewing statements about a woman’s experience during labor and birth may see that she has been a victim of D & A “experienced D & A”, the woman herself may not recognize that this was D & A “reported D & A. |
| **Items:** Non-consented care: Provider did not introduce herself, Provider did not encourage to ask questions, Provider didn’t responded politely, truthfully and promptly, Provider didn’t explained procedure and explained expectations, Provider didn’t give the periodic updates on status and progress, Provider didn’t give the periodic updates on status and progress, Provider didn’t allow to move during delivery, Provider didn’t allow to assume position of choice, Provider didn’t obtain consent prior to procedure Non confidential care: Curtains and physical barriers were not used, Drape or body covering was not used, The number of staff members around were not logical. Non-dignified care: Provider didn’t speak politely, Provider made insults, threats etc., Provider used abusive language. Discriminatory care: Provider used language difficult to understand, Provider showed disrespect based on specific attribute. Abandonment in facility: Provider didn’t encouraged to call if needed, Provider made patient feel alone or unattended,Provider didn’t come quickly when needed. Physical abuse: Provider used physical force, slapped or hit the woman, Woman was physically restrained, Baby was separated without medical indication, Didn’t receive comfort, pain relief as necessary. Provider didn’t demonstrated in culturally appropriate way. Detention in facility. | | | | | | |
| (11) | Development and use of a scale to assess gender differences in appraisal of mistreatment during childbirth among Ethiopian midwifery students | Ethiopia, Africa | To "develop a tool that assesses mistreatment appraisal from a provider’s perspective and to assess gender dif- ferences in mistreatment appraisal among Ethiopian final-year midwifery students and to analyze possible mediating roles of self-esteem and stress". | 390 | final year midwifery students (151 males, 239 females) | “Mistreatment comprises (…) seven domains: 1. physical abuse, such as slapping, 2. sexual abuse, such as rape, 3. verbal abuse, such as shouting, 4. stigma and discrimination, such as providing poor treatment due to HIV status, 5. failure to meet professional standards, such as neglect, 6. poor rapport between women and providers, such as dis- missal of women’s concerns, and 7. health system conditions and constraints, such as lack of privacy. Mistreatment is often justified as a means of punishment for patients’ misbehavior (…)“. |
| **Items:** Mistreatment-Appraisal-Scale (10-item MISAP Scale): 1. A woman is constantly closing her legs during the second stage of labor. A midwife tells her that she should not do that, as he/she believes that the baby will not deliver, yet the woman continues to move her legs together. Each time the woman closes her legs, the midwife slaps her legs and forces them apart again. 2. An episiotomy (surgical cut at opening of vagina) is performed at an obstetric health facility due to fetal distress. The woman is illiterate and comes from a rural area. The midwife believes that the woman will not understand the medical procedure and that offering explanations would be a waste of time. In order to quicken childbirth, the midwife carries out the episiotomy without any explanation and getting the woman’s permission. 3. On a busy day, the admission room of a district hospital is overcrowded with many emergency cases. During the admission of one of the cases – a woman in advanced labor who has vaginal bleeding and is very anxious – the midwife does not offer explanations about what he/she is doing or any findings on the procedures to the woman and her sister who accompanied her.. 4. A midwife is caring for a woman in labor who is HIV positive. In order to limit the risk of infection, the midwife believes she needs to tell the woman’s HIV status to a colleague, who works at the outpatient department and who is not directly involved in the care of that woman during lunch break in the cafeteria. 5. A 16-year-old girl is visiting a health facility for safe abortion care at 10 weeks in a district hospital. Before the procedure is carried out, the girl is asked whether she has ever had an abortion. The girl indicates that she has had two abortions within the last two years, due to poor contraceptive use. During the procedure, she is weeping loudly, despite receiving painkillers. A midwife tells her in a strict tone: ‘If you find the procedure painful, stop sleeping with a man at every opportunity you get!’ 6. A midwife that works at a health center provides all aspects of maternity care services. There are many women waiting for their turn. Some of the clients are from the same area as the midwife. Some women who speak a different language arrived at the facility earlier, yet the midwife attends to the women from her area first. 7. A midwife believes that if companions are allowed in the delivery room, the cleanliness of the room will be compromised, which poses a risk to the health of newborns. Moreover, the midwife thinks that companions reduce patients’ privacy, given the fact that multiple women give birth in the same room. A woman asks if her mother can be present during her delivery but the midwife denies this request for the above-mentioned reasons. 8. A mother with postpartum bleeding arrives at a health facility following a home birth. She has lost about 400 ml of blood within the first 24 hours following childbirth and is unwell. She waits two hours for a midwife that works alone to finish attending to two births. After the deliveries, the midwife is exhausted and wants to go home as the scheduled shift is over. Therefore, the midwife decides to refer the woman to another health facility, which is one hour away. 9. A woman wants to give birth in a kneeling position, however a midwife believes that the bed is not suited for that position. Hence, the midwife forces the woman to give birth in a lying position. 10. A midwife attends to a woman that came for delivery services to a district hospital. Other women are also being attended to in the same room. The woman is shouting and crying and others feel disturbed. The healthcare personnel are finding it hard to concentrate when carrying out routine tasks. A midwife tells the woman to be quiet, yet the woman continues to make a lot of noise. Eventually, the midwife yells at the woman to be quiet using very harsh language. | | | | | | |
| (12) | Jeopardizing quality at the frontline of healthcare: prevalence and risk factors for disrespect and abuse during facility-based childbirth in Ethiopia. | Ethiopia, Africa | To "quantify and the frequency and categories of D&A experienced by women in four health centres n two rural regions of Ethiopia". | 193/204 (observations/interviews) | All women who gave birth in one of the four health care centres. | “D&A has been acknowledged as a deficiency in the delivery of high quality maternal health services, threatening the ability of health systems to achieve good maternal health outcomes (…). D&A manifests as physical violence, harsh language, stigma and neglect suffered by women at the hands of health care providers (…). Drivers of D&A can include systemic failures, such as overwhelmed health care administration, poor staffing and supervisory structures and inadequate physical infrastructure (…). Women who experience D&A are more likely to report lower satisfaction with their birth experience and are less likely to seek facility-based delivery for future pregnancies (…)”. |
| **Items**: Observations of disrespect and abuse (1st and 3rd order themes) (Items for Reported experiences of D&A not reported):Physical abuse (3rd): fundal pressure applied (1st) Non-consented care(3rd): Lack of consent for first vaginal examination (1st) Non-confidential care (3rd): Mother’s history taking findings shared when others could hear (1st),auditory privacy not respected during post-natal examination (1st) Lack of privacy(3rd): No partitions separating beds for first examination (1st), Partitions do not give privacy in prenatal ward (1st), Mother not covered during examination in prenatal ward (1st), Mother not covered while being moved from prenatal ward to delivery room (1st) Mother not covered during delivery (1st), Partitions not closed during delivery (1st), Mother not well covered after third stage of labour (1st), No partitions/curtains between beds in post-natal ward (1st), Mother’s physical privacy not respected during post-natal examination(1st). Non-dignified care(3rd): mother not welcomed in a kind and gentle maner(1st); provider did not introduce herself to mother (antenatal ward) (1st), use of non-dignified language during history taking(1st); delivery midwife did not introduce herself by name(1st); delivering service provider did not congratulate mother after birth(1st); mother not cleaned after birth and third stage of labour(1st); no pad provided to mother(1st); mother not allocated her own bed inpost-natal ward(1st), bed in post-natal ward not clean(1st); mother not called by her name throughout interactions(1st); mother not asked about preferred birth position (1st); mother not allowed to practice religious/cultural custom, if requested(1st); | | | | | | |
| (13) | Respectful maternity care and associated factors among women who delivered at Harar hospitals, eastern Ethiopia: a cross- sectional study | Ethiopia, Africa | To "assess the status of RMC and associated factors at Harar town hospitals, Eastern Ethiopia". | 425 | Women who visited the nominated Harar hospitals for labor and delivery throughout the information assortment period were included. Women who were fundamentally sick, and unfit to impart were excluded from the study. | “Respectful maternity care (RMC) during childbirth is an interaction between the client and the healthcare providers (HCPs) or facility conditions. It has a significant role in maternal mortality ratio reduction by enhancing clients’ inclination to deliver in health facilities. Furthermore, RMC is the standard of care for all women that encompasses women’s basic human rights”. |
| **Items:** Friendly care: I felt that healthcare workers cared for me with a kind approach.Healthcare workers treated me in a friendly manner. The healthcare providers were talking positively about pain and relief. The health worker showed his/her concern and empathy. All healthcare workers treated me with respect as an individual. The healthcare workers speak to me in a language that I can understand. The healthcare providers called me by my name. Abusive free care: The healthcare workers responded to my needs whether or not I asked. Some healthcare providers slapped me during delivery for different reasons. Some health workers shouted at me because I haven’t done what I was told to do. Timely care: Some healthcare providers slapped me during delivery for different reasons. Discrimination-free care: Some of the health workers do not treat me well because of some personal attribute.Some health workers insulted me and my companions due to my personal attributes. I was allowed to practice cultural rituals in the facility. | | | | | | |
| (14) | Is there respectful maternity care in Poland? Women’s views about care duringlabor and birth | Poland, Europe | To "analyze perinatal care related experiences of women, especially focusing on those that have characteristics that indicate disrespectful/abu- sive care during childbirth in health facilities". | 8378 | Women who had given birth in 2017 or 2018 to an alive baby. | “A systematic review in the area of negligence and violations of childbirth led by Bohren et al. allowed a widening to the typology of these abuses (…). The review presented a detailed typology that was evidence based and comprehensively illustrated how women in perinatal care facilities can be mistreated on multiple levels: inter- actions between women and healthcare providers as well as system and organizational failures (…)“. |
| **Items:** Verbal violence: Inappropriate comments, Nonchalant treatment, Not answering questions/ignoring, Raising your voice, shouting, disrespectful expressions, Mocking, Insulting, Blackmailing with child’s health / woman’s health. Physical violence: The staff would force their legs apart when pushing, The staff tied their legs to the delivery bed, The staff poked her. Abuse doing things without asking women for permission (lack of consent): Enema, Newborn vaccination, Shaving of pubic hair, Newborn examination, Induction of delivery, Administration of an oxytocin drip, Episiotomy, Vaginal examination, Newborn drug administration, Insertion of intravenous cannula, Feeding a newborn baby with modified milk, Presence of students during delivery,. Newborn bath. No access to professional health care: Undelicate treatment (internal examination, episiotomy repair), No access to lactation consultant, No support in breastfeeding, No support in dealing with depressed mood, No access to epidural anesthesia.Care that violates the right to privacy/confidentiality: Some activities were done without respect for intimacy, Improper relations between staff and women, Providing information in an incomprehensible way, Not showing respect, Conversation in a rude and uncultured manner, Not giving all the information needed, Discrimination and stigmatization, The feeling of being discriminated or stigmatized | | | | | | |
| (15) | Respectful and disrespectful care in the Czech Republic: an online survey. | Czech Republic, Europe | To "obtain maternity care-givers’ views on levels of respectful care, and their perception of the prevalence of potentially disrespectful interventions and care in the Czech Republic, in an attempt to supplement the sparse existing data". | 52 | Midwifes, doulas and birth counsellor | “Respectful maternity care encompasses physical and psychological care, communication and interactions, is influenced by structural, organisational and cultural systems, and financial issues and implies 'doing no harm'. The terms used to describe respectful care include both positive descriptions, such as ‘respectful’ and ‘humanised’, and negative descriptions, such as ‘disrespectful’, ‘obstetric violence’, ‘mistreatment’ and ‘abuse’”. |
| **Items:** Hospital´s (or home birth) rates of: induction, acceleration of labour, episiotomy and third/ fourth perineal tears, mode of birth, frequency of neonatal birth-related injuries; Hospital’s (or home birth) practices on: offering women choice regarding induction of labour or elective caesarean section, electronic fetal monitoring, the use of local anaesthetic before suturing perineal trauma, umbilical cord clamping and cutting, skin-to-skin (SCC) contact, rooming-in and non-separation of mother and baby; Women’s ability to move around freely during labour, positions for spontaneous vaginal birth, the practice of pushing hard on the woman’s abdomen, and/or pulling on the baby’s head, during the birth; Whether or not women are informed of the reason why vaginal examination and artificial rupture of membranes are performed, if their permission is sought, and professionals’ reactions when women refuse; If women in normal labour, with no risk factors, are permitted to drink fluids and eat a light diet in labour. | | | | | | |
| (16) | Magnitude of disrespectful and abusive care among women during facility-based childbirth in Shambu town, Horro Guduru Wollega zone, Ethiopia | Ethiopia, Africa | To "determine the levels and types of D&A care during facility-based childbirth among women in Shambu town and ist determinants". | 321 | All mothers who gave birth at the public health facilities of Shambu town during twelve months of preceding the study. | Not applicable |
| **Items:** Physical abuse: health provider hit or slapped, forcing leg apart during labor, provider insulted or treatened me and/or my companion; did not receive a comfortable/pain-relief treatment, denied from food or fluid in labor unless medically necessitated. Privacy not maintained: did not us drapes or cover to keep privacy, health provides discussed your private health information in a way that others could hear. Non-consented care: Provider did not introduce herself, Provider did not encourage to ask questions, Provider didn’t responded politely, truthfully and promptly, Provider didn’t explained procedure and explained expectations, Provider didn’t give the periodic updates on status and progress, Provider didn’t give the periodic updates on status and progress, Provider didn’t allow to move during delivery, Provider didn’t allow to assume position of choice, Provider didn’t obtain consent prior to procedure. Non-dignified care: health provider shouted at me, health providers made negative comments about me, delivery coach on which I gave birth was not clean. Abandonment/neglect of care: Provider ignored me or did not come quickly when I called him/her, provider left me alone, or unattended; discriminated while receiving care, providers discriminated by ecnonmic status. Experienced detention: kept in health facility without her will. | | | | | | |
| (17) | Silent voices: Institutional disrespect and abuse during delivery among women of Varanasi district, northern India. | India, Asia | To "assess the frequency and nature of disrespect and abuse experienced in health care facilities by women, and explore possible associations, if any during labour and delivery". | 410 | Women from all age groups who had delivered at any government or a private health facility between June 2014 to August 2015. | “Bowser and Hill conducted a land- scape analysis identifying seven categories of disrespect and abuse: physical abuse, non-consented care, non-confidential care, non-dignified care, discrimination based on specific patient attributes, abandonment of care, and detention in facilities due to failure to pay (…)“. |
| **Items:** Physical abuse: slapping/ pinching etc., use of excessive force during delivery, delivery without any pain relief. Non-dignified care: shouting/ scolding; threatening to withhold treatment; threatening or negative comments. Non-confidential care: delivery without any physical barriers, disclosing private health information to others. Neglect: ignored when needed help, delivery without attendant. Inappropriate demands for money: Detention in facility for failure to pay, request for bribe | | | | | | |
| (18) | Maternal Satisfaction on Delivery Service and Its Associated Factors among Mothers Who Gave Birth in Public Health Facilities of Debre Markos Town, Northwest Ethiopia. | Ethiopia, Africa | To assess "satisfaction on delivery service and its associated factors among mothers who gave delivery in public health facilities of Debre Markos town". | 398 | All women who used delivery services at a public health institution within the past one year of study conduction | “Patient satisfaction is a subjective and dynamic perception of the extent to which the expected health care is received (...). It is not important whether the patient is right or wrong, but what is important is how the patient feels”. |
| **Items:** Physical and staff accessibility: Examination area cleanliness and comfort,Restfulness of the rooms of the facility, Overall cleanness of the facility, Availability of transportation, Supplies of basic drugs and equipment, Accessibility and cleanness of toilets and/or shower, Waiting time to be seen by health worker.Interaction with providers and staff of the facility: Helpfulness of staff, Explanation about the treatment given,Communication between health care providers, Opportunity to ask about the treatment, Involvement of patient in decision making, Willingness of staff to hear patient problems, Explanation about the drugs prescribed, Explanation about the side effects of drugs, Delivery position of patient choice. Provision of respect and privacy: Respect and assurance of privacy, Respect of social norms and values, Equal treatment of people | | | | | | |
| (19) | How women are treated during facility-based childbirth in four countries: a cross-sectional study with labour observations and community-based surveys. | Ghana, Guinea, Myanmar, and Nigeria (Africa, Asia) | To "develop and implement evidence-informed, validated tools to measure mistreatment during childbirth, and report results from a cross-sectional study in four low-income and middle-income countries". | 2016 for observation, 2672 for survey | Labour observation: women admitted for childbirth in early established or active labour , aged at least 15 years. Survey: admitted for childbirth, were aged at least 15 years. | “Evidence suggests that women across the world experience mistreatment during childbirth, including physical abuse, verbal abuse, discrimination, non-consented procedures, and non-supportive care (…). Bowser and Hill’s landscape analysis (…) brought this issue to global attention and our mixed-methods systematic review developed a typology of what constitutes mistreatment”. |
| **Items:** Mistreatment (labour observations and survey): Any physical abuse:Slap, Forceful downward pressure on abdomen, Held down to the bed forcefully, Punch, Hit, Kick, Pinch, Gag, Tied to the bed, Other physical abuse, Verbal abuse: Shouted at, Scolded, Mocked, Insulted, Threatened with poor outcome for baby, Hissed at, Negative comments - her sexual activity, Threatened with medical procedure, Negative comments - her appearance, Threatened to withhold care, Negative comments - baby's appearance, Threatened with physical violence, Blamed woman for poor outcome.Stigma or discrimination: Race/ethnicity, Economic circumstances, Age, Marital status, Level of education/literacy, Religion, HIVstatus, Other stigma/discrimination. Informed consent and confidentiality (C-section non-consented, episiotomy non-consented). Vaginal examinations (first vaginal examination, across all vaginal examinations) (e.g. permissions optained, total number),Pain relief (request for pain relief, receive of pain relief). Neglect and abandonment (no staff member present when baby came out), Supportive care (Woman not offered to have a labour companion during labour and birth, Companion not present at any time during labour and birth, Companion not present at the time of birth, Woman did not have easy access to water or oral fluids during labour, Woman not told she could mobilise during labour, and did not mobilise during labour, Woman not asked for her preferred birthing position), Health systems: Woman instructed to clean up blood, urine, faeces, or amniotic fluid, Staff suggested or asked the woman or companion for a bribe, informal payment, or gift, Curtains, partitions, or other measures used to provide privacy for the women throughout labour, childbirth and post-partum period. Questionnaire: Any physical abuse, verbal abuse, or stigma or discrimination (any physical abuse, any verbal abuse, any stigma or discrimination), Failure to meet professional standards (c-section non-consented, episiotomy non-consented, induction of labour, non-consented), vaginal examinations (non-consented), general description of experience of vaginal examinations (comfortable, a little uncomfortable, quite uncomfortable, very unformfortable), pain relief (Woman not offered pain relief during time in hospital,Woman requested pain relief, Woman requested pain relief but did not receive it,Woman denied pain relief during time in hospital); Neglect and abandonment: Staff member not present when the baby came out (2187 women with vaginal birth), Woman waited for long periods of time before attended by health workers, Woman felt ignored, neglected, or that presence was a nuisance for health workers or staff. Communication: Language interpretation needed, Interpreter not available, woman felt that health workers or staff did not listen and respond to her concerns. Supportive care: Not allowed to have a labour companion during labour and birth, Did not have a labour companion present at any point. Autonomy: Did not have easy access to water or oral fluids, not allowed to eat, Woman not told to or did not mobilise during labour, Woman did not have a preferred birthing position, Woman or baby detained in hospital because of inability to pay hospital bills. Health systems: Curtains, partitions, or other privacy measures not used, Staff suggested or asked for a bribe, informal payment, or giftm Woman instructed to clean up own blood, urine, faeces, or amniotic fluid after birth | | | | | | |
| (20) | Childbirth experiences related to obstetric violence in public health units in Quito, Ecuador. | Ecuador, South America | To "explore women’s experiences of obstetric violence related to childbirth in Ecuador". | 388 | Women between 1 hour and 1 month after an uncomplicated low-risk delivery. | „The definition of obstetric violence is, “the acts of dehumanizing treatment, abuse of procedures, and loss of autonomy that affect the quality of life of women.” It is important to realize that obstetric violence is considered another form of gender violence against women (…)”. |
| **Items:** (1)Non-consensual care: including the option of vaginal delivery after cesarean; freedom of movement during childbirth; freedom of movement during labor; and immediate attachment and permanence of skin-to-skin contact with the mother immediately after delivery. (2)Physical violence, including the Kristeller maneuver; routine episiotomy; routine oxytocin; repeated vaginal examinations (1–4 hours recommended); vaginal examinations by several individuals outside the recommended time frame; and shaving. (3) Lack of information, including execution of procedures without information and/or without consent. (4) Psychologic violence, including intimidating language; inappropriate comments; and lack of respect for cultural customs. (5) Negligent care, including lack of breastfeeding help and absence of a professional during delivery. (6) Confidentiality and discrimination, including privacy. (7) Perception of obstetric violence, including patient perception about their experience in relation to obstetric violence via the following questions, “Do you know what obstetric violence is?” and, “Do you think you have experienced obstetric violence?” | | | | | | |
| (21) | Validation of the Spanish version of Mackey childbirth satisfaction rating scale. | Spain, Europe | To "describe the internal structure of the scale and validate the reliability and validity of concept of its Spanish version MCSRS-E". | 463 women | Women aged 18–45 who had just delivered a singleton live baby at 38–42 weeks through vaginal delivery and women who had undergone an unplanned cesarean section. Women who had difficulty speaking and understanding Spanish were excluded. | “The outcomes of health care delivery are measured in terms of effectiveness and efficiency but also in terms of the individual’s experience as a patient. This experience involves pain, autonomy, a feeling of physical and mental well-being and satisfaction with the favorable results achieved (...) and provides a unique opportunity to better understand satisfaction with the quality of the health care provided (…). Satisfaction with health care delivery is significantly associated with patients’ adherence to medical treatment (…), their quality of life) (…) or simply improvements in their health status (…). Therefore, patients’ experiences are increasingly being used inter- nationally as an indicator of the quality and performance of health systems(...), and thousands of surveys are used by health care providers, administrators or policymakers to assess the quality of care, make decisions about pro- visions and organization of health care services, avoid malpractice and support a competitive edge in the health care area (…)”. |
| **Items**: Mackey Childbirth Satisfaction Rating Scale” (MCSRS), six sub- scales: general satisfaction (three items), satisfaction with self (nine items), baby (three items), midwife (nine items), gynecologist (eight items), and partner (two items). (33 items): Q1; Your overall labor experience, Q2: Your overall delivery experience, Q3: Your level of participation in decision-making during labor, Q4: Your level of participating in decision-making during delivery, Q5: Your ability to manage your labor contractions, Q6: Your level of comfort during labor, Q7: Your level of comfort during delivery, Q8: The control you had over your emotions during labor, Q9: The control you had over your emotions during delivery, Q10: the control you had over your actions during labor, Q11: The control you had over your actions during delivery, Q12: Your partner’s help and support during labor, Q13: Your partner’s help and support during delivery, Q14: Your baby’s physical condition at birth, Q15: The amount of time which passed before you first held your baby,Q16: the amount of time which passed before you first fed your baby, Q17: The physical care you received from the nursing staff during labor and delivery, Q18: The physical care you received from the medical staff during labor and delivery, Q19: The technical knowledge, ability, and competence of the nursing staff in labor and delivery, Q20: The technical knowledge, ability, and competence of the medical staff in labor and delivery, Q21: The amount of explanation or information received from the nursing staff in labor and delivery, Q22: The amount of explanation or information received from the medical staff in labor and delivery, Q23: The personal interest and attention given you by the nursing staff in labor and delivery, Q24: The personal interest and attention given you by the medical staff in labor and delivery, Q25:The help and support with breathing and relaxation which you received from the nursing staff in labor and delivery, Q26: The help and support with breathing and relaxation which you received from the medical staff in labor and delivery, Q27: The amount of time the nurses spent with you during labor, Q28: The amount of time the doctors spent with you during labor, Q29:The attitude of the nurses in labor and delivery, Q30: The attitude of the doctors in labor and delivery, Q31:The nursing staff’s sensitivity to your needs during labor and delivery, Q32: The medical staff’s sensitivity to your needs during labor and delivery, Q33: Overall, the care you received during labor and delivery, overall, how satisfied or dissatisfied are you with your childbirth experience? | | | | | | |
| (22) | Obstetric Violence in Mexico: Results From a 2016 National Household Survey | Mexico, North America | To "assess the prevalence and factors associated with experiences of obstetric violence (obstetric abuse and violence, and nonconsensual care) among women between 15 and 49 years of age in their latest childbirth within the last 5 years". | 24064 | Mexican women aged 15-49 who had given birth during the previous 5 years (from October 2011 to 2016). | “Obstetric violence as a specific type of gender violence affecting women, rather than as a problem of poor- quality health care service or mistreatment and abuse in health care services that might affect any patient (…) This perspective, which is adopted in our study, allows mistreatment and abuse to be studied in the broader context of the various types of violence suffered by women. (…) this phenomenon was legally defined in Venezuela as: the appropriation of a woman’s body and reproductive processes by personnel, expressed as dehumanizing treatment, an abuse of medication, and the pathologization of natural processes, bringing about a loss of autonomy and the capacity to freely decide about their bodies and sexuality, negatively impacting the quality of life of women (…) “. |
| **Items:** Physical abuse: (a) Were you forced to stay in an annoying or uncomfortable position? (b) Did they refuse to anesthetize you or apply a pain blocker without providing any explanation? Non-dignified care: (c) Did they yell at you or scold you? (d) Did they say offensive or humiliating things (e.g., “Is that how you screamed when he did that to you?” or “When he did it, you opened your legs all right, didn’t you?”).Abandonment of care: Abandonment of care was captured through three items: (e) Were you ignored when you asked things about your delivery or about your baby? (f) Did they take a long time to assist you, saying that you were screaming or complaining a lot? (g) Were you pre- vented from seeing, holding, or breastfeeding your baby for more than 5 hours for no reason or without being told of a reason for the delay? Non-consented care: (h) Were you sterilized, given a contraceptive, or had an IUD (intrauterine device) inserted, or had surgery to prevent you from having more children without being asked or letting you know? (i) Were you pressured to accept the insertion of an IUD or an operation to prevent further pregnancies? (j) Were you obliged or threatened to sign a piece of paper without being told what it was or what it was for? Two addi- tional questions were asked only to women who had a C-section: (k) Were you informed in such a way for you to understand why a C-section was necessary? (l) Did you give permission or authorization for the C-section? | | | | | | |
| (23) | Women's perception of support and control during childbirth in the Gambia, a quantitative study on dignified facility-based intrapartum care | The Gambia, Africa | To "(1) assess women’s perception of support and control during childbirth (2) to identify related factors influencing per ceptions of support and control during childbirth". | 200 | Women aged 18-35 years, with no medical or obstetric complication during pregnancy; at least 3 h prior to delivery. | "Childbirth is a stressful experience for women worldwide, therefore, it is of paramount importance for care- givers to be supportive and create an atmosphere that allows women to gain autonomy over the process of childbirth to ensure positive dignified birth experience.Supportive care from providers and care that help women to obtain their level of control enhances dignity during childbirth, promote women’s partici- pation in decision-making regarding their care, re- duce obstetric interventions during birth, and promote positive decisions concerning the utilization of maternity health services in future pregnancies." |
| **Items:** 33-item scale with three subscales, namely; internal control, external control, and support. Internal control: 1.The pain was too great for me to gain control over it, 2.I was overcome by the pain , 3.I was able to control my reactions to the pain , 4.I was mentally calm , 5.I was in control of my emotions, 6.I felt my body was on a mission that I could not control , 7.Negative feelings overwhelmed me, 8.I gained control by working with my body, 9.I could control the sounds I was making, 10.I behaved in a way, not myself. External control: 1. I had control over when procedures happened , 2.I could influence which procedures were carried , 3.I decided whether procedures were carried out or not , 4.People in the room took control, 5.I had control over the decisions that were carried out or not, 6.I could get up and move around as much as I wanted, 7.People coming in and out of the room was beyond my control. 8.I chose whether I was given information or not, 9.I could decide when I received information, 10.I had control over what information I was given, 11. I felt I had control over the way my baby was finally born. Support: 1. The staff helped me find the energy to continue when I wanted to give up.2. The staff seemed to know instinctively what I wanted or needed,3. The staff went out of their way to try a new way to try to keep me comfortable., 4. The staff encouraged me to try new ways of coping (such as breathing techniques)., 5. The staff realized the pain I was in, 6. The staff encouraged me not to fight what my body was doing , 7. I felt like the staff had their own agenda, 8. I felt like the staff tried to move things along for their own convenience, 9. I was given time to ask questions, 10. The staff helped me to try different positions , 11. The staff stopped doing something if I asked them to stop , 12. The staff dismissed things I said to them | | | | | | |
| (24) | Comparative study analysing women's childbirth satisfaction and obstetric outcomes across two different models of maternity care | Spain, Europe | To "describe the differences in obstetrical results and women’s childbirth satisfaction across 2 different models of maternity care (biomedical model and humanised birth)". | 406 (204: biomedical model, 202 humanised model) | Mothers aged 16-49 who had given birth at two university hospitals in south-eastern Spain. Women with elective cesearean section and women not understanding spanish or english were excluded. | “Satisfaction with childbirth is the most important qualitative outcome in assessing childbirth experiences, given the fact that this experience affects their health and their relationship with their infant. (…) Five dimensions: the delivery experience (pain intensity, complications and length of labour), medical care, nursing care, information received and participation in the decision-making process, and physical aspects of the labour and delivery rooms. (…) identified the following features of obstetric care as influencing satisfaction with childbirth: explanation of procedures and involvement of mothers in administering or choosing them; support from the presence of a partner and qualified hospital staff; and physical comfort of the postnatal ward. (…) described factors contributing to a satisfying birth experience as follows: support, information, intervention, decision-making, control, pain relief and trial participation (…)”. |
| **Items:** 1) Professional support during labour: All my labour carers were very supportive. Carers always listened very, very carefully to everything that I had to say. During labour, there was always a carer to explain things so that I could understand. All my carers treated me in the most friendly and courteous manner possible. My carers couldn’t have been more helpful. 2) Expectations: My labour went totally normally.The labour went nearly exactly as I had hoped that it would.The delivery went almost completely as I had hoped that it would.My labour was just about the right length. 3) Holding baby: I got to see my baby at exactly the right time after she/he was born.After my baby was born, I was not given him/her quite as soon as I wanted. I needed to hold my baby a little earlier than I did. 4) Support from husband:My birth partner/husband helped me to understand what was going on when I was in labour.My birth partner/husband helped me to understand what was going on when I was in labour. Pain in labour: I should have been offered something more to relieve my labour pains.I got excellent pain relief in labour.More pain relief would have made my labour easier. Pain after delivery: I should have been offered something more to relieve the pains I had after my baby was born.I was in a fair bit of pain immediately after the birth.I didn’t need a lot of pain relief after the birth. Environment: My birth room was a little impersonal and clinical. The area where I gave birth was very pleasant and relaxing. Control:Everyone seemed to tell me what to do in labour. Labour was just a matter of doing what I was told by my carers. General satisfaction: The way my labour care was provided could not have been improved. I am satisfied with just one or two things about the labour care that I received | | | | | | |
| (25) | Labor and birth in the rural region: obstetric violence. | Brazil, South America | To "investigate forms of obstetric violence in childbirth and birth care". | 169 | Women who had delivered in the selected hospital. Recruited at least 12 hours after giving birth. Live births only. | „The term obstetric violence is used to describe the various forms of violence that occur in the care of pregnancy, childbirth, postpartum and abortion. It is understood by the appropriation of the body and the reproductive processes of women by health professionals who express themselves through dehumanizing relations, abuse of medicalization and pathologization of natural processes resulting in loss of autonomy and ability to freely decide on their body and sexuality and negatively impacting the quality of life of women (…)” |
| **Items:** Categories and variables of disrespect and abuse/obstetric violence 1. Physical abuse: Episiotomy, Cesearean, touching during Labor, average of touching, 2. imposition of non-consensual interventions: C-section due to circular cord, C-section due to post maturity, C-section to get a tincture (?) 3. non-confidential or private care: Absence of private room, Absence of a companion (Labor, birth, puerperium), 4.undue care and verbal abuse: not specified, 5. discrimination based on certain attributes: not specified, 6. abandonment, neglect or refusal of assistance: not specified, 7. detention in services: not specified | | | | | | |
| (26) | Discordance in self-report and observation data on mistreatment of women by providers during childbirth in Uttar Pradesh, India. | India, Asia | To "assess provider mistreatment of women during childbirth via self-reports and observations and examine the concordance between these two measures". | 1047 (for observations) 875 (for follow-up) | Married women aged 17-49 who had delivered at the selected hospitals. | „A growing body of literature suggests that fear of such mistreatment is a key impediment to timely acquisition of care and use of institutional facilities for childbirth, particularly among less educated and poor women, and is associated with poor birth outcomes for both mother and child (…) Such mistreatment can include a broad array of provide behaviors, from neglectful or non-consensual care to verbal or physical abuse against a woman during childbirth (…)”. |
| **Items**: 1.Beaten / slapped by health care provider,2.Provider forcefully pushed abdomen during delivery, 3.Provider applied force to pull baby, 4.Provider used bad/abusive language, 5.Provider threatened to slap client, 6.Client faced problem due to unavailability of provider during delivery, 7.Client disrespected during stay at facility, 8.Client not provided complete information on the delivery procedures, 9.Client not provide information on problem you might face after delivery, 10.Provider did not answer client’s questions, 11.Provider did not tell client about her health, 12.Provider did not tell client about her baby’s health, 13.Provider did not advice client on avoiding illness after delivery, 14.Provider did not take client’s consent before conducting the delivery procedures, 15.Client was denied specific things by the provider 16.Client was treated differently based on her caste, 17.Client was discriminated during her stay at facility | | | | | | |
| (27) | The relationship between women's experiences of mistreatment at facilities during childbirth, types of support received and person providing the support in Lucknow, India | India, Asia | To "measure the association between support (who was with a woman at the time of childbirth), what type of support that person provided, and the level of mistreatment a woman experienced". | 392 | Women living in economically disadvantaged (slum) areas, who gave birth within the past 5 years, aged 16–30 at time of survey. | “Mistreatment can encompass a number of factors, including verbal and physical abuse, disrespect, and neglect of various forms (…)”. |
| **Items:** 1. Discrimination based on race, ethnicity, or ability to pay 2. Physical abuse (slapping or hitting) 3. Verbal abuse (insult and shouting), 4. Threatening to withhold treatment, 5. Lack of information about care provided, 6. Ignoring or abandoning patient when in need,7.Delivering alone 8. Denying choice of position during delivery, 9.Birth companion(s) not allowed, 10. Request or suggestion for informal payments or bribes for better care, 11. Unnecessary separation from baby after birth | | | | | | |
| (28) | Women's empowerment and experiences of mistreatment during childbirth in facilities in Lucknow, India: results from a cross-sectional study | India, Asia | To "identify associations between women’s empowerment and experiences of mistreatment during childbirth by examining associations between reports of multiple types of disrespect and empowerment, utilizing the GEM scale". | 392 | Women living in economically disadvantaged (slum) areas, who gave birth within the past 5 years, aged 16–30 at time of survey. | „(…) domains of mistreatment, including physical, sexual, and verbal abuse, stigma and discrimination, failure to meet professional standards of care, poor rapport between women and providers, and health care-related conditions and constraints“. |
| **Items:** 1. Discrimination based on race, ethnicity, or ability to pay 2. Physical abuse (slapping or hitting) 3. Verbal abuse (insult and shouting), 4. Threatening to withhold treatment, 5. Lack of information about care provided, 6. Ignoring or abandoning patient when in need,7.Delivering alone 8. Denying choice of position during delivery, 9. Birth companion(s) not allowed, 10. Request or suggestion for informal payments or bribes for better care, 11. Unnecessary separation from baby after birth, Gender equitable men score (29). | | | | | | |
| (30) | Patient and provider determinants for receipt of three dimensions of respectful maternity care in Kigoma Region, Tanzania-April-July, 2016. | Tanzania, Africa | To "collect and analyse interviews linked between clients and providers, allowing for description of both patient and provider characteristics and their association with receipt of RMC". | 935 women and 249 providers | Providers: Clinicians, nurses/midwifes, other staff, Clients/women: 15-40 years | “Respectful Maternity Care Charter defined seven rights of childbearing women(…): Freedom from harm and ill treatment; Right to information, informed consent and refusal, and respect for choices and preferences, including the right to companionship of choice whenever possible; Confidentiality, privacy; Dignity, respect; Equality, freedom from discrimination, equitable care; Right to timely health care and to the highest attainable level of health; and Liberty, autonomy, self-determination, and freedom from coercion.”. |
| **Items:** Every woman has the right to be free from harm and ill treatment (Article I): 1. Did any of the health facility staff ever physically abuse you during your visit? By physical abuse, we mean, did they hit, slap, push, kick you, or use any other type of physical force against you ( Absence of physical abuse): Every woman has the right to information, informed consent and refusal, and respect for her choices and preferences, including the right to her choice of companionship during maternity care, whenever possible (Article II): 2. Did the staff explain what will happen during your labor and delivery? (Explain what will happen) 3. Did the staff get your consent before proceeding with procedures and exams? ( Consent before procedures/exams) 4. Did the staff explain procedures or exams before proceeding? (Explain procedures/exam beforehand) 5. Did the staff inform you of the findings from procedures and exams? (Inform about findings from procedures/exams) 6. Did you feel the information given to you during your visit was too little, just about right, or too much? (Right amount of information) 7. Did the staff ask if you have questions? (Provider asked if any questions) 8. Did the staff encourage you to have a support person with you throughout labor and delivery? (Provider encouraged companion) 9. Did you feel comfortable to ask questions during the visit? (Client comfortable asking questions)  Did the staff... (Index for receipt of post-delivery counseling). 10. Counsel you about danger signs you should look for in yourself such as too much bleeding, fever, or breast pain? 11. Counsel you about danger signs you should look for in your baby such as refusing to breastfeed, fever, or convulsions (fits)? 12. Tell you what to do if you or the baby have any problems? 13. Counsel you on good body hygiene to prevent infections? 14. Counsel you on breastfeeding? 15. Counsel you about exclusive breastfeeding (not using any other fluid/food except breastmilk)? 16. Ask about your reproductive goals? By reproductive goals, we mean did the provider ask about your desire to have children in the future or to use family planning. 17. Counsel you on when you can have sex with your husband/partner? 18. Counsel you on when you can bear another pregnancy? 19. Counsel you on the risks of sexually transmitted infections (STIs), including HIV? 20. Counsel you on how to prevent sexually transmitted infections (STIs), including HIV? 21. Tell you when to return for a follow-up visit? Every woman has the right to privacy and confidentiality (Article III): 22. Do you believe the information you shared about yourself with the health care provider will be kept confidential? (Client feels provider will keep information confidential) 23. Did the staff provide privacy during counseling or exams such as using a private room, screens, curtains, or cloths to cover you? (Given privacy for exams or counseling) 24. When meeting with the health care provider during the visit, do you think other clients could hear what you said? (Other clients could not hear discussions). Every woman has the right to be treated with dignity and respect (Article IV): 25.Did the staff introduce themselves? (Provider introduced self) 26.Did the staff greet you respectfully? (Greeted respectfully) 27. Did any of the staff ever emotionally abuse you during your visit? By emotional abuse, we mean, did they speak or act in an angry or condescending way that made you feel badly about yourself, degraded, embarrassed, or sad? (Absence of emotional abuse) 28. Did the staff interact in a friendly way? (Interacts in a friendly way) 29. Overall, how would you rate the staff’s kindness in the way they spoke to you during this visit? (Kindness) 30. Overall, how would you rate the staff’s level of encouragement during labor and delivery? (Encouragement) 31. How would you rate the facility’s level of cleanliness? (Level of cleanliness) 32. Did the staff advise you on what you could do to make yourself more comfortable when you were in pain? (Advised of comfort measures) What comfort measures did the staff provide to make you more comfortable? (Index for receipt of comfort measures): 33. Rubbed back 34. Offered fluids to drink 35. Offered food to eat 36. Assisted in changing position 37. Helped to walk around 38. Used encouraging words Every woman has the right to healthcare and to the highest attainable level of health (Articvle VI): 40. Every woman has the right to healthcare and to the highest attainable level of health (Provider came when called) 41. Did the staff pay close attention to you throughout labor and delivery? (Close attention in labor) 42. Did the health care staff visit you regularly during the course of labor? (Visited regularly in labor) 43. Was a health care provider with you at the moment of delivery? (Provider present at delivery) 44. Did you feel that your waiting time (when you first arrived at this facility and the time you saw a staff person for a consultation) was reasonable or too long? (Wait time from arrival to care) 45. How long ago was your new baby born? (proxy measure from birth to time from time of exit interview) (Discharge time 24 h or more after delivery) 46. Did the facility provider supplies for your labor and delivery care? (Facility provided birth supplies) | | | | | | |
| (31) | The relationship between prenatal control, expectations, experienced control, and birth satisfaction among primiparous women | United States of America, North America | To "study how all three issues of control affect one another and how they collectively influence birth satisfaction levels. We seek to address the following questions: 1. What is the relationship between prenatal control, expectations of control, and experienced control? 2. How do prenatal control, expectations of control, and experienced control relate to birth satisfaction?" | 31 | Primiparae women, 26-40 weeks pregnant, aged between 18-35 years, 13 were African-American, 14 Caucasian. | “Birth satisfaction refers to a woman’s satisfaction with her birth experience throughout labour, birth, and the immediate postpartum period (…). It is assessed by measuring the mother’s perceptions of care received, maternal control, personal support, medical interventions, and overall health (…) Birth satisfaction is an important construct, as unsatisfying birth experiences are associated with the occurrence of postpartum depression and even posttraumatic stress disorder (…). Research has consistently identified control as one of these factors that greatly affects a woman’s assessment of the quality of her birth Issues of control during pregnancy and childbirth manifest themselves in three ways. These include prenatal control of fetal health during the pregnancy, expectations of control for labour and birth, and actual control experienced during childbirth (…)”. |
| **Items:** Prenatal control (from: ‘Internal Control of Birth Outcomes before Birth, Weisman et al. 2008), e.g.: There is nothing I can do to make sure my child is born healthy.It is my job as a mother to make sure my child is born healthy.I could make very few choices that would affect my child's health at birth.I could do many things to make sure my child is born healthy. Expectations of control during childbirth (Hauck et al.,2007). This ‘Birth Expectations’ scale was used to assess the kinds of expectations pregnant women have for their childbirth experiences. Experienced control in the delivery room (items not listed) Birth satisfaction: ‘Labor and Delivery Satisfaction Index (LADSI) (Lomas et al., 1987), additional and modified items (not listed). | | | | | | |
| (32) | Disrespect and abuse during facility-based childbirth in southern Mozambique: a cross-sectional study | Mozambique, Africa | To "assess the experience of women giving birth in hospital in different settings in Maputo City and Province, Mozambique". | 520 | All women aged 18–45years who had delivered at the participating hospitals and who spoke Portuguese or Changana, were invited for an interview. | “(…) physical abuse; non-consented care; non-confidential care; non-dignified care; discrimination; abandonment of care; and detention in facilities (…).The mistreatment of women during childbirth often occurs at the level of the interaction between women and healthcare providers but deficiencies in the health care system (e.g. lack of adequate personal and poor infrastructure) also contribute to its occurrence (…)”. |
| **Items:** Services without permission: Caesarean section, Episiotomy, Stitching, Blood transfusion, Sterilization, Injection, Shaving. Lack of confidentiality: disease (HIV),Age, medical history, absence or position of the father During labour and delivery. Disrespectful treatment: Threatened with C-section, Scolded, shouted at slanderous remarks, blamed or intimidated. Physical violence: beaten, slapped or pinched; tied down or restrained; episiotomy sutured without anesthesia; sexually abused by health worker. Discrimination: ethnicity, young and unexperienced, single motherhood, status HIV sero-positive status, low socio-economic status. Detention in facility: unpaid bills mother, unpaid bills baby | | | | | | |
| (33) | Client satisfaction with existing labor and delivery care and associated factors among mothers who gave birth in university of Gondar teaching hospital; Northwest Ethiopia: Institution based cross-sectional study | Ethiopia, Africa | To " estimate the degree of patient satisfaction and its determinants in labor and delivery care in the University of Gondar Hospital". | 593 | All mothers who delivered at University of Gondar Hospital from July through September 2016; exclusion of those those who were critically ill and unable to communicate. | “Studies show that women accessing modern institutional health care still face many challenges including disrespectful, abusive, and inhumane ways of treatment, especially during labor and delivery processes. Such treatment violates the right of women to respectful care, and can also threaten their rights to life, health, bodily integrity, and freedom from dis- crimination (…). Evidence has shown that dissatisfied mothers, especially in the developing world like Ethiopia, tend to prefer utilizing traditional means of health care, using modern health care services as a last resort (…)”. |
| **Items**: Not available | | | | | | |
| (34) | Mothers' experience of disrespect and abuse during maternity care in northern Ethiopia | Ethiopia, Africa | To "assess the extent of, and factors associated with, disrespectful and abusive maternity care reported by women who utilised facilty-based delivery services in northern Ethiopia". | 1125 | Women who gave birth in the past 12 months and visited public health facilities for birthing. | “Disrespectful and abusive care includes impoliteness of care providers, inappropriate reprimands, shouting at the client, lack of empathy, refusal to assist, threatening clients for their non-compliance, and denying clients opportunities to choose or give an opinion on the care they are receiving”. |
| **Items:** Shouted at; scolded/insulted; discouraging/became negative to me; request for assistance/help ignored; not told information before/during a procedure; providers discussed my private health information in public; shared my health information with others; was ade to lay on unhygienic bed/couch; ashamed for being exposed naked to others; movement restricted for a long time; procedure/s done without being adequately informed; left alone unattended; hit, slapped, pushed by provider; anything that you do not want to mention | | | | | | |
| (35) | Satisfaction with Delivery Services Offered under the Free Maternal Healthcare Policy in Kenyan Public Health Facilities | Kenya, Africa | To "determine satisfaction level of mothers with the free maternal services in selected Kenyan public health facilities after the implementation of the free maternal healthcare policy". | 2216 | The respondents were mothers who had delivered in the health facilities, had been discharged from the health facilities, and were waiting go home. | “Patients’ satisfaction with healthcare services is one of the measures for quality of care that has been shown to influence confidence in a health facility and the subsequent utilization of services from the facility (…). Patients’ satisfaction with quality of healthcare is the degree to which the patients’ desired expectations, goals, and preferences are provided by the healthcare service providers (…). Patients’ satisfaction and dissatisfaction with healthcare services indicate their perception about the strengths and weaknesses in the service delivery (…)”. |
| **Items**: Satisfaction with: 1. Consultation Time, 2. Communication by the health care workers, 3. Availability of staff in the delivery rooms, 4. Availability of Staff in the wards, 5. Cleanliness in the health facilities, 6. Privacy in the wards, 7. Availability of drugs and supplies | | | | | | |
| (36) | Construct and content validity of the Turkish Birth Satisfaction Scale – Revised (T-BSS-R) | Turkey, Asia, Europe | To "assess factor structure, validity, and reliability of the Turkish Birth Satisfaction Scale – Revised (T-BSS-R) using data collected from a Turkish population". | 120 | Turkish-speaking, low-risk women who had experiences vaginal delivery of a full termed baby. | “Birth satisfaction: "tells how a woman feels about her birth experience, which requires the midwife to take into consideration her personal wants and needs within confines of safety and cost (…). Markers of ‘birth satisfaction’ include, for example (…): considering person-centred preparation for childbirth, providing respect and support throughout the birth process, maintaining open and honest communication, affording a comfortable environment in which the woman is less likely to lose control, offering acceptable methods of pain relief, minimising obstetric injury, and helping the woman to give birth in her desired position (…). Levels of ‘birth satisfaction’ can affect the mental health of both mother and infant, with a negative experience having the potential to reduce mother–infant attachment, reduce willingness to breast-feed, instigate sexual dysfunction, instigate infant neglect/abuse, result in postnatal depression (PND), post-traumatic stress disorder (PTSD) and request for future elective cesarean section (CS), and lead to requests for sterilisation and/or abortion (…)”. |
| **Items:** 10-item Birth Satisfaction Scale – Revised (BSS-R): 1. I came through childbirth virtually unscathed. 2. thought my labour was excessively long.3. the delivery room staff encouraged me to make decisions about how i wanted my birth to progress.4. I felt very anxious during my labour and birth.5. I felt well supported by staff during my labour and birth.6. The staff communicated well with me during labour.7. I found giving birth a distressing experience.8. I felt out of control during my birth experience.9. I was not distressed at all during labour.10. I the delivery room was clean and hygienic. | | | | | | |
| (37) | Development and psychometric testing of the scales for measuring maternal satisfaction in normal and caesarean birth | Turkey, Asia, Europe | To "develop a scale to measure maternal satisfaction with birth to evaluate women's experience in labour and the early postpartum period". | 500 (250 vaginal births, 250 caesarean sections) | Low risk post-partum women who gave birth to a single healthy fetus over 37 weeks of gestation during the study period. | “Women’s satisfaction with their childbirth experience also has implications for the health and well-being of a woman and her newborn. A woman’s satisfaction with her childbirth experience may have immediate and long-term effects on her health and her relationship with her infant, including: postpartum depression, post-traumatic stress disorder, future abortions, a lack of ability to resume sexual intercourse, preference for a caesarean section, negative feelings towards her infant, poor adaptation to the mothering role and breast-feeding problems (…)”. |
| **Items:** Perception of health professionals: doctors, midwives and nurses treated me/behaved well. Nursing care in labour: midwives and nurses spent enough time helping me. Cope with pain. Preparation for caesarean: midwifes and nurses spent enought time helping me to prepare for caesarean birth. Comforting: I'd like to have had more help to reduce my stress during childbirth. Information and involvement in decision-making: I was informed about all necessary procedures Meeting baby: I would have like to breastfeed my baby earlier. Postpartum care: nurses met my needs adaquately. Hospital room:room was comfortable and clean.Hospital facilities: we could easily find everything we needed. Respect for privacy: healthcare personnel showed respect ot my privacy. Meeting expectations: I could not get any better care int his hospital | | | | | | |
| (38) | The role of women´s attitudial profiles in satisfaction with the quality of their antenatal and intrapartum care. | Sweden, Europe Australia, Australia | To "compare perceptions of antenatal and intrapartum care in women categorized into three profiles based on attitudes and fear". | 505 | Pregnant women from one hospital in Sweden (n = 386) and one in Australia (n = 123).Women had normal ultrasounds during pregnancy and could speak, write and understand swedisch or english | “Satisfaction, patient perceptions, and actual experiences of the care received are not synonymous concepts, although the terms are often used inter- changeably within one study and between studies (…) Most women report high satisfaction with maternity care, but when asked to consider particular aspects of that care they are more critical. During the intrapartum period, women are consistently dissatisfied with three dimensions of care: their perceived sense of control (…), support received from caregivers (…), and their experiences of managing pain (…). Dissatisfaction has been reported to be associated with operative delivery (especially emergency caesarean) and admission of the infant to neonatal intensive care (…)”. |
| **Items**: Fear of birth scale (FOBS): Deficiencies in the content of antenatal care: 1. Information about pregnancy, 2.Information about breast-feeding, 3.Information about labour and delivery,4. Information about the period following birth, 5. Medical care, 6. Emotional/psychological care,7. The midwife/doctor gives me all the support I need, 8.The midwife/doctor makes my partner feel involved, 9.The midwife/doctor shows engagement ‘cares about me’, 10.The midwife/doctor seems to understand my situation,11. Opportunities to talk about my health,12. Opportunities to discuss ailments & taken seriously, 13.Emotional care received during pregnancy, 14.Medical care received during pregnancy, 15. Overall care received during pregnancy, Deficiencies in the content of intrapartum care:1. Midwife gave me all the support I needed,2.Support from my partner during labor and birth, 3. I was involved in decision making during labor and birth, 4. I was in control of my body during labor and birth, 5. Midwife present in room as much as I wanted during labor & birth, 6. I received information about the progress of labor, 7. I got the best possible medical care during labor and birth, 8. I got the pain relief I wanted during labor and birth, 9. The midwife I met most of the time involved my partner in the care, 10. Opportunity to discuss birth afterwards with the assisting midwife, 11. Best possible help when I was breastfeeding the first time, 12. Satisfaction with emotional care during labor and birth, 13. Satisfaction with medical care during labor and birth, 14. Overall assessment of the care during labor and birth | | | | | | |
| (39) | Women's experiences of mistreatment during childbirth: A comparative view of home- and facility-based births in Pakistan | Pakistan, Asia | To "estimate the prevalence of mistreatment and types of mistreatment among women giving birth in facility- and home-based settings in Pakistan". | 1334 | Women who gave birth in the past 12 months in one of the 14 districts included in the study. | „This mistreatment can have immediate and long-term consequences: for example, denial of pain relief medication, episiotomy (without anaesthesia) and physical abuse can cause extreme pain and suffering (…) It may also lead to adverse psychological effects such as re-traumatisation (…) post-traumatic stress symptoms, sleeping problems, poor self-rated health (…) and feelings of dehumanisation (…) that could result in a distorted body perception and fear of childbirth (…).These categories were reworked as follows: physical abuse, verbal abuse, right to information, non-consented care, non-confidential care, discrimination and abandonment of care.” |
| **Items:** Physical abuse: (a) beating, (b) slapping, (c) push badly to change position, (d) pinch irritably. Verbal abuse: (a) pass insulting or degrading com- ments, (b) harsh tone or shouting, (c) abusive language, (d) threatening for poor outcomes; Non-consented care: (a) perform procedure without consent, (b) explain about the proce- dure to be used for delivery, (c) offer choices regarding births, (d) coercion to undergo caesar- ean section. Right to information: Aa) share results/diagnosis of medical reports, (b) encourage to ask questions, (c) regularly share progress of childbirth; Non-confidential care:(a) privacy during examination, (b) cover woman while taking to and from labour room (c) assure woman for confidentiality of information, (d) women-provider conversation over- heard by others (stranger, other patients, or non-medical staff). Abandonment of care: (a) abandon women during childbirth or afterward, (b) ignore while asking pain relief/medica- tion, (c) delay birthing after deciding for operative procedure; and Discrimination: (a) denial of service due to ethnicity, (b) denial of service due to lack of money. | | | | | | |
| (40) | Women's perceptions of communication in pregnancy and childbirth: Influences on participation and satisfaction with care. | Australia, Australia | To "investigate pregnant women’s perceptions of their maternity care". | 3531 | Women who gave birth within a two month period (age range: 16-51). | The model encompasses the processes and factors contributing to patient participation and its outcomes. Street (2001) suggested that verbal participation (the extent to which patients ask questions, make assertive utterances, express concerns, and provide narratives of their health experiences) influences the quality of care (e.g., satisfaction with the encounter, quality of information patients receive), which is, in turn, related to health outcomes (e.g., adherence to treatment). 3 factors direct a patient’s level of involvement in a medical encounter: predisposing factors, enabling factors, and provider responses: Predisposing factors (a person’s cultural and social background, including status, age, education, and their variant combinations) influence how a person communicates with a health provider.Enabling factors (knowledge of the topic, experience with the topic, interpersonal skills) also affect levels of communication participation.Communication by the health care provider is the final factor that influences the ways and extent to which patients participate. Patient participation affects the perceived and actual quality of care in the immediate and longer term. |
| **Items:** Client-centrered communication, Communication about choices, Participation in decision-making, Perceived quality of care received: | | | | | | |
| (41) | Development and psychometric properties of the Birth Satisfaction Scale-Revised (BSS-R) | United Kingdom, Europe | To "assess factor structure, validity and reliability of the Birth Satisfaction Scale (BSS) and to develop a short-form version of the tool." | 228 | Women who gave birth within the last 10 days, were between 16 and 50 year old, without medical diagnosis, poor obstetric history and who delivered a term infant (no preterm or postterm) | “Every woman's perceptions of birth are important, which within this study is conceptualised as ‘birth satisfaction’. In terms of quantitative research, a woman's satisfaction with intrapartum care can only be considered high quality when gratification over what she received is measured as high (…).” |
| **Items:** Quality of care provision: (1a) Home assessment, (1b) Birth environment, (1c) Sufficient support, (1d) Relationships with health care professionalswomen's personal attributes: (2a) Ability to cope during labour, (2b) Feeling in control, (2c) Preparation for childbirth, (2d) Relationship with baby.Stress experienced during labour: (3a) Distress experienced during labour, (3b) Obstetric injuries, (3c) Perception of having received sufficient medical care, (3d) Receipt of an obstetric intervention, (3e) Pain experienced, (3f) Long labour, (3e) Health of baby. (1) I coped well during my birth.(2) The delivery room staff encouraged me to make decisions about how I wanted my birth to progress.(3) I was well prepared for my labour, i.e., read a lot of literature and/or attended parenthood education classes.(4) I found giving birth a distressing experience.(5) I came through childbirth virtually unscathed.(6) I gave birth to a healthy normal baby.(7) During labour I received outstanding medical care. (8) I received a lot of medical intervention, i.e., induction, forceps, section etc. (9) I had a swift and speedy labour. (10) I felt well supported by my partner during labour and birth. (11) I was encouraged to hold my baby for a substantial amount of time after birth. (12) My birth experience was considerably different to what I intended. (13) I had the same midwife throughout the entire process of labour and delivery. (14) I felt that the delivery room was unthreatening and comfortable. (15) I felt very anxious during my labour and birth. (16) I felt out of control during my birth experience. (17) I felt it was better not to know in advance about the processes of giving birth. (18) I was not distressed at all during labour. (19) I felt mutilated by my birth experience. (20) My baby was avoidably hurt during birth. (21) The staff provided me with insufficient medical care during my birth. (22) I had a natural labour, i.e., minimal medical intervention. (23) I thought my labour was excessively long. (24) I felt well supported by staff during my labour and birth. (25) I was separated from my baby for a considerable period of time after my birth. (26) My birth proceeded as I planned it. (27) The staff communicated well with me during labour. (28) The delivery room was clean and hygienic. (29) Giving birth was incredibly painful. (30) Labour was not as painful as I imagined. | | | | | | |
| (42) | The relationship between women-centred care and women’s birth experiences: A comparison between birth centres, clinics, and hospitals in Japan | Japan, Asia | To "ask what are the perceptions and comparison of women-centered care at Japanese birth centres, clinics, and hospitals and what are the relationships between women-centered care and three dimensions of women’s birth experience: 1) satisfaction with care they received during pregnancy and birth, 2) sense of control during labour and birth, and 3) attachment to their new born babies". | 482 | participants were women who had a singleton birth and were admitted to one of the study settings. Women who were seriously ill were excluded | “From the evidence, care provided at birth centres can be called women-centred care (WCC).The four elements of WCC were respect, safety, holism, and partnership and its goal is the general well-being of women, potentially leading to the woman’s empowerment (…). (…) basic attitudes to be important in providing WCC: (1) treating women with respect, (2) providing care in a non-threatening manner, (3) working in collaboration as equal partners, and (4) giving priority to the woman’s preferences over that of the health-care provider (…)”. |
| **Items**: Labour agentry scale, maternal attachment questionnaire, care satisfaction tooll, WCC (questionnaire) (subscale scores, items not available): being respected, feelings of encouragement, effecitive interaction, help in decision-making, noneffective communication, help in decision-making, non-threatening manner, trusting the caregiver, Labour agency scale: feeling of reassurence, no feelings of powerlessness, no feelings of fumbling around, feelings of accomplishment | | | | | | |
| (43) | Risk factors associated with post-traumatic stress symptoms following childbirth in Turkey | Turkey, Asia, Europe | To investigate "factors associated with symptoms of post-traumatic stress (PTS) following childbirth in women with normal, low-risk pregnancies". | 242 | Women with normal, low-risk pregnancies in Nigde, Turkey. | Childbirth is fundamentally a physiological process, but a woman's thoughts and feelings may directly affect the labour and birth. There is some evidence that self-efficacy for labour and birth is associated with less anxiety about birth and greater perception of control during birth. Support from healthcare professionals was as, or more, important than the events of birth, particularly for women's perception of control during birth. A small proportion of women may perceive birth as traumatic and develop post-traumatic stress disorder (PTSD) as a result. Others may experience severe symptoms of post-traumatic stress (PTS) that are distressing but do not reach threshold for a diagnosis of PTSD. . A difficult or complicated birth can lead to the development of PTS if a woman believes her life or her infant's life is in danger during birth and she feels intense fear, helplessness and horror . Symptoms of PTS include intrusive thoughts, flashbacks and nightmares, emotional numbing, avoid- ance of reminders of the birth, and hyper-arousal such as irrit- ability. Loss of control, feeling trapped, and vivid memories of the event have also been noted as experiences and perceptions of women after a difficult or traumatic childbirth. |
| **Items:** Support and control in birth (SCIB) Scale measuring 33 items and three subscales of: internal control (10 items), external control (6 items) and support from healthcare professionals (17 items).Postpartum Self-Evaluation Questionnaire (PPSEQ). The postpartum PPSEQ was developed by Lederman et al. (1981) to evaluate women's adaptation to being a new mother.Fear of childbirth (WDEQ-B). Social support during pregnancy and after birth. Multidimensional Scale of Perceived Social Support (MSPSS; Zimet et al., 1988). The MSPSS is a 12-item scale designed to assess perceived social support. | | | | | | |
| (44) | Ethical aspects of obstetric care: Expectations and experiences of patients in South East Nigeria | Nigeria, Africa | To "describe patients’ expectations and experiences of the ethical conduct of their physicians during obstetric care in three university teaching hospitals in South East Nigeria". | 1112 | Parturient women who had antenatal care and/or delivery at the study hospitals during the study period | "The ethical principle of patient autonomy determines the appropriate attitude of doctors to foster patients’ right to information, primacy in decision making, and confidentiality and privacy. The basic quality of primacy in making decisions is the basis for the requirement of informed consent. (...)Justice with respect to obstetric care is related to fair choices regarding investigations and treatment, especially with respect to cost. It can also be related to fairness in attending to patients in clinics, such as seeing patients on a 'first come, first seen' basis". |
| **Items**: General perception of doctors' conduct. i. Was the conduct of doctors an important consideration in your choice of where to receive maternity care? (yes/no) ii. How do you rate the importance of respectful and dignified handling by doctors during your care in this pregnancy and delivery? Tick only one as appropriate: (a) very important, (b) moderately important, (c) important, (d) unimportant, (e) very unimportant iii. Do you consider free communication between women and doctors during maternity as an important aspect of your care during this pregnancy and delivery? Tick Yes or No. Expectations of ethical conduct (Yes/no): a. Asking for and respecting the opinion of the patient in every decision regarding her investigation and treatment b. Consideration and respect for the religious beliefs of the patient c. Consideration of the ability of the patient to pay in choosing investigations and treatment, d. Strictly ensuring that no physical, emotional pr psychological harm is done to the patient in the course of her care, e. Ensuring that every aspect of her care is meant to do good to the patient, f. Having romantic relationship with the patient, g. Soliciting money or donations from the patient, h. Your personal contribution to decisions on your investigations and treatments, i. Being attended to on “first come, first seen” basis, j. Being seen only when there is a female chaperone, k. Respect for your emotional feelings in the course of your care, l. Respect for your privacy including the confidentiality of your case records 2. Which of these have you experienced in the course of your care in this pregnancy? (Yes/No) a. Being attended to in the presence of too many people without respect for your privacy, b. Being examined alone by a doctor without the presence of a chaperone, c. Having investigation and treatment without asking for your opinion about them, d. Having investigation or treatment without having them explained to you, e. Not being talked to regarding what the doctor found out on your assessment each day of your visit, f. Demand for romantic or sexual relationship from your doctor, g. Demand for monetary reward from your doctor before being treated or after treatment, h. Being touched inappropriately by your doctor, i. Verbal abuse from your doctor. Informed consent for cesarean section: Questions about the detailed explanation of indication, short and long term maternal risks, and explanation of the surgery itself, the process of recovery and costs of surgery, Rating of ethical practices of doctors: Privacy, physical examination in the presence of a chaperone, asking the women's opinion, explaining interventions/ treatments/ findings/ diagnosis/ benefits/ limitations/ risks, counseling, informed consent, demanding payment | | | | | | |
| (45) | Satisfaction with childbirth services provided in public health facilities: results from a cross- sectional survey among postnatal women in Chhattisgarh, India." | India, Asia | To " measure postnatal Indian women’s satisfaction with childbirth services at selected public health facilities". | 1004 | Women who had uncomplicated vaginal births (according to WHO criteria) and women who had a cesarean section, where the mother and the neonate experienced an uneventful postnatal period | “Studies show that women who are satisfied with child- birth services tend to have better self-esteem and confidence, are faster in establishing a maternal–neonatal bond, and are more likely to breastfeed compared with women who are dissatisfied (…). Women who are dis- satisfied with their childbirth experiences are more prone to develop a fear of childbirth and postnatal depressive symptoms, and to face difficulties in breastfeeding and in performing baby and self-care (…)”. |
| **Items:** Basic facilities: own bed, bedsheets, pillows, personal lockers, interaction frequency with providers, Satisfaction with childbirth: Wijma Delivery Experience Questionnaire Version B (WDEQ-B), Edinburgh Postnatal Depression Scale (EPDS), Scale for Measuring Maternal Satisfaction (SMMS): Normal (Vaginal) and Caesarean Births: Facilities and services, Information and involvement in decision making, Maintenance of privacy, Compassion and respect, Managing stress, Intrapartum care received, Meeting the baby, Postpartum care received, Overall support provided Expectations from institutional birth | | | | | | |
| (46) | Intrapartum care could be improved according to Swedish fathers: Mode of birth matters for satisfaction | Sweden, Europe | To "explore Swedish fathers’ intrapartum care quality experiences, with a specific focus on care deficiencies in relation to birth mode. A secondary aim was to explore which issues of quality that contributed most to dissatisfaction with the overall assessment of the care". | 827 | Fathers capable of mastering the swedisch language and non-malformed foetus found at ulktrasound screening (17-19 weeks of pregnancy)., 47% first-timeand 53% repeat fathers | “(…) Parents’ satisfaction with given care has been described as an indicator of care quality,14 and may be used to improve healthcare. Dissatisfaction with given care has been related to professionals lacking skills, giving inadequate information, and professionals restricted in number. To increase satisfaction with given care, the care should be individualized. The most important determinants for patient satisfaction, in general, have been related to respect for patient preferences, and giving emotional and physical support. (…)”. |
| **Items:** Quality of intrapartum care from the fathers’perspective in relation to birthmode: 1. Midwife gave me all the best support I needed, 2. Midwife showed engagement, ‘cared about me’, 3. midwife involved me in care, 4. midwife was present in the room as much as I wanted, 5. I received information about the process of labour, 6. I was involved in decision-making during labour and birth,7. Partner got the best possible medical care, 8.Partner got the best possible help when breast-feeding the first time, 9. Opportunity to discuss the birth afterward with the assisting midwife. | | | | | | |
| (47) | Women’s satisfaction and perception of control in childbirth in three Arab countries. | Egypt, Africa, Lebanon, Syria, Asia | To describe "levels of satisfaction with the childbirth experience and perceptions of control of women giving birth in public hospitals in three middle-income Arab countries, Egypt, Lebanon and Syria, and to determine the service delivery factors associated with their satisfaction". | 2620 (709 in Mansoura, 705 in Beirut and 1206 in Damascus) | Women older than 18 years, who were not classified as high-risk by health care providers and did not suffer from intrauterine foetal death attending one of the three hospitals | “A woman’s satisfaction with the birth experience has been shown to influence her relationship with her infant, to affect her self-esteem and self-image, and influence her future childbirth expectations. Perceptions of being in control during childbirth have been recognised as the strongest component of women’s birth experiences, of their own behaviour during labour and their inter- action with care providers, contributing largely to women’s feelings of fulfilment and postpartum well-being. Satisfaction is also related to the caregiver’s attitude, good communication with care providers, and the responsiveness of staff to women’s needs. One report indicates that dissatisfaction with care and perceptions of diminished control over the process of childbirth have led to a preference for caesarean sections for future births”. |
| **Items:** Satisfaction with childbirth: measured with the Mackey Childbirth Satisfaction Rating scale capturing aspects related to self, partner, baby, nurse/midwife, physician and general rating in six sub-dimensions.Women's perception of control: measured with a shortened version of the Labor Agentry Scale (LAS), 10-item version on a 7-point Likert scale (also translated to Arabic and back-translated into English). Assesses women's agreegment to statements about personal control during childbirth. | | | | | | |
| (48) | Disrespectful and abusive treatment during facility delivery in Tanzania: a facility and community survey. | Tanzania, Africa | To "assess the frequency of disrespect and abusive experiences as reported by women during facility childbirth in eight health facilities in Tanzania. We compared two approaches for measuring prevalence—exit interviews and community follow-up surveys and examined individual and delivery-related factors associated with reports of abusive treatment". | 1779 (exit interviews, of these 593 were interviewed for follow-up) | Women aged 15 years and older and residing in Korogwe and Muheza Districts, rural areas in Tanzania that delivered in one of the 8 selected health facilities | "These include physical abuse (beating, slapping and pinching), lack of consent for care (e.g. for Caesarean section or tubal ligation), non- confidential care (e.g. lack of physical privacy or sharing of confidential information), undignified care (e.g. shouting, scolding and demeaning comments), abandonment (e.g. being left alone during delivery), discrimination on the basis of ethnicity, age, or wealth, or detention in facilities for failure to pay user fees" following the Browser and Hill categorization (…)”. |
| **Items**: Non-confidential care: lack of physical privacy. Non-dignified care: shouting/scolding, threat of withholding treatment, threatening or negative comments.Neglect: ignored when needed help, delivery without attendant. Non-consented care: non-consent for tubal ligation/c-section/hysterectomy. Physical abuse: physical abuse (slapping, pinching, etc.), sexual harassment, rape. Inappropriate demands for payment: detention in facility for failure to pay, request for bribe | | | | | | |
| (49) | Association between disrespect and abuse during childbirth and women’s confidence in health facilities in Tanzania. | Tanzania, Africa | To "examine how experiencing disrespectful and abusive treatment during childbirth influences women’s satisfaction with delivery, perceived quality of care ratings for delivery, and the intention to deliver their next child at the same facility". | 1388 | Women aged 15 years and older and residing in Korogwe and Muheza Districts, rural areas in Tanzania that delivered in one of the two district hospitals | “Disrespectful and abusive treatment during childbirth, such as physical abuse, abandonment, threatening and negative language, shouting and scolding, physical privacy violations, and non-consented care, has been observed in several resource-constrained contexts, including Tanzania (…)”. |
| **Items:** Non-confidential care: lack of physical privacy. Non-dignified care: shouting/scolding, threat of withholding treatment, threatening or negative comments.Neglect: ignored when needed help, delivery without attendant. Non-consented care: non-consent for tubal ligation/c-section/hysterectomy. Physical abuse: physical abuse (slapping, pinching, etc.), sexual harassment, rape. Inappropriate demands for payment: detention in facility for failure to pay, request for bribe | | | | | | |
| (50) | Factors determining satisfaction among facility-based maternity clients in Nepal | Nepal, Asia | To "identify the determinants of client satisfaction with maternity care in Nepal using data from a nationally representative health facility survey". | 477 | Women who delivered at the facility or sought care for intra-partum complications. | “Satisfaction with maternity care is a multidimensional construct embracing satisfaction with self (personal control), and with the physical environment of delivery room and quality of care. Aspects of care that may influence client satisfaction include provider attitude, provider competence, outcome, physical environment, continuity of care, access, information, cost, bureaucracy and attention to psychosocial problems. Quality of care may not al- ways be linearly associated with the level of satisfaction as perceived by the clients; however client satisfaction an important determinant of utilization of health services and the choice of health facility (…). Women who are treated with respect, courtesy and dignity, and have trusting relationships with their care providers are more likely to be satisfied (…). Lack of involvement in decision making and inadequate information about their care are associated with dissatisfaction (…)”. |
| **Items:** Client satisfaction: Accessability: How satisfied were you about the waiting time? Interpersonal communication: How satisfied are you with the information you received from the providers?How satisfied are you with the politeness of the staff with whom you consulted? Physical environment: How satisfied are you with the level of privacy you received? How satisfied are you with the cleanliness of the facility? Clinical care: How satisfied are you with the level of skill the provider had to deliver your baby? How satisfied are you with the care you received at this facility? Decision-making: How satisfied are you regarding your involvement in decision making during the care at the facility? | | | | | | |
| (51) | Components of obstetric violence in health facilities in Quito, Ecuador: A descriptive study on information, accompaniment, and position during childbirth | Ecuador, South America | To "describe the level of obstetric violence in public health facilities in Quito, for the following three components: (1) the information provided to the mother; (2) the accompaniment of the mother; and (3) the positions during childbirth". | 388 | Woman were selected for the study only if they were in the post- partum period, 1 hour to 1 month after a low-risk delivery, and if their child's birth took place in one of the health units included in the study. | „Obstetric violence, a specific type of violation of women's rights, includes the right to equality, freedom, information, integrity, health, and reproductive autonomy (…). In Ecuador, the latest definition of obstetric violence has been extended to include the concept of ‘gynecological-obstetric violence’. It includes: abuse; imposing cultural practices and non- consented scientific procedures; violation of professional secrecy; improper medicalization; inconsideration for natural processes of pregnancy, childbirth, and postpartum; forced sterilization; loss of autonomy and women's incapacity to freely decide over their body and their sexuality; all of which can have a negative impact on women's quality of life, especially in regards to their sexual and reproductive health”. |
| **Items**: 1) non-consensual care, 2) physical violence, 3) information: Informed about the performed procedures, 4) psychological violence , 5) negligent care, 6) confidentiality and discrimination, 7) perception of obstetric violence | | | | | | |
| (52) | Obstetric violence and its associated factors among postnatal women in a Specialized Comprehensive Hospital, Amhara Region, Northwest Ethiopia | Ethiopia, Africa | To assess "the prevalence and associated factors of OV among immediate postnatal women in care in Gondar University Comprehensive Specialized Hospital (GUCSH), Northwest Ethiopia, 2019". | 409 | immediate postnatal women | “Obstetric violence (OV) is a specific type of violation of women’s rights in medical practice during health care related to the childbirth processes. Laboring mothers may be subjected to different forms of OV during facility child birth.. Such ill-treatments and abuses create a psychological distance between the women and care providers and then drive women away from formal health care systems in fear of being subjected to such violence and sometimes are a more prominent hindrance than geographical or financial barriers to maternal health service utilization”. |
| **Items**: Physical abuse: The presence of at least one of the following activities by the care provider on the client: beating, threatening with beating, slapping, pinching, restraining or tying down during labor, cutting or suturing of episiotomy cuts or perineal tears without the use of anesthesia and the use of fundal pressure to fasten the delivery of the baby. Non-consented care: The presence of at least one of the following: providers not giving women or her relatives proper information about medical procedures, not asking for women’s permission to conduct medical procedures such as cesarean sections, episiotomies, hysterectomies, blood transfusions, tubal ligation, augmentation of labor; and coercing into a medical procedures such as a cesarean section. Non-confidential care: The presence of at least one of the following: giving birth in a public view without privacy barriers such as curtains; and having healthcare providers share sensitive clients’ information, such as HIV status, age, marital status, and medical history, in a way that other people who are not involved in their care can hear. Non-dignified care: A report by the client about at least one of the following: intentional humiliation, blaming, rough treatment, scolding, shouting at, women not allowed to bring companion to the labor ward, and ordering to stop crying while they are in labor pain.Discrimination: Discrimination based on specific client attributes like race, age, HIV/AIDS status, traditional beliefs and preferences, economic status, or educational background. Neglected care: If there was any of the following practices: leaving laboring woman alone, women giving birth by themselves at health facilities, failure of care givers to monitor women in labor and intervene in life threatening conditions. Detention in facilities: detaining of mothers in health facility because of bills or damage to the property of the health care facility. | | | | | | |
| (53) | Quality of delivery care in Assiut University Hospital, Egypt: mothers' satisfaction. | Egypt, Africa | To "assess women’s satisfaction with quality of healthcare during hospitalized delivery and to determine factors associated with their satisfaction". | 435 | Women who gave live birth to a singleton the public section of the hospital. | "An important predictor of satisfaction is quality of care (contributors to poor quality of care (…): provider incompetency, lack of drugs and supplies, delay in referral, non cleanliness, and poor interaction between clients and healthcare providers (…). Women are more vocal about patient–provider communication and value good interaction with their provider (…). Mothers who are treated with respect, courtesy, and dignity are more likely to be satisfied with the obstetric care (…). The influences of the attitudes and behaviors of the caregivers are more powerful and obvious on subsequent satisfaction than the influences of pain relief, and intrapartum medical interventions (…) even with the evidence that the majority of women would want pain relief in labor (…). Moreover, it was concluded that poor sanitary condition of the health facilities and lack of basic amenities were the major cause of dissatisfaction (…)”. |
| **Items:** Mothers’ overall satisfaction with delivery care in the hospital was indirectly measured by asking the mother ‘Thinking about your experience, are you going to recommend this facility for delivery to your family (relatives) or friends?’ and ‘Thinking about your experience, if you were to have another baby, would you like to deliver in this hospital again?’Satisfaction with different aspects of quality of care were measured by 14 items: Accessibility: Access to hospital from residence, Interpersonal aspect of care: privacy maintained during care, encouragement at delivery, they way the doctor treated them, the way the nurses treated them, the way the workers treated them, Technical aspects of care: availability of medical facilities, competency of care provider, health advices, Physical environment: cleanliness, availability of beds, sanitary facilities, Outcome of care: health condition of mother, health condition of newborn´ | | | | | | |
| (54) | Disrespect and abuse during childbirth in fourteen hospitals in nine cities of Peru | Peru, South America | To "assess the prevalence of disrespect and abuse during childbirth and ist associated factors in Peru". | 1528 | Women who were under observation in the hospitals after delivering a live neonate within the past 48 hours. | “Disrespect and abuse during childbirth care is considered a form of violence that directly violates women’s rights as defined by the United Nations—i.e., the right to respect, timely care, autonomy, self-determination, and information during childbirth (…)”. |
| **Items**: Non-dignified care: 1. The health personnel did not explain to her what is being done, what to expect throughout labour and birth or did not give the periodic updates on status and progress of her labour. 2. The health personnel who attended her most of the time did not introduce himself/herself to her. 3.The health personnel refused to give her food or fluids when she asked for them.4.The health personnel mocked. laughed about her person or behaviour, or insulted her. 5.The health personnel made her feel guilty for getting pregnant (eg "You're to blame for all this”, “you should have used condoms"). Non-consented care (health personnel did not obtain her consent or permission prior to...): 1.Vaginal examinations, 2. Being examined by other health personnel or a student, 3. Labour induction, 4. Pubic hair shaving, 5. Episiotomy, 6. Caesarean section, 7.Blood transfusions, 8. Tubal ligation, if she had a caesarean section, 9. Hysterectomy, if she had a caesarean section. Non-confidential care: 1. The health personnel did not use curtains or other visual barriers to protect her, 2.The health personnel commented that she was a teenager publicly, 3.Too many people at the delivery room who made her feel uncomfortable, 4. The health personnel commented that she was a single mother publicly, 5.The health personnel commented that she had a sexually transmitted disease publicly. Abondonment of care: 1.The health personnel did not accept when she requested for having a companion during the delivery, 2.The health personnel left her alone or unattended, 3.The health personnel did not attend her, so she gave birth alone. Discrimination based on patients attributes (women felt that health personnel discriminated her…), 1. For her socioeconomic status, 2.For being teenager, 3.For being single, 4. For her religion, 5.For having a sexually transmitted disease. Detention in facilities: 1. The health personnel told her that if she did not cancel for childbirth services she or her baby would stay at the health facility until the payments were made. Physical abuse: 1.The health personnel did not allow her to move around during labour, even if the amniotic sac was not broken, 2.The health personnel performed the Kristeller manoeuvre, 3.The health personnel denied administration of pain medication when she asked for it, 4.The health personnel did not allow her to assume position of choice during birth, 5.The health personnel stitched the episiotomy without anaesthesia, 6.The health personnel tied her up sometime during labour, 7. The health personnel assaulted her physically at some point. Example: Pinching /Slapping/Pushing/Beating Stitching, 8. The health personnel rape her or inappropriate touched her during exam- genital/ thighs | | | | | | |
| (55) | Respectful and evidence-based birth care in Mexico (or lack thereof): An observational study | Mexico, North America | To "describe birth practices and factors associated with respectful and evidence-based care at 15 referral hospitals in Mexico". | 401 | Women giving birth between 2010-2016 | “The law defines obstetric violence as ‘any action or omission of action by health personnel that damages, injures, denigrates or causes the death of a woman during pregnancy, birth and the puerperal period’ (…). More specifically, the law penalises medical negligence, which is expressed as ‘(1) dehumanised care; (2) abuse of medication and pathologisation of natural processes; (3) use of a caesarean section even when the conditions for a natural birth exist; (4) use of contraceptive methods or sterilisation without voluntary consent, and (5) interference in the early attachment between the newborn and his or her mother without medical justification, denying the mother the possibility of carrying and nursing the newborn immediately after birth (…)”. |
| **Items:** Observation of evidence-based and respectful maternity care practices: 1. Fetal Heart Rate monitored at least 15 min prior to birth, 2.Administration of a uterotonic soon after birth (Step 1 of AMTSL1) , 3. Birth of the placenta by controlled cord traction (Step 2 of AMTSL1), 4. Uterine massage (Step 3 of AMTSLa ), 5. Placenta examination after birth, 6. Newborn dried and stimulated within 30 seconds after born, 7. Immediate Skin to Skin contact, 8. Delayed Cord Clamping, 9. Breastfeeding prior to leaving the birth room (60 min), 10. Non-insertion of IV cannula during labour, 11. Labour induction, 12.No Kristeller manoeuvre, 13.No Episiotomy, 14.No Manual Uterine Exploration. Women autonomy and confidentiality: 1. Privacy (gowns, curtains or doors) during labour, 2.Freedom of movement, 3.Oral liquids intake allowed during labour, 4. Freedom to choose the position of birth. Dignified care: 1.Provider communicates with the woman by name, 2.Provider verbal communication is positive, 3.Provider nonverbal communication is positive, 4.A companion is allowed during labour and birth. Informed consent: 1. Women signs informed consent, 2.Provider explains procedures, 3.Provider explains medications, 4.Provider gives the woman all of the information she requests Provider addressed the woman’s questions or concerns during labour Provider explains post-partum care, 5.Provider explains newborn care | | | | | | |
| (56) | Bearing witness: United States and Canadian maternity support workers’ observations of disrespectful care in childbirth | United States of America and Canada, North-America | To "compare birth doulas' and labour and delivery nurses' reports of witnessing disrespectful care in the United States and Canada". | 2781 (967 nures, 1435 doulas) | Nurses, doulas | “Three typologies of disrespectful care: 1. verbal abuse including threats of poor outcome, racially demeaning comments; sexually degreeding remarks. 2.stigma and discrimination: extra procedures because of race/ethnicity. 3. failure to meet professional standards of care: failure to secure fully informed consent or performing procedures explicitly against a women´s wishes (…)”. |
| **Items:** Verbal abuse:Have you ever witnessed a care provider tell a woman that her baby might die if she does not agree to a proposed procedure? Have you ever heard a care provider mention a laboring woman’s racial or ethnic background in a way that was demeaning? Have you witnessed a care provider use sexually degrading language with a laboring woman? Stigma and Discrimination: Have you observed a laboring woman receive more procedures because of her racial or ethnic background? Faulure to meet professional standards of care: Have you witnessed a care provider engage in procedures without giving the woman a choice or time to consider the procedure? Have you witnessed a care provider engage in procedures explicitly against the wishes of the woman? | | | | | | |
| (57) | Associations between perceptions of care and women's childbirth experience: a population-based cross-sectional study in Rwanda | Rwanda, Africa | To "investigate how women's overall childbirth experience in Rwanda was related to their perception of childbirth care". | 921 | Women wo had given birth 1 to 13 months earlier. | “A positive childbirth experience is important for the woman’s wellbeing, facilitates the mother-child bonding and may have implications for the future health for both the mother and baby. On the contrary, a negative experience increases the risk for postpartum depression, secondary fear of childbirth and post-traumatic stress disorder”. |
| **Items**: 1. I had confidence in the medical skills of the staff during childbirth, 2. I got information on what was happening during childbirth, 3. the health care staff treated me with respect during childbirth, 4.I got the pain relief I needed during childbirth, 5. I got the support from the health care providers that I needed during childbirth, 6.the health care providers helped me start breastfeeding, 7.I had my baby skin to skin after birth | | | | | | |
| (58) | Disrespect and abuse during facility-based childbirth in a low-income country. | Nigeria, Africa | To "determine the prevalence and pattern of disrespectful and abusive care during faciloty-based childbirth in Enegu, Nigeria". | 446 | Women wo had delivered up to 6 weeks before participation at the hospital and were now at the immunization clinic for their newborns. | “(…) seven categories of attributes that effectively defined disrespectful and abusive care in facility-based skilled childbirth: physical abuse, non-consented care, non-confidential care, non-dignified care, discrimination, abandonment/ neglect of care, and detention in facilities until hospital bills are paid (…)“. |
| **Items:** Non-consented care: episiotomy, augmentation of labour, shaving of pubic hair, sterilization, Cesarean delivery, blood transfusion. Physical abuse: restrained or tied down during labour, episiotomy given or sutured without anesthesia; beaten, slapped, or pinched; sexually abused by health worker. Non-dignified care: Blamed or intimidated during childbirth; Threatened with cesarean delivery to discourage patient from shouting; Received slanderous remarks (aspersions) from birth attendant; Scolded, shouted at, or called stupid; abandonment/neglect of care: Denied companionship by the husband or close relatives; Being left unattended in second stage of labor; Birth attendant failed to intervene in a life-threatening situation; Not granted requested attention because staff was exhausted. Non-confidential care: Age disclosure without consent, Provision of care without privacy, Medical history disclosure without consent, Disclosure of HIV status without consent. Detention in the health facility: Discharge postponed until her hospital bills are paid, Detained in the hospital until infant’s bills are paid. Discrimination on the basis of specific patient attributes: Denial of needed attention on the basis of ethnic origin, Denial of needed attention because of low social class, Denial of needed attention because of teen age (≤19 years), Denial of needed attention because of HIV-seropositive status | | | | | | |
| (59) | Birth experiences of primiparous Turkish women: public and private hospitals | Turkey, Asia, Europe | To "better understand primiparous women's childbirth experiences in private and public hospitals to shed light on the reasons for women's preference of caesearean sections". | 240 (120 in private hospital, 120 in public hospital) | Primiparous women admitted for postpartum care, between 18-35 years of age, married, primiparous, delivered a singleton infant between gestational weeks 38-40 | “A woman’s experience of labour and birth may have long-lasting and profound effects on her wellbeing and that of her baby and husband.2 Further, the childbirth experiences of primiparous women are especially important because of their impact on future births, most especially if the first birth is a caesarean section. There is also an impact on the nature of the birth stories that are told to subsequent generations. Negative childbirth experiences often lead women to prefer caesarean sections to vaginal birth (…)”. |
| **Items:** Supportive practices: Position and mobility, maintaining oral intake, skin to skin contact, midwife support, gynecologist support, protection of the privacy. Women's perception of pain, anxiety and fear during labour and birth | | | | | | |
| (60) | The impact of birthplace on women's birth experiences and perceptions of care | Denmark, Europe | To "compare women’s birth experience, care satisfaction and perception of specific patient-centred care elements in two FMUs versus two OUs and to explore the influence of specific medical and socio-demographic factors on women’s birth experience". | 375 | 185 low-risk women receiving reestanding midwifery units care and a matched control group of 190 low-risk women receiving standard obstetric units care. | “While positive birth experiences contribute to women’s feeling of accomplishment and self-esteem and lead to psychological growth, empowerment, and easier adaptation to motherhood (…), negative experiences are associated with a number of complications such as postpartum anxiety, depression, post- traumatic stress syndrome (…) fear of childbirth (…), reduced future reproduction (…),and request for caesarean section (…) Four key dimensions of patient-centred care (…)have been identified as prominent aspects of the childbirth experience: the woman’s perceptions of intrapartum support, participation in decision-making, information, and control“. |
| **Items:** 1.Overall birth experience,2.Care satisfaction, 3.Support from midwife,4.Midwife present when wanted, 5.Attention to psychological needs, 6.Feeling of being listened to7.Level of information, 8. Participation in decision-making, 9. Consideration for birth wishes, 10.Suggestions for pain-relief, 11.Staff support for partner, 12. Undisturbed contact with newborn, 13.Support provided by partner, 14. Loss of control over labour/reactions, 15.Loss of control over staff actions | | | | | | |
| (61) | Women's experiences of maternity care in England: preliminary development of a standard measure | England, Europe | To "develop a valid and reliable self-report measure of the Experience of Maternity Care (EMC) that examines salient aspects of experience retrospectively related to (i) pregnancy (ii) labour and birth and (iii) the early postnatal period". | 504 | Women who gave birth in 2016. Women experiencing a perinatal loss and young mothers less than 16 years of age were excluded. | “Positive experiences during this time can be looked back upon fondly, empowering the woman in her role as a mother, and strengthening her emotionally during her transition to motherhood (…) Conversely, a negative maternity experience may significantly increase the risk of negative health outcomes for the mother such as postnatal mental health disorders with possible long-lasting effects on the mother, the child, and the family system as a whole. The care a woman receives during the perinatal period can have a profound impact on her overall maternity experience, with potentially significant implications for her health and wellbeing both at the time and subsequently (…) In turn, this can impact on the mother-baby relationship and also on the health and wellbeing of the baby (…).A woman’s experiences and memories of maternity care might also influence her decision-making regarding future pregnancies, requests for medical intervention during future childbirth, as well as having an im- pact on future reproduction in general. Thus, it is necessary to monitor, evaluate and optimise the care that women and their families receive during this important time”. |
| **Items**: Staff communicated well with me during labour and birth. I needed more staff support during labour and birth. Everything was explained to me well during labour and birth. I was treated as an individual by staff. I was not involved enough in decisions about procedures that were carried out (e.g. breaking waters, epidural, caesarean section). Health professionals left me alone more than I would have liked. I felt that my pain relief needs were not managed well. I felt safe in the labour and birth environment. The staff could have done more to help me to feel in control of my labour and birth. I had confidence and trust in the staff caring for me. I did not mind being looked after by midwives or doctors I had not met before. I had the best possible care during labour and birth. I received enough care and attention from staff on the postnatal ward. I stayed in hospital as long as I wanted after the birth. I was treated as an individual by midwives/doctors after the birth. After I had given birth, health professionals treated me as though I was no longer important. I had enough information from health professionals about how to care for my baby. I was able to build a good relationship with the healthcare professional(s) I saw after coming home. I was not given the advice and information I needed by health professionals after my baby was born. There was not enough time to talk over my concerns with health professionals. I had all the checks I needed after the birth. After the birth of my baby, I knew who to contact if I had questions or concerns. The postnatal care I received did not meet the needs of me and my baby. Overall I was very pleased with the quality of my postnatal care. | | | | | | |
| (62) | Direct observation of respectful maternity care in five countries: A cross-sectional study of health facilities in East and Southern Africa | Ethiopia, Kenya, Madagascar, Rwanda and Tanzania, Africa | To "provide a descriptive overview of the quality of respectful maternity care in diverse facility settings in East and Southern Africa". | 2164 (labor and delivery observations) | Women (no further information applicable) | „Seven rights of childbearing women from Respectful Maternity Care Charter (…) Article 1. Every woman has the right to be free from harm and ill treatment., Article 2. Every woman has the right to information, informed consent and refusal, and respect for her choices and preferences, including companionship during maternity care. Article 3. Every woman has the right to privacy and confidentiality. Article 4. Every woman has the right to be treated with dignity and respect. Article 5. Every woman has the right to equality, freedom from discrimination, and equitable care. Article 6. Every woman has the right to healthcare and to the highest attainable level of health. Article 7. Every woman has the right to liberty, autonomy, self-determination, and freedom from coercion”. |
| **Items:** Right to information, informed consent and refusal, and respect for her choices and preferences: 1. Explains procedures before proceeding, 2.Informs client of findings,3.Asks client if she has any questions, 4.Provider explains what will happen during labor to client.Right to choose choose evidence-based, respectful, client-focused care practices: 5.Encourages client to have support person, 6.Provider encourages client to consume food and fluids during labor, 7.Provider encourages or assists client to ambulate and assume different labor positions. Right to privacy and confidentiality: 8. Delivery in rooms with auditory and visual privacy, 9. Provider drapes client before delivery.Right to be treated with dignity and respect: 10. Greets client in a respectful manner, 11.Provider supports client in friendly way during labor | | | | | | |
| (63) | The prevalence of disrespect and abuse during facility-based childbirth in urban Tanzania | Tanzania, Africa | To "assess the prevalence of D&A as reported by women who delivered in a large, urban referral hospital in Dar es Salaam, Tanzania, and as observed by trained individuals". | 208 for observation, 1914 for interviews | All pregnant women 18 years and above and admitted to the study facility for labor and delivery services during the period of data collection were eligible for this study. | “D&A is also a fundamental violation of women’s human rights and undermines the safety and effectiveness of health systems. (…) seven categories of disrespect and abuse (D&A) during childbirth emerged from qualitative and anecdotal reports: physical abuse, non-consented care, non-confidential care, non- dignified care, discrimination, abandonment, and detention in health care facilities (…)”. |
| **Items:** Physical abuse: Kicked, pinched, slapped, episiotomy without anesthesia, pushed, raped, other. Non-dignified care: Shouted at, scolded, threatened to withhold services, laughed at or scorned, other. Non-consented care: Tubal ligation, hysterectomy, abdominal palpation, vaginal examination, episiotomy, other. Non-confidential care: HIV status shown to others, other health information shown to others, HIV status shown to non-health staff, health information discussed with non-health staff, personal issues discussed in earshot of others, other. Abondonment: While in labor, while delivering, while experiencing a complication, after delivery, other. Lack of privacy: Uncovered during delivery or examination, no screens blocking view during delivery or examination. Detention: any. Observation: Physical abuse: Episiotomy performed without anesthesia given. Non-consented care: Lack of consent for first examination in antenatal ward, Lack of consent for vaginal examination in antenatal ward. Non-confidential care: Mother’s history taking findings shared when others could hear. Lack of privacy: No partitions separating beds in antenatal ward; partitions do not give privacy in antenatal ward, mother not covered during examination in antenatal ward, mother not covered while being moved from antenatal ward to delivery room, mother not covered during delivery Partitions not closed during delivery, mother not well covered after third stage of labor, mother not given a bed to herself in post-natal ward, no partitions/curtains between beds in post-natal ward, no partition/curtain during post-natal examination, if done/ Mother not covered during post-natal examination, if done.Non-dignified care: Mother not welcomed in a kind and gentle manner, use of non-dignified language during history taking, use of harsh tone or shouting during history taking, bed in post-natal ward not clean, bed in post-natal ward not covered with a bed sheet | | | | | | |
| (64) | The prevalence of disrespect and abuse during facility-based maternity care in Malawi: evidence from direct observations of labor and delivery | Malawi, Africa | To "measure the prevalence of disrespect and abuse during labor and delivery through the secondary analysis of direct clinical observations and to describe the association between the observation of D&A items with the place of delivery and client background characteristics". | 2109 direct clinical observations | Women and health workers. | „While disrespect and abuse during delivery does not necessarily mean that respectful care was provided, it does mean that the fundamental human right of women to receive the highest attainable standard of care was violated (…)”. |
| **Items:** Non dignified care: Did not respectfully greet pregnant woman, shouted, insulted or threatened the woman during labor or after. Non consented care: manual exploration of uterus after delivery when unindicated, used episiotomy (without indication).Did not ask woman (and support person) if she has any questions. Did not ask client if there are any other problems the client is concerned about. Did not explain procedures to woman (support person) before proceeding. Did not inform the woman what will happen before conducting the vaginal examination. Did not inform pregnant woman of findings. Did not explain what will happen in labor to woman (support person) at least once. Did not explains procedures to woman (support person) before proceeding. Provider did not give at least one update on status and progress of labor. Non confidential care: Woman did not have audio and visual privacy. Provider did not drape woman (one drape under buttocks, one over abdomen). Woman did not have her own bed.Provider did not use curtains or other visual barriers to protect woman during exams, births, procedures. Abandonment or denial of care: Provider did not encourage the woman to have a support person present during labor and delivery. Provider did not encourage woman to consume fluids/food during labor at least once. Provider did not encourage or assist woman to ambulate and assume different positions during labor at least once. Provider did not ask woman which position she would like to deliver in. Support person or companion for mother was not present at birth. If support person was not present at birth: Support person was restricted from being present. Woman requested some pain relief for her pain but was not given anything. Woman was not allowed to deliver in her preferred birthing position (if she had a preferred position). Mother and newborn were not kept in same room after delivery (rooming-in). Physical abuse: Provider slapped, hit or pinched the woman during labor or after | | | | | | |
| (65) | Respectful maternity care in Ethiopian public health facilities | Ethiopia, Africa | To "measure the prevalence of RMC and mistreatment of women in hospitals and health centers and to identify factors associated with the observed RMC and mistreatment of women in Ethiopia, including facility- and provider- related factors". | 240 women (175 in health center and 65 in hospitals) | Women and health professionals. | “The White Ribbon Alliance defines RMC as an approach that emphasizes the positive inter- personal interactions of women with health care providers and staff during labor, delivery, and the postpartum period. Absence of D&A by health care providers and other staff alone is not sufficient for provision of RMC; the RMC definition calls for fostering positive staff attitudes and behaviors that are conducive to improved satisfaction of women with their birth experience”. |
| **Items:** Respectful maternity care: receives and greets the pregnant women, explains each step of the examination to the women, encourages the women to ask questions, responds to a women/companion question politely, explains what will happen in labor to women, encourages women to walk and change position, at least once ensures if she has taken light food, asks women which position she would like to deliver, allowed to give birth in the position she wants. Mistreatment: Physical abuse, Verbal abuse, Privacy violated, Abandonment: or being left alone | | | | | | |
| (66) | Mistreatment of women in public health facilities of Ethiopia | Ethiopia, Africa | To "generate evidence on the prevalence of mistreatment of women in public health facilities as reported by women in Ethiopia and identify factors that may contribute to such mistreatment". | 379 | Women who had used skilled birth attendance services in public health facilities from 6 hours to 3 months prior to the start of data collection were included and interviewed about their birthing experiences. | “Physical abuse included hitting, slapping or pinching. Verbal abuse included shouting, scolding, threatening to take women into the operating theatre or addressing women using insulting names. Failure to meet standards of care included neglecting women when they needed care at some point during labor and childbirth, ignoring women’s requests for pain relief, providing treatment without consent and providing care that violated privacy of women. Poor rapport between women and providers included not greeting women, not explaining the labor progress, not responding to women’s questions in a polite manner, not encouraging women to move around freely, not allowing women to bring a companion, not allowing women to give birth in their preferred birth position and not offering hot drinks or food after childbirth“(…)”. |
| **Items**: Physical abuse included: hitting, slapping or pinching. Verbal abuse included shouting, scolding, threatening to take women into the operating theatre or addressing women using insulting names.Failure to meet standards of care included neglecting women when they needed care at some point during labor and childbirth, ignoring women’s requests for pain relief, providing treatment without consent and provid- ing care that violated privacy of women. Poor rapport between women and providers included not greeting women, not explaining the labor progress, not respond- ing to women’s questions in a polite manner, not encouraging women to move around freely, not allowing women to bring a companion, not allowing women to give birth in their preferred birth position and not offering hot drinks or food after childbirth. | | | | | | |
| (67) | The association between disrespect and abuse of women during childbirth and postpartum depression: Findings from the 2015 Pelotas birth cohort study | Brazil, South America | To "examine the effect of the different types of disrespectful and abusive experiences (verbal abuse, denial of care, physical abuse, and undesired procedures) on maternal postpartum depression occurrence and, given that antenatal depression is a strong predictor of depression in the postpartum period and can influence how women perceive, internalize or justify experiences and to explore if the associations differ according to women's antenatal depressive symp- toms status". | 3065 | Women resident in the urban area of Pelota with estimated delivery date in 2015 | “Women ́s experiences of disrespect and abuse often results from the nature of patient-provider interactions in the context of obstetric care and can be expressed as verbal, physical or sexual abuse, stigma and discrimination, neglect, and failure to meet standards of care and attention – such as privacy and confidentiality breaches, limiting access to information and medical procedures con- ducted without consent (…). They have also been linked to the institutional structures and processes that frame the practice of obstetric care in health systems and the persistence of structural gender inequalities in society being considered by some authors as a dimension of violence against women (…)”. |
| **Items:** a) Physical abuse: “Has any professional ever pushed, hurt, beat, or held yourself strongly or conducted any examinations rudely or disrespectfully?” b) Verbal abuse: “Has any professional been rude to you, cursed you or yelled at you, humiliated you or threatened not to assist you?” c) Denial of care: “Has any professional refused to give you anything that you asked for, such as water or painkillers?”d) Undesired obstetric procedures: “Has any professional ever con- ducted any procedure against your will, without explaining the need to conduct it, such as episiotomy or medication to induce labor?” | | | | | | |
| (68) | Prevalence of disrespect and abuse during facility based child birth and associated factors, Jimma University Medical Center, Southwest Ethiopia | Ethiopia, Africa | To "quantitatively describe the level and types of disrespect and abuse women faced during facility-based childbirth". | 290 | All women who gave birth vaginally during the study period. | “Seven categories of disrespect and abuse during child- birth are physical abuse, non-dignified care, discrimination based on specific patient attributes, non-consented care, non-confidential care, abandonment of care and detention in facilities. However, Numerous factors (individual and community-level) may contribute to the experiences of disrespect and abuse. Lack of legal and ethical foundations to address D&A, normalizing D&A, lack of standards and accountability, lack of leadership commitment, and provider prejudice due to training and lack of resources are some among many factors (…)”. |
| **Items:** Physical harm or ill-treatment: mother did not cared for in a culturally appropriate way, mother denied food/fluid without medical indication, mother did not receive pain-relief as necessary, mother and newborn were separated without medical indication, mother was physically confined, physical force was used (e.g. slapping/hitting the mother). Non-consented care: service provider did not introduce him/herself to the mother, mother was not encouraged to ask questions consent or permission prior to any procedure not obtained, service provider did not explain what is being done and expected outcome during labor and birth, periodic updates on status and progress of labor not given, service provider did not answer questions promptly, politely and truthfully, mother not allowed to move about during labor, mother not allowed to take position of choice during childbirth. Non-confidential care: curtains or other visual barriers not used non-dignified care: service provider did not speak politely, mother was insulted, intimidated, threaten, or coerced. Discrimination on specific grounds/ characteristics: mother shown disrespect based on religion or ethnicity or place of residence, etc., a language or language-level that mother cannot understand was used. Abandonment or denial of care: provider did not arrive quickly when called, mother was not encouraged to call provider if needed, mother was left alone or unattended. Detention or confinement in facilities: mother was delayed in health facility against her will. | | | | | | |
| (69) | A questionnaire to measure women's experiences with pregnancy, birth and postnatal care: instrument development and assessment following a national survey in Norway. | Norway, Europe | To "describe the development and the psychometric properties of the pregnancy- and maternity-care patients’ experiences questionnaire (PreMaPEQ)". | 4904 | Women who gave birth in the last quarter of 2011 in a Norwegian institution and who were 16 years old or older were included. | “Collection of patient-reported outcomes, including patient experiences, is an important aspect of evaluations of health services. (…) These surveys call for descriptions of mainly non- technical aspects of the health-care services and may in- volve different target populations, such as the general population, broad groups of service users, or patients with specific conditions (…)”. |
| **Items:** Options for pain relief during the birth. Post-natal period (e.g. breastfeeding, nutrition, care for the child). Personal relationships in the delivery ward. Were you treated politely and with respect by the health-care personnel at the delivery ward? Did you find that the health-care personnel were open to your questions? Did you find that the health-care personnel cared about you? Attention to partner in the delivery ward: Was your partner received well by the health-care personnel at the delivery ward? Were things arranged so that your partner could be present if you both so wished? Resources and organization in the delivery ward: Were you received well when you arrived at the delivery ward? Did the health-care personnel have time for you when you needed it?Did you have confidence in the health-care personnel’s competence?, Did you receive sufficient information during your stay at the delivery ward? Did you find that the services you received during your stay at the delivery ward were well-organized?, Did you find that the health-care personnel cooperated well during the birth? Did you receive information about who had the main responsibility for you? Personal relationships during your postnatal stay: Were you treated politely and with respect by the health-care personnel during your postnatal stay? Did you find that the health-care personnel were open to your questions? Did you find that the health-care personnel cared about you and your child?Attention to partner during your postnatal stay: Was your partner received well by the health-care personnel during your postnatal stay? Were things arranged so that your partner could be present if you both so wished? Information about women’s health during your postnatal stay: Did you receive sufficient information about the following? Your physical health after giving birth, Any possible mood changes after giving birth. Information and guidance about your child during your postnatal stay. Did you receive sufficient information about the following?: Breastfeeding and other ways of feeding the child. Child care. Resources and organization during your postnatal stay: Did the health-care personnel have time for you when you needed it? Did you have confidence in the health-care personnel’s professional competence? Did you find that the services you received during your postnatal stay were well organized? Did you find that the health-care personnel cooperated well during your postnatal stay? Did you receive information about who had the main responsibility for you? Were things arranged so that you could get enough peace and rest? Personal relationships in the public health clinic: Are you treated politely and with respect by the staff? Do you find that the staff are open to your questions? Do you find that the staff care about you and your child? Information about women’s health in the public health clinic: Did you receive sufficient information about the following? Your physical health after giving birth, Possible mood changes after giving birth. Information about your child in the public health clinic: Did you receive sufficient information about the following?: The child’s development and health, Vaccines for the child, Breastfeeding and other ways of feeding the child, Child care. Resources and organization in the public health clinic: Do the staff spend enough time at the check-ups? Do you have confidence in the professional competence of the staff? Do you find that the care you receive at the health clinic is well organized? | | | | | | |
| (70) | Institutional violence and quality of service in obstetrics are associated with postpartum depression | Brazil, South America | To "investigate the association between institutional violence in obstetrics and postpartum depression and the potential effect of race, age, and educational level in this outcome". | 432 | Mother-baby pairs. The inclusion criteria were: mother with children younger than three months of age on the vaccination day. | “(…) Among the problems related to the health of pregnant women, concerns have been raised more recently regarding certain practices adopted in medical assistance, referred to by specialists as ‘institutional violence in childbirth’ or ‘obstetric violence’ (…) institutional violence is defined as the failure to act or any type of omission in health care services. This ranges from the broad level of lack of access to these services to their bad quality. (…) Some epidemiological studies have associated the occurrence of psychiatric disorders in the puerperal period, among them postpartum depression, with elements related to obstetrical care (…) such as feeling of abandonment during delivery, inadequate pain management, frustration for having delivered via cesarean section when natural childbirth was possible, and the pregnant woman’s perception of the team who provided the care”. |
| **Items:** Physical violence was defined as the answer yes to the questions: During childbirth, did any of the health care professionals: a) hurt you during the vaginal exam?; b) hit you?; c) push you?; and, d) tie you? Violence by negligence was defined as the answer yes to the questions: a) Did the health care professionals deny you pain relief ?; b) Did any health care professional deny assistance?; and, c) Did any health care professional not explain the procedures he or she was doing to you? Verbal violence was defined by the answer yes to any of the questions: During delivery, did any of the health professionals: a) yell at you?; or said something similar to b) Don’t cry! Next year you will be here again; c) When you were making the baby, you didn’t cry, or called your mommy. Why are you crying now?; d) If you keep screaming, I will stop what I’m doing and I won’t come back again; and, e) If you keep screaming, you will harm your baby. It will be born deaf. Violence from the institution: Were you not allowed a companion of your own choice during labor?; b) during childbirth?; and, c) after childbirth? Violence from the health system: Did you go to more than one hospital to find a bed for childbirth? Did your delivery not happen in the hospital originally recommended? | | | | | | |
| (71) | Ethical Implications of Obstetric Care in Hungary: Results from the Mother-Centred Pregnancy Care Survey | Hungary, Europe | To "see the frequency of selected interventions (birth induction, cae- sarean section, episiotomy, forced supinal position during birth, and the consent process associated to these interventions". | 1257 | women (with childbearing capacity) between the age of 18 and 45 with children under the age of 5 were surveyed online. | "Informed consent plays an important role in clinical decision making. It is a basis of self determination in health care. In ideal situations health care professionals inform their patients about all relevant aspects of care and alternative care options, map the value system of the patients, and adjust the information process accordingly. Patients and health care professionals have shared responsibility in the process, both parties have obligations and responsibilities but apart from some exceptional situations the ultimate decision is in the hands of patients". |
| **Items:** Mother-Centred Pregnancy Care’ questionnaire (only issues related to respectful care are addressed (informed consent and autonomy). The majority of the items were selected from two previously validated English-language maternity care surveys, Listening to Mothers III and Changing Childbirth in BC). 1. Before your caesarean, did your doctor ask for your permission? 2. Before your induction, did your provider ask your permission? 3. Before your episiotomy did you doctor ask for your permission? 4. Was the birth position freely chosen by you? | | | | | | |
| (72) | Development and Assessment of Respectful Maternity Care Questionnaire in Iran | Iran, Asia | To "develop and test the Quality of Respectful Maternity Care Questioner (QRMCQI) in Iran, condurc conent and face validity, assess ist reliability, and evaluate it based on factor analysis for assessing RMC by asking the clients who were women who had received care during labor, delivery and post-partum". | 453 | Women wo had delivered a baby and no experience of angusihed feeling, critical situation, severe stress or hig anxiety that affected the subject mentally in about the last eight weeks | “Over recent years, promotion of the usage of Respectful Maternity Care (RMC) has been developed gradually, emphasizing the importance of underlying professional ethics and considering psychological, social and cultural aspects of health care delivery as essential elements of care (…). While medical treatment is only one aspect of RMC, failure to focus on the well-being of women and newborns by imposing unnecessary or harmful practices can be considered abusive and disrespectful (…)”. |
| **Items:** LABOUR: 1. Did the service providers introduce themselves to you? 2. Did the staff adress you with your name? 3. Was the staff attitude with you politely? 4. Did you have the right to choose? 5. Did the staff request you to ask your questions? 6. Did the staff request your campanion to ask questions? 7. Did the staff disagree with your beliefs? 8.Did te staff diagree with you companions beliefs? 9.Did the staff speak to you in a language you easily understand? 10. Any insults or threats happened against you? 11. Any insults or threats happened against your companion? 12.Did you forced to do what you did not want? 13. Did your companion forced to do what he/she did not want? 14.Were your questions answered politely? 15.Have your questions answered honestly? 16.Did the staff screamed over you? 17. Where you allowed to take the hands of the staff or touch them? 18.Did the care provider and staff consider your comfort? 19.Was your privacy maintained during the medical examinations using the curtain? 20.Have you ever been told that your recorded information will be kept safe and secure (so that no one can access it)? 21.Have you explained what to do? 22.Did the staff explain what migt happened to you in the labor process? 23. Did the staff explain to you before any action and practice? 24.did the staff ask for your permission b3fore any action and practice? 25. did the staff have regular monitoring of your delivery progress? 26. Did they limit your drinking? 27.Did they limit your eating? 28.Did they insure you physically? 29. Have you been forced to stay in your bed? 30. Were you allowed to move? 31.Were you allowed to choose your desired status on the bed or outside it? 32.If the discomfort was felt, would the care providers fix it? 33.Of the pain was expressed, would the care providers resolve it? 34.Did the stadff use non-pharmacological pain relief methods (massage, heat therapy, hot water compression, ice compress, aroma therapy, pressure on the waist region) for you? 35. Did the care provider use pain reliver (oxytocin) in your serum? 36.Were you allowed to choose the type of delivery? 37.Did the staff describe the benefits oif you chosen delivery type? 38. Did the staff describe the disadvantages of your chosen delivery type? 39. Did the staff suggest you the physiological delivery /(without intervention)? 40. Did the staff offer you a cesarean? 41. Were you discriminated because of ethnicity, race, economic situation and etc. from the rest of admitted women? DELIVERY: 42.Did the staff describe to you about what you need to do? 43.Did the staff explain to you about possible events that may have occured in the process of delivery? 44. Have you been allowed to choose your desired condition in the bed during childbirth? 45.Did the staff manage your privacy using a curtain or parlor during the delivery, whether in the public room or in private? 46.Have you been informed that you have permission to attend a trained female companion during childbirth? 47.Did the staff ask for your permission before any action and practice? 48.Was the skin of the baby contacted with your skin immediately after birth? 49.Was you tendency considered in case of skin contact with your baby immediately after birth? 50. Was the skin contact with your baby last for the first hour of birth? 51.Did you breastfeed your baby during the first hour? POST-PARTUM: 52.Were you admitted to your baby during the whole day in a room? 53.Did the staff provide you ecessary explanations for breastfeeding your baby? 54.Did the staff force you to breastfeed your baby? 55.Did the staff tell you to call them if you need help? 56. Were the staff quickly in responding to your need in case of any help and information? 57. Have you ever been completely abandoned and keep alone? 58. Did you have permission to attend a companion with yourself? 59. Have you been hospitalized too much due to the delay or non-payment of the related costs? | | | | | | |
| (73) | Disrespect and abuse during childbirth in Western Ethiopia: Should women continue to tolerate? | Ethiopia, Africa | To "examine the prevalence and associated predictors of D&A as reported by women during labor and delivery in public health facilities of western Oromia region in South- western Ethiopia". | 612 | NA | “(…) Types of D&A were then categorized as physical abuse, non-dignified care, abandonment, non-consented care, non-confidential care, detention and discrimination.” |
| **Items:** Physical abuse: Hitting (slapped, beaten or pinched), harshly forcing legs apart, tied down during labor. Non-dignified care: Shouted at, threat of withholding treatment, blamed or intimidated. Non-consented care: Non-consented episiotomy, C-section, tubal ligation. Non-confidential care: Provision of care without privacy, medical history disclosed without consent. Neglect/abandonment: Gave birth outside delivery room (corridor, waiting room or floor), ignored when needed help, delivered without skilled attendant. Detention: Detention in health facility for failure to pay, request for bribe. Discrimination: Denial of needed attention on the basis on areas of residence (urban/rural), denial of needed attention on the basis of age (old/young), denial of needed attention on the basis of occupation/education. | | | | | | |
| (74) | Intrapartum midwifery care impact Swedish couple's birth experiences - A cross-sectional study | Sweden, Europe | To "describe and evaluate uniformity in couples’ birth experience and experience of the quality of intrapartum midwifery care". | 209 couples (418 individuals) | To be included in the present study both the mother and her partner had to be able to understand the Swedish language (1-4 years postpartum) | “Childbirth is a highly significant event for the mother, her family and the community (…) The experience of giving birth has long-term implications for mothers’ future emotional, physical and reproductive health and wellbeing.3,4 It has been shown that the experience of childbirth also has an impact on the birth partner’s future emotional, physical and reproductive health and wellbeing. Parents’ birth experiences affect bonding with their child which in turn may influence the child’s future health. (…) Quality of intrapartum care is understood as a resource structure of the care organisation in combination with parents’ preferences and therefore it is important to not only measure satisfaction but also to simultaneously measure the subjective importance accorded the care given”. |
| **Items**: Midwife support during labour and birth: 1. Midwife’s presence in the labour room, 2.Midwife information about labour progress, 3. Mother recieved best possible medical care, 4. Mother recieved wished painrelief, 5.Mother encouraged by midwife during second stage birth position, 6. Mother encouraged by midwife for birth position at birth, 7. I felt involved in decision making. Father involvement: 8. Father overall support of mother, 9.Father supported female partner’s birth position. Perception of control: 10. I felt in control | | | | | | |
| (75) | Disrespect and abuse of women during childbirth in public health facilities in Arba Minch town, south Ethiopia - a cross-sectional study | Ethiopia, Africa | To "assess the prevalence of women’s disrespect and abuse during childbirth in public health facilities in Arba Minch town, south Ethiopia". | 281 | Women giving birth in one of the three public health facilities expect except the ones who underwent elective cesarean section. | “Disrespect and abuse during childbirth is common throughout the world (…). It can occur at the level of contact between the client and the care provider, as well as through systemic failures at the health facility and health system level (…)“. |
| **Items**: Did the birth attendants/the care providers use physical forces (slapping, pinching, beating /hitting) against you while you were in a labor pain? Did the birth attendant(s) threaten you with beating to let you obey their order? Have you tied down on a delivery bed when you were in labor? Did the health care provider(s) suture your perineum? If so, did they use local anesthesia so that it was pain-free? What birthing position do you prefer to give birth? Did the care providers allow you to assume the position of your choice during the current childbirth? Did the birth attendants(s) allow you to move around. (Ambulate) during the course of the labor? If No, have they told you that you have a medical condition or you are in advanced labor or any other reason why they have not allowed you to do so? Did the birth attendants push your tummy down to deliver the baby (used fundal pressure)? Were you restricted from drinking any fluid throughout the labor course? Did the care providers order your caretakers/family to clean the delivery bed/room?Non-confidential care: Did the health care providers use curtains or other physical barriers so that your privacy was kept during the labor and delivery processes? Were other persons apart from the care providers allowed to the room you were giving birth who could observe you while you are naked on the bed? Did the birth attendants share your secret information with other non-concerned persons? Or don’t you trust them that your secret is likely to be shared with others? Non-consented care: Did the care provider introduce him/herself to you and your companion, Did the care providers share the findings of your initial assessment with you and or your families? Did the care providers encourage you to ask questions? Did the care providers(s) explain to you what is being done and what to expect throughout the labor and birth process? Have you undergone an episiotomy? If Yes, did the birth attendant explain the indication and asked your permission/consent before she/he cut? Have you undergone a cesarean section? If Yes, did the care providers explain the indication and asked you to sign consent/ permission? Was your labor augmented? If Yes, did the care providers explain the indication and asked your permission before putting you on the medication/oxytocin? Did you receive blood during the course of labor and delivery? If you were given blood, were you informed about the indication and was your/your families/ permission asked before the procedure is started? Did the care providers coerce you to undergo C/S? Non-dignified care: Did the care provider speak to you politely throughout the course of the labor, Did the care provider intimidate/ humiliate you at least one times? Did the care provider balm you for getting pregnant or shouting/crying due to the pain of the labor? Did the care provider shout at you to calm you down? Did the care providers allow your companion to enter the delivery room? Perceived discrimination: Did the care provider discriminate you because of your traditional belief? Did the care provider discriminate you because of your religion? Did the care provider discriminate you because of your educational status? Did the care provider discriminate you because you are from rural area/ from a very far distance? Did the care provider discriminate you because you are RVI patient? Did the care providers discriminate you because of your age? Abandonment of care: Have you ever left alone without the care provider nearby you while you were in labor and needed help? Did you give birth in the health institution by yourself because the care providers were not around you? Have you encountered a life-threatening condition for which you have shouted for help but could not get anyone reached you in time? Detention of health facilities: Did the health care providers detain you in the health facility because of payment of because you have pose damage to the property of the health institution? | | | | | | |
| (76) | Validity of a questionnaire measuring the world health organization concept of health system responsiveness with respect to perinatal services in the Dutch obstetric care system | Netherlands, Europe | To "assess the psychometric properties of a newly developed responsiveness questionnaire dedicated to evaluating maternal experiences of perinatal care services, called the Responsiveness in Perinatal and Obstetric Health Care Questionnaire (ReproQ), using the eight-domain WHO concept". | 171 | Women or their partners were required to speak and understand dutch sufficiently. | "Responsiveness addresses non-clinical aspects of health service quality that are relevant regardless of provider, country, health system or health condition. Responsiveness refers to “aspects related to the way individuals are treated and the environment in which they are treated” during health system interactions. The concept excludes the financial and clinical domains of quality and focuses on a set of non-clinical domains that reflect respect for human dignity and the client orientationof the care process and setting.  The concept of responsiveness aims to capture information on the non-clinical quality of the patient’sactual experience in contrast to patient satisfaction questionnaires. Literature has shown that expectations may strongly influence patient satisfaction, which makes international comparisons of non-clinical service quality challenging since expectations are in turn influenced by economic and political influences". |
| **Items:** ReproQ (Responsiveness in Perinatal and Obstetric Health Care Questionnaire) domains: dignitiy, autonomy, confidentiality, communication, propmt attention, social consideration, quality of basic amenities and choice and continuinity) Dignity: Were physical examinations and treatments done in a way that respected your privacy? Did the examination rooms ensure your privacy? Were you treated with respect by your health care provider? Autonomy: How well were you involved in making decisions regarding your examinations or treatments? Were you able to refuse examinations or treatments? Were you asked permission before testing or starting treatment? Confidentiality of information: Were consultations carried out in a manner that protected your confidentiality? Was confidentiality kept on the information provided by you? Was your medical record kept confidential? Communication: How well were things explained by your health care provider in a way you could understand? Was written information provided in such a way you could understand? Were you encouraged to ask questions about your health problems, treatment and care? Were you given time to ask questions about your health problem or treatment? Was information on the health service’s contact, location and parking information clear to you? Prompt attention: How well did you receive prompt attention at your health service? How did you experience the waiting time after you asked for help? How well was the accessibility by phone? How do you rate the travel time to your health service? Social consideration: Did the health care provider facilitate the support of your relatives and friends? Was the home situation taken into consideration when planning an appointment? Quality of basis amenities: How do you rate the quality of the hygiene of the toilets? How do you rate the overall quality of the surroundings, for example, space, seating, fresh air and cleanness? Choice and continuity of health care provider: Were you able to choose your own health care provider? Were you able to use other health care services other than the one you usually went to? How well was the continuity of care by one health care provider? Were you able to choose your own place of delivery? | | | | | | |
| (77) | Quality of perinatal care services from the user's perspective: A Dutch study applies the World Health Organization's responsiveness concept | Netherlands, Europe | To "assess responsiveness outcomes, their importance and factors influencing responsiveness outcomes during the antenatal and delivery phases of perinatal care". | 171 | Women or their partners were required to speak and understand dutch sufficiently. | "Responsiveness addresses non-clinical aspects of health service quality that are relevant regardless of provider, country, health system or health condition. Responsiveness refers to “aspects related to the way individuals are treated and the environment in which they are treated” during health system interactions. The concept excludes the financial and clinical domains of quality and focuses on a set of non-clinical domains that reflect respect for human dignity and the client orientationof the care process and setting. The concept of responsiveness aims to capture information on the non-clinical quality of the patient’sactual experience in contrast to patient satisfaction questionnaires. Literature has shown that expectations may strongly influence patient satisfaction, which makes international comparisons of non-clinical service quality challenging since expectations are in turn influenced by economic and political influences". |
| **Items:** ReproQ (Responsiveness in Perinatal and Obstetric Health Care Questionnaire) domains: dignitiy, autonomy, confidentiality, communication, propmt attention, social consideration, quality of basic amenities and choice and continuinity) Dignity: Were physical examinations and treatments done in a way that respected your privacy? Did the examination rooms ensure your privacy? Were you treated with respect by your health care provider? Autonomy: How well were you involved in making decisions regarding your examinations or treatments? Were you able to refuse examinations or treatments? Were you asked permission before testing or starting treatment? Confidentiality of information: Were consultations carried out in a manner that protected your confidentiality? Was confidentiality kept on the information provided by you? Was your medical record kept confidential? Communication: How well were things explained by your health care provider in a way you could understand? Was written information provided in such a way you could understand? Were you encouraged to ask questions about your health problems, treatment and care? Were you given time to ask questions about your health problem or treatment? Was information on the health service’s contact, location and parking information clear to you? Prompt attention: How well did you receive prompt attention at your health service? How did you experience the waiting time after you asked for help? How well was the accessibility by phone? How do you rate the travel time to your health service? Social consideration: Did the health care provider facilitate the support of your relatives and friends? Was the home situation taken into consideration when planning an appointment? Quality of basis amenities: How do you rate the quality of the hygiene of the toilets? How do you rate the overall quality of the surroundings, for example, space, seating, fresh air and cleanness? Choice and continuity of health care provider: Were you able to choose your own health care provider? Were you able to use other health care services other than the one you usually went to? How well was the continuity of care by one health care provider? Were you able to choose your own place of delivery? | | | | | | |
| (78) | Construct and content validity of the Greek version of the Birth Satisfaction Scale (G-BSS) | Greece, Europe | To "evaluate properties of an instrument designed to measure birth satisfaction in a Greek population of postnatal women". | 162 | healthy greek postnatal women aged 22-46, who delivered between week 32-42 at a maternity unit in Athens. Exlusion of mothers who had experienced a stillbirth, perinatal or neonatal death | “Birth satisfaction represents a woman’s subjective and uniquely personal evaluation of her birth experience. This complex, multifaceted construct includes elements of perceived quality of care, coping efficacy and reflections of the birth experience as a whole and in context. Birth satisfaction is thus a retrospective reconstruction related directly to the salient events surrounding the experience of birth (…). The woman’s individual evaluation of her own birth experience is important, as this may be a potent indicator of perinatal mental health outcome; for example, birth trauma, which would be anticipated to be experienced as a negative event, may be associated with the experience and manifestation of postpartum post-traumatic stress disorder (…)”. |
| **Items:** 1) I came through childbirth virtually unscathed. 2) I thought my labour was excessively long. 3)the delivery room staff encouraged me to make decisions about how I wanted my birth to progress. 4)I felt very anxious during my labour and birth 5)I felt well supported by staff during my labour and birth. 6) The staff communicated well with me during labour. 7) I found giving birth a distressing experience. 8) I felt out of control during my birth experience. 9) I was not distressed at all during labour. 10) The delivery room was clean and hygienic | | | | | | |
| (79) | The Giving Voice to Mothers study: inequity and mistreatment during pregnancy and childbirth in the United States | United States of America and Canada, North-America | To "examine how women in the US overall, and among key subgroups, report on mistreatment during pregnancy and childbirth. In addition, we examine the relationships between race and mistreatment in the context of factors that are frequently related to health inequity. The concept of intersectionality is rarely considered during design, analysis or interpretation of public health studies. we aimed to address this gap in this study". | 2138 | Women who experienced at least one pregnancy in the United States between 2010 and 2016, including those currently pregnant. | “(…) ‘mistreatment’ and delineated the phenomena across seven dimensions: physical abuse, sexual abuse, verbal abuse, stigma and discrimination, failure to meet professional standards of care, poor rapport between women and providers, and poor conditions and constraints presented by the health system (…)”. |
| **Items:** Physical abuse (3rd): Use of force (2nd), physical restraint (2nd): 1. You experienced physical abuse (including aggressive physical contact, inappropriate sexual conduct, a refusal to provide anesthesia for an episiotomy, etc.) (GVtM), Sexual abuse (3rd): sexual abuse (2nd): NA Verbal abuse (3rd): Harsh language (2nd): 2. Health care providers (doctors, midwives, or nurses) shouted at or scolded you (GVtM). Verbal abuse (3rd): threats and blaming (2nd): 3. “Health care providers threatened to withhold treatment or to force you to accept treatment you did not want” (GVtM), 4.“Health care providers threatened you in any other way”. Stigma and discrimination (3rd): discrimination based on socio-demographic characteristics (2nd): 5. Mothers on Respect (MOR) Index (14 items, see Vedam et al.) (GVtM). 17-item Perception of Racism scale: 4 items that assess perceived discrimination from care providers or other disrespectful care provider behaviours, e.g. During my pregnancy I held back from asking questions or discussing my concerns because I felt discriminated against; During my pregnancy I held back from asking questions or discussing my concerns because my care provider used language I could not understand.One item asking women how often they have felt treated unfairly because of their race, heritage or ethnic group (GVtM) Failure to meet professional standards of care (3rd): Lack of informed consent and confidentiality (2nd): “Your private or personal information was shared without your consent” (GVtM), “Your physical privacy was violated (i.e., being uncovered or having people in the delivery room without your consent)” (GVtM). Failure to meet professional standards of care (3rd): Physical examinations and procedures (2nd): “My doctor or midwife explained different options for care during my labour and birth.” (GVtM). Neglect and abandonment (2nd): “My doctor or midwife asked me what I wanted to do before the following procedures were done: (episiotomy, continuous fetal monitoring, screening tests etc).” (GVtM), , “Health care providers ignored you, refused your requests for help, or failed to respond to requests for help in a reasonable amount of time.” (GVtM). Poor rapport between women and providers (3rd): Ineffective communication (2nd): Mother Autonomy in Decision Making scale (MADM) (7 items) (GVtM), (Vedam et al.): Lack of supportive care: Three items that ask women to rate the level of respect, dignity and privacy that their care provider showed during labour and/or birth, Loss of autonomy: Five items about care that women declined, what they declined, why, how their care provider reacted and if anyone helped the woman maintain her wishes. Health system conditions and constraints (3rd): Lack of policies (2nd) and facility culture (2nd): Adapted Perceptions of Racism Scale included items assessing treatment in medical offices and hospital wards | | | | | | |
| (80) | The Mothers on Respect (MOR) index: measuring quality, safety, and human rights in childbirth | United States of America and Canada, North-America | To "describe a person-centered research process to develop a new quality and safety instrument that measures respectful maternity care". | 2271 experiences: Sample 1: 1596 experiences, sample 2: 675 experiences | Women with maternity experiences with a single provider during pregnancy (maternity care experiences for up to two previous pregnancies). Participants from other counties and those where the primary care provider was a nurse or ‘other’ because they did not reflect the primary care provider model in Canada. | not applicable |
| **Items:** The Mothers on Respect index (MORi):14 items on patient-provider communication/respectful maternity care. While making decision during my pregnancy I felt: 1) Comfortable asking questions 2) Comfortable declining care that was offered 3) Comfortable accepting the options for care that my (midwife, doctor) recommended 4) Coerced into accepting the options my (midwife, doctor) suggested (reverse scored) 5) I chose the care options that I received 6) My personal preferences were respected 7) My cultural preferences were respected. During a prenatal visit I held back from asking questions or discussing my concerns: 8)Because my (midwife, doctor) seemed rushed (reverse scored) 9) Because I wanted maternity care that differed from what my (midwife, doctor) recommended (reverse scored) 10) Because I thought my (midwife, doctor) might think I was being difficult (reverse scored) When I had my baby I felt that I was treated poorly by my (midwife, doctor): 11)Because of my race, ethnicity, cultural background or language (reverse scored) 12)Because of my sexual orientation and/or gender identity (reverse scored) 13)Because of my health insurance (reverse scored) 14) Because of a difference in opinion with my caregivers about the right care for myself or my baby (reverse scored) | | | | | | |
| (81) | Prevalence of disrespect and abuse of women during child birth and associated factors in Bahir Dar town, Ethiopia | Ethiopia, Africa | To "assess the prevalence and associated factors of disrespect and abuse of women during childbirth at a health facility in the town of Bahir Dar, Ethiopia". | 422 | Mothers who had given birth at a health facility within the last year in the town of Bahir Dar, Amhara Region | “Disrespect and abuse are defined as any form of inhumane treatment or uncaring behavior toward a woman during labor and delivery (…). Laboring mothers may face various forms of disrespectful and abusive treatment during childbirth at a facility, including physical abuse, lack of consent for care, non-confidential care, undignified care, abandonment, discrimination, and detention in facilities for failure to pay user fees” (…). |
| **Items:** Physical abuse: 1) The provider used physical force/slapped me/hit me. 2)The provider verbally insulted me during labor. 3) I was separated from my baby without medical indication. 4) Support staff insulted me and my companion. 5)The providers did not demonstrate caring in a culturally appropriate way Received unnecessary pain-relief treatment. 6)Denied food or fluids in labor unless medically necessitated. Non-confidential care: 7) The provider did not use curtains or other visual barriers for protecting privacy. 8) Providers discussed my private health information in a way that others could hear. Non-consented care: 9) The provider did not introduce himself/herself to me and my companion. 10) The provider did not encourage me to ask questions-11) The provider did not respond to my questions with promptness, politeness, and truthfulness. 12) The provider did not explain to me what was being done and what to expect throughout labor and birth. 13) The provider did not give me periodic updates on status and progress of my labor.14) The provider did not allow me to assume my position of choice during birth. 15) The provider did not obtain my consent or permission prior to any procedure. Non-dignified care: 16) Providers shouted at or scolded me during labor 17) Providers made negative comments during labor. Abandonment/neglect of care: 18) The provider left me alone or unattened. 19) The provider did not come quickly when I called him/her. Discrimination: 20) Healthcare providers discriminated by race, ethnicity, or economic status. 21) Healthcare providers discriminated because of being a teenager. 22) Healthcare providers discriminated because of being HIV-positive. Detention in a health facility: 23) Discharge was postponed until hospital bills were paid. 24) I was detained in a health facility against my will | | | | | | |
| (82) | Compassionate and respectful maternity care during facility based childbirth and women’s intent to use maternity service in Bahir Dar, Ethiopia | Ethiopia, Africa | To assess "the status of compassionate and respectful maternity care and associated factors in health facility-based childbirth in Bahir Dar town". | 284 | Mothers who had given birth at a health facility within the last year in the town of Bahir Dar, Amhara Region. | “A universal human right that is due to every childbearing woman in every health system around the world in which the maternity care is expanded beyond the prevention of morbidity or mortality to encompass respect for women’s basic human rights, including respect for women’s autonomy, dignity, feelings, choices, and preferences, such as having a companion wherever possible (…)”. |
| **Items:** Friendly care: 1) I felt that health workers cared for me with a kind approach. 2) The health workers treated me in a friendly manner. 3)The HWs was talking positively about pain and relief. 4)The health worker showed his/her concern and empathy. 5)All HWs treated me with respect as an individual. 6)The HWs speak to me in a language that I can understand. 7)The health provider called me by my name. Abuse free care: 8) The health worker responded to my needs whether or not I asked. 9)The health provider slapped me during delivery for different reasons. 10)The health workers shouted at me because I haven’t done what I was told to do. Timely care: 11) I was kept waiting for a long time before receiving service. 12) I was allowed to practice cultural rituals in the facility. 13) Service provision was delayed due to the health facilities internal problem. Discrimination free care: 14) Some of the health workers do not treated me well because of some personal attribute. 15) Some HWs insulted me and my companions due to my personal attributes | | | | | | |

1. Abuya T, Sripad P, Ritter J, Ndwiga C, Warren CE. Measuring mistreatment of women throughout the birthing process: implications for quality of care assessments. *Reprod Health Matters* (2018) 26(53):48-61. Epub 2018/09/14. doi: 10.1080/09688080.2018.1502018. PubMed PMID: 30212308.

2. Afulani PA, Sayi TS, Montagu D. Predictors of person-centered maternity care: the role of socioeconomic status, empowerment, and facility type. *BMC health services research* (2018) 18(1). Epub 2018/05/13. doi: 10.1186/s12913-018-3183-x. PubMed PMID: 29751805; PubMed Central PMCID: PMCPMC5948900.

3. Afulani PA, Diamond-Smith N, Phillips B, Singhal S, Sudhinaraset M. Validation of the person-centered maternity care scale in India. *Reprod Health* (2018) 15(1):147. Epub 2018/08/31. doi: 10.1186/s12978-018-0591-7. PubMed PMID: 30157877; PubMed Central PMCID: PMCPMC6114501.

4. Afulani PA, Feeser K, Sudhinaraset M, Aborigo R, Montagu D, Chakraborty N. Toward the development of a short multi-country person-centered maternity care scale. *International journal of gynaecology and obstetrics: the official organ of the International Federation of Gynaecology and Obstetrics* (2019) 146(1):80-7. Epub 2019/04/21. doi: 10.1002/ijgo.12827. PubMed PMID: 31004349.

5. Alzyoud F, Khoshnood K, Alnatour A, Oweis A. Exposure to verbal abuse and neglect during childbirth among Jordanian women. *Midwifery* (2018) 58:71-6. Epub 2018/01/08. doi: 10.1016/j.midw.2017.12.008. PubMed PMID: 29306737.

6. Asefa A, Bekele D, Morgan A, Kermode M. Service providers' experiences of disrespectful and abusive behavior towards women during facility based childbirth in Addis Ababa, Ethiopia. *Reproductive health* (2018) 15(1):4. Epub 2018/01/07. doi: 10.1186/s12978-017-0449-4. PubMed PMID: 29304814; PubMed Central PMCID: PMCPMC5756390.

7. Asefa A, Bekele D. Status of respectful and non-abusive care during facility-based childbirth in a hospital and health centers in Addis Ababa, Ethiopia. *Reprod Health* (2015) 12:33. Epub 2015/04/19. doi: 10.1186/s12978-015-0024-9. PubMed PMID: 25890317; PubMed Central PMCID: PMCPMC4403719.

8. Attanasio L, Kozhimannil KB. Patient-reported Communication Quality and Perceived Discrimination in Maternity Care. *Med Care* (2015) 53(10):863-71. Epub 2015/09/05. doi: 10.1097/MLR.0000000000000411. PubMed PMID: 26340663; PubMed Central PMCID: PMCPMC4570858.

9. Attanasio L, Hardeman R. Declined care and discrimination during the childbirth hospitalization. *Soc Sci Med* (2019) 232:270-7. Epub 2019/05/22. doi: 10.1016/j.socscimed.2019.05.008. PubMed PMID: 31112918.

10. Azhar Z, Oyebode O, Masud H. Disrespect and abuse during childbirth in district Gujrat, Pakistan: A quest for respectful maternity care. *PLoS One* (2018) 13(7):e0200318. Epub 2018/07/12. doi: 10.1371/journal.pone.0200318. PubMed PMID: 29995939; PubMed Central PMCID: PMCPMC6040717.

11. Bakker R, Sheferaw ED, Stekelenburg J, Yigzaw T, de Kroon MLA. Development and use of a scale to assess gender differences in appraisal of mistreatment during childbirth among Ethiopian midwifery students. *PloS one* (2020) 15(1):e0227958. Epub 2020/01/17. doi: 10.1371/journal.pone.0227958. PubMed PMID: 31945110; PubMed Central PMCID: PMCPMC6964878.

12. Banks KP, Karim AM, Ratcliffe HL, Betemariam W, Langer A. Jeopardizing quality at the frontline of healthcare: prevalence and risk factors for disrespect and abuse during facility-based childbirth in Ethiopia. *Health policy and planning* (2018) 33(3):317-27. Epub 2018/01/09. doi: 10.1093/heapol/czx180. PubMed PMID: 29309598; PubMed Central PMCID: PMCPMC5886294.

13. Bante A, Teji K, Seyoum B, Mersha A. Respectful maternity care and associated factors among women who delivered at Harar hospitals, eastern Ethiopia: a cross-sectional study. *BMC pregnancy and childbirth* (2020) 20(1):86. Epub 2020/02/12. doi: 10.1186/s12884-020-2757-x. PubMed PMID: 32041564; PubMed Central PMCID: PMCPMC7011506.

14. Baranowska B, Doroszewska A, Kubicka-Kraszynska U, Pietrusiewicz J, Adamska-Sala I, Kajdy A, et al. Is there respectful maternity care in Poland? Women's views about care during labor and birth. *BMC pregnancy and childbirth* (2019) 19(1):520. Epub 2019/12/25. doi: 10.1186/s12884-019-2675-y. PubMed PMID: 31870323; PubMed Central PMCID: PMCPMC6929297.

15. Begley C, Sedlicka N, Daly D. Respectful and disrespectful care in the Czech Republic: an online survey. *Reproductive health* (2018) 15(1):198. Epub 2018/12/06. doi: 10.1186/s12978-018-0648-7. PubMed PMID: 30514394; PubMed Central PMCID: PMCPMC6280471.

16. Bekele W, Bayou NB, Garedew MG. Magnitude of disrespectful and abusive care among women during facility-based childbirth in Shambu town, Horro Guduru Wollega zone, Ethiopia. *Midwifery* (2020) 83:102629. Epub 2020/01/26. doi: 10.1016/j.midw.2020.102629. PubMed PMID: 31981935.

17. Bhattacharya S, Sundari Ravindran TK. Silent voices: institutional disrespect and abuse during delivery among women of Varanasi district, northern India. *BMC pregnancy and childbirth* (2018) 18(1):338. Epub 2018/08/22. doi: 10.1186/s12884-018-1970-3. PubMed PMID: 30126357; PubMed Central PMCID: PMCPMC6102865.

18. Bitew K, Ayichiluhm M, Yimam K. Maternal Satisfaction on Delivery Service and Its Associated Factors among Mothers Who Gave Birth in Public Health Facilities of Debre Markos Town, Northwest Ethiopia. *Biomed Res Int* (2015) 2015:460767. Epub 2015/09/09. doi: 10.1155/2015/460767. PubMed PMID: 26347882; PubMed Central PMCID: PMCPMC4546969.

19. Bohren MA, Mehrtash H, Fawole B, Maung TM, Balde MD, Maya E, et al. How women are treated during facility-based childbirth in four countries: a cross-sectional study with labour observations and community-based surveys. *Lancet* (2019) 394(10210):1750-63. doi: 10.1016/S0140-6736(19)31992-0. PubMed PMID: WOS:000496920300030.

20. Brandao T, Canadas S, Galvis A, de Los Rios MM, Meijer M, Falcon K. Childbirth experiences related to obstetric violence in public health units in Quito, Ecuador. *International journal of gynaecology and obstetrics: the official organ of the International Federation of Gynaecology and Obstetrics* (2018) 143(1):84-8. Epub 2018/07/20. doi: 10.1002/ijgo.12625. PubMed PMID: 30025157.

21. Caballero P, Delgado-Garcia BE, Orts-Cortes I, Moncho J, Pereyra-Zamora P, Nolasco A. Validation of the Spanish version of Mackey childbirth satisfaction rating scale. *BMC Pregnancy Childbirth* (2016) 16:78. Epub 2016/04/17. doi: 10.1186/s12884-016-0862-7. PubMed PMID: 27084092; PubMed Central PMCID: PMCPMC4833934.

22. Castro R, Frias SM. Obstetric Violence in Mexico: Results From a 2016 National Household Survey. *Violence Against Women* (2020) 26(6-7):555-72. Epub 2019/04/09. doi: 10.1177/1077801219836732. PubMed PMID: 30957706.

23. Colley S, Kao CH, Gau M, Cheng SF. Women's perception of support and control during childbirth in The Gambia, a quantitative study on dignified facility-based intrapartum care. *BMC pregnancy and childbirth* (2018) 18(1):413. Epub 2018/10/26. doi: 10.1186/s12884-018-2025-5. PubMed PMID: 30352577; PubMed Central PMCID: PMCPMC6199796.

24. Conesa Ferrer MB, Canteras Jordana M, Ballesteros Meseguer C, Carrillo Garcia C, Martinez Roche ME. Comparative study analysing women's childbirth satisfaction and obstetric outcomes across two different models of maternity care. *BMJ open* (2016) 6(8):e011362. Epub 2016/08/28. doi: 10.1136/bmjopen-2016-011362. PubMed PMID: 27566632; PubMed Central PMCID: PMCPMC5013466.

25. Da Silva MC, Feijó BDM, Pereira FANS, Guerra FJF, Santos ISd, Rodrigues GDO, et al. Parto e nascimento na região rural: a violência obstétrica. *Revista de Enfermagem UFPE on line* (2018) 12(9):2407-17. doi: 10.5205/1981-8963-v12i9a234440p2407-2417-2018. PubMed PMID: 131728246. Language: English. Entry Date: 20180919. Revision Date: 20181010. Publication Type: Article. Journal Subset: Mexico & Central/South America.

26. Dey A, Shakya HB, Chandurkar D, Kumar S, Das AK, Anthony J, et al. Discordance in self-report and observation data on mistreatment of women by providers during childbirth in Uttar Pradesh, India. *Reproductive health* (2017) 14(1):149. Epub 2017/11/17. doi: 10.1186/s12978-017-0409-z. PubMed PMID: 29141640; PubMed Central PMCID: PMCPMC5688759.

27. Diamond-Smith N, Sudhinaraset M, Melo J, Murthy N. The relationship between women's experiences of mistreatment at facilities during childbirth, types of support received and person providing the support in Lucknow, India. *Midwifery* (2016) 40:114-23. Epub 2016/07/19. doi: 10.1016/j.midw.2016.06.014. PubMed PMID: 27428107.

28. Diamond-Smith N, Treleaven E, Murthy N, Sudhinaraset M. Women's empowerment and experiences of mistreatment during childbirth in facilities in Lucknow, India: results from a cross-sectional study. *BMC Pregnancy Childbirth* (2017) 17(Suppl 2):335. Epub 2017/11/17. doi: 10.1186/s12884-017-1501-7. PubMed PMID: 29143668; PubMed Central PMCID: PMCPMC5688442.

29. Pulerwitz J, Barker G. Measuring Attitudes toward Gender Norms among Young Men in Brazil. *Men and Masculinities* (2007) 10(3):322-38. doi: 10.1177/1097184x06298778.

30. Dynes MM, Twentyman E, Kelly L, Maro G, Msuya AA, Dominico S, et al. Patient and provider determinants for receipt of three dimensions of respectful maternity care in Kigoma Region, Tanzania-April-July, 2016. *Reproductive health* (2018) 15(1):41. Epub 2018/03/07. doi: 10.1186/s12978-018-0486-7. PubMed PMID: 29506559; PubMed Central PMCID: PMCPMC5838967.

31. Fair CD, Morrison TE. The relationship between prenatal control, expectations, experienced control, and birth satisfaction among primiparous women. *Midwifery* (2012) 28(1):39-44. Epub 2011/04/05. doi: 10.1016/j.midw.2010.10.013. PubMed PMID: 21458895.

32. Galle A, Manaharlal H, Cumbane E, Picardo J, Griffin S, Osman N, et al. Disrespect and abuse during facility-based childbirth in southern Mozambique: a cross-sectional study. *BMC pregnancy and childbirth* (2019) 19(1):369. Epub 2019/10/24. doi: 10.1186/s12884-019-2532-z. PubMed PMID: 31640603; PubMed Central PMCID: PMCPMC6805678.

33. Gashaye KT, Tsegaye AT, Shiferaw G, Worku AG, Abebe SM. Client satisfaction with existing labor and delivery care and associated factors among mothers who gave birth in university of Gondar teaching hospital; Northwest Ethiopia: Institution based cross-sectional study. *PloS one* (2019) 14(2):e0210693. Epub 2019/02/07. doi: 10.1371/journal.pone.0210693. PubMed PMID: 30726297; PubMed Central PMCID: PMCPMC6364872.

34. Gebremichael MW, Worku A, Medhanyie AA, Berhane Y. Mothers' experience of disrespect and abuse during maternity care in northern Ethiopia. *Global health action* (2018) 11(sup3):1465215. Epub 2018/06/05. doi: 10.1080/16549716.2018.1465215. PubMed PMID: 29860934; PubMed Central PMCID: PMCPMC5990935.

35. Gitobu CM, Gichangi PB, Mwanda WO. Satisfaction with Delivery Services Offered under the Free Maternal Healthcare Policy in Kenyan Public Health Facilities. *J Environ Public Health* (2018) 2018:4902864. Epub 2018/06/29. doi: 10.1155/2018/4902864. PubMed PMID: 29951103; PubMed Central PMCID: PMCPMC5987322.

36. Goncu Serhatlioglu S, Karahan N, Hollins Martin CJ, Martin CR. Construct and content validity of the Turkish Birth Satisfaction Scale - Revised (T-BSS-R). *J Reprod Infant Psychol* (2018) 36(3):235-45. Epub 2018/03/20. doi: 10.1080/02646838.2018.1443322. PubMed PMID: 29553295.

37. Gungor I, Beji NK. Development and psychometric testing of the scales for measuring maternal satisfaction in normal and caesarean birth. *Midwifery* (2012) 28(3):348-57. Epub 2011/05/07. doi: 10.1016/j.midw.2011.03.009. PubMed PMID: 21546142.

38. Haines HM, Hildingsson I, Pallant JF, Rubertsson C. The role of women's attitudinal profiles in satisfaction with the quality of their antenatal and intrapartum care. *J Obstet Gynecol Neonatal Nurs* (2013) 42(4):428-41. Epub 2013/06/19. doi: 10.1111/1552-6909.12221. PubMed PMID: 23773005.

39. Hameed W, Avan BI. Women's experiences of mistreatment during childbirth: A comparative view of home- and facility-based births in Pakistan. *PLoS One* (2018) 13(3):e0194601. Epub 2018/03/17. doi: 10.1371/journal.pone.0194601. PubMed PMID: 29547632; PubMed Central PMCID: PMCPMC5856402.

40. Heatley ML, Watson B, Gallois C, Miller YD. Women's Perceptions of Communication in Pregnancy and Childbirth: Influences on Participation and Satisfaction With Care. *J Health Commun* (2015) 20(7):827-34. Epub 2015/05/29. doi: 10.1080/10810730.2015.1018587. PubMed PMID: 26020149.

41. Hollins Martin CJ, Martin CR. Development and psychometric properties of the Birth Satisfaction Scale-Revised (BSS-R). *Midwifery* (2014) 30(6):610-9. Epub 2013/11/21. doi: 10.1016/j.midw.2013.10.006. PubMed PMID: 24252712.

42. Iida M, Horiuchi S, Porter SE. The relationship between women-centred care and women's birth experiences: a comparison between birth centres, clinics, and hospitals in Japan. *Midwifery* (2012) 28(4):398-405. Epub 2011/08/13. doi: 10.1016/j.midw.2011.07.002. PubMed PMID: 21835515.

43. Gokce IsbIr G, Inc IF, Bektas M, Dikmen Yildiz P, Ayers S. Risk factors associated with post-traumatic stress symptoms following childbirth in Turkey. *Midwifery* (2016) 41:96-103. Epub 2016/08/31. doi: 10.1016/j.midw.2016.07.016. PubMed PMID: 27571774.

44. Iyoke CA, Ezugwu FO, Ugwu GO, Lawani OL, Onyebuchi AK. Ethical aspects of obstetric care: expectations and experiences of patients in South East Nigeria. *Int J Womens Health* (2013) 5(1):571-82. Epub 2013/09/18. doi: 10.2147/IJWH.S49843. PubMed PMID: 24043956; PubMed Central PMCID: PMCPMC3772694.

45. Jha P, Larsson M, Christensson K, Skoog Svanberg A. Satisfaction with childbirth services provided in public health facilities: results from a cross- sectional survey among postnatal women in Chhattisgarh, India. *Global health action* (2017) 10(1):1386932. Epub 2017/11/01. doi: 10.1080/16549716.2017.1386932. PubMed PMID: 29087240; PubMed Central PMCID: PMCPMC5678347.

46. Johansson M, Hildingsson I. Intrapartum care could be improved according to Swedish fathers: mode of birth matters for satisfaction. *Women Birth* (2013) 26(3):195-201. Epub 2013/05/15. doi: 10.1016/j.wombi.2013.04.001. PubMed PMID: 23664433.

47. Kabakian-Khasholian T, Bashour H, El-Nemer A, Kharouf M, Sheikha S, El Lakany N, et al. Women's satisfaction and perception of control in childbirth in three Arab countries. *Reprod Health Matters* (2017) 25(sup1):16-26. Epub 2017/11/10. doi: 10.1080/09688080.2017.1381533. PubMed PMID: 29120285.

48. Kruk ME, Kujawski S, Mbaruku G, Ramsey K, Moyo W, Freedman LP. Disrespectful and abusive treatment during facility delivery in Tanzania: a facility and community survey. *Health policy and planning* (2018) 33(1):e26-e33. Epub 2018/01/06. doi: 10.1093/heapol/czu079. PubMed PMID: 29304252.

49. Kujawski S, Mbaruku G, Freedman LP, Ramsey K, Moyo W, Kruk ME. Association Between Disrespect and Abuse During Childbirth and Women's Confidence in Health Facilities in Tanzania. *Matern Child Health J* (2015) 19(10):2243-50. Epub 2015/05/21. doi: 10.1007/s10995-015-1743-9. PubMed PMID: 25990843.

50. Mehata S, Paudel YR, Dariang M, Aryal KK, Paudel S, Mehta R, et al. Factors determining satisfaction among facility-based maternity clients in Nepal. *BMC pregnancy and childbirth* (2017) 17(1):319. Epub 2017/09/28. doi: 10.1186/s12884-017-1532-0. PubMed PMID: 28946851; PubMed Central PMCID: PMCPMC5613378.

51. Meijer M, Brandao T, Canadas S, Falcon K. Components of obstetric violence in health facilities in Quito, Ecuador: A descriptive study on information, accompaniment, and position during childbirth. *International journal of gynaecology and obstetrics: the official organ of the International Federation of Gynaecology and Obstetrics* (2020) 148(3):355-60. Epub 2019/12/01. doi: 10.1002/ijgo.13075. PubMed PMID: 31785159.

52. Mihret MS. Obstetric violence and its associated factors among postnatal women in a Specialized Comprehensive Hospital, Amhara Region, Northwest Ethiopia. *BMC Res Notes* (2019) 12(1):600. Epub 2019/09/20. doi: 10.1186/s13104-019-4614-4. PubMed PMID: 31533858; PubMed Central PMCID: PMCPMC6751597.

53. Monazea EM, Al-Attar GS. Quality of delivery care in Assiut University Hospital, Egypt: mothers' satisfaction. *The Journal of the Egyptian Public Health Association* (2015) 90(2):64-71. Epub 2015/07/15. doi: 10.1097/01.EPX.0000466380.29269.4b. PubMed PMID: 26154833.

54. Montesinos-Segura R, Urrunaga-Pastor D, Mendoza-Chuctaya G, Taype-Rondan A, Helguero-Santin LM, Martinez-Ninanqui FW, et al. Disrespect and abuse during childbirth in fourteen hospitals in nine cities of Peru. *International journal of gynaecology and obstetrics: the official organ of the International Federation of Gynaecology and Obstetrics* (2018) 140(2):184-90. Epub 2017/10/19. doi: 10.1002/ijgo.12353. PubMed PMID: 29044510.

55. Montoya A, Fritz J, Labora A, Rodriguez M, Walker D, Trevino-Siller S, et al. Respectful and evidence-based birth care in Mexico (or lack thereof): An observational study. *Women Birth* (2020) 33(6):574-82. Epub 2020/03/01. doi: 10.1016/j.wombi.2020.02.011. PubMed PMID: 32111555.

56. Morton CH, Henley MM, Seacrist M, Roth LM. Bearing witness: United States and Canadian maternity support workers' observations of disrespectful care in childbirth. *Birth* (2018) 45(3):263-74. Epub 2018/07/31. doi: 10.1111/birt.12373. PubMed PMID: 30058157.

57. Mukamurigo JU, Berg M, Ntaganira J, Nyirazinyoye L, Dencker A. Associations between perceptions of care and women's childbirth experience: a population-based cross-sectional study in Rwanda. *BMC Pregnancy Childbirth* (2017) 17(1):181. Epub 2017/06/11. doi: 10.1186/s12884-017-1363-z. PubMed PMID: 28599645; PubMed Central PMCID: PMCPMC5466750.

58. Okafor, II, Ugwu EO, Obi SN. Disrespect and abuse during facility-based childbirth in a low-income country. *International journal of gynaecology and obstetrics: the official organ of the International Federation of Gynaecology and Obstetrics* (2015) 128(2):110-3. Epub 2014/12/06. doi: 10.1016/j.ijgo.2014.08.015. PubMed PMID: 25476154.

59. Okumus F. Birth experiences of primiparous Turkish women: public and private hospitals. *Journal of Asian Midwives* (2017) 4(1):35-46. PubMed PMID: 124425275. Language: English. Entry Date: 20180117. Revision Date: 20190304. Publication Type: Article.

60. Overgaard C, Fenger-Gron M, Sandall J. The impact of birthplace on women's birth experiences and perceptions of care. *Soc Sci Med* (2012) 74(7):973-81. Epub 2012/02/14. doi: 10.1016/j.socscimed.2011.12.023. PubMed PMID: 22326105.

61. Redshaw M, Martin CR, Savage-McGlynn E, Harrison S. Women's experiences of maternity care in England: preliminary development of a standard measure. *BMC pregnancy and childbirth* (2019) 19(1):167. Epub 2019/05/16. doi: 10.1186/s12884-019-2284-9. PubMed PMID: 31088487; PubMed Central PMCID: PMCPMC6518811.

62. Rosen HE, Lynam PF, Carr C, Reis V, Ricca J, Bazant ES, et al. Direct observation of respectful maternity care in five countries: a cross-sectional study of health facilities in East and Southern Africa. *BMC pregnancy and childbirth* (2015) 15(1):306. Epub 2015/11/26. doi: 10.1186/s12884-015-0728-4. PubMed PMID: 26596353; PubMed Central PMCID: PMCPMC4657214.

63. Sando D, Ratcliffe H, McDonald K, Spiegelman D, Lyatuu G, Mwanyika-Sando M, et al. The prevalence of disrespect and abuse during facility-based childbirth in urban Tanzania. *BMC pregnancy and childbirth* (2016) 16:236. Epub 2016/08/21. doi: 10.1186/s12884-016-1019-4. PubMed PMID: 27543002; PubMed Central PMCID: PMCPMC4992239.

64. Sethi R, Gupta S, Oseni L, Mtimuni A, Rashidi T, Kachale F. The prevalence of disrespect and abuse during facility-based maternity care in Malawi: evidence from direct observations of labor and delivery. *Reproductive health* (2017) 14(1):111. Epub 2017/09/08. doi: 10.1186/s12978-017-0370-x. PubMed PMID: 28877701; PubMed Central PMCID: PMCPMC5588731.

65. Sheferaw ED, Bazant E, Gibson H, Fenta HB, Ayalew F, Belay TB, et al. Respectful maternity care in Ethiopian public health facilities. *Reproductive health* (2017) 14(1):60. Epub 2017/05/18. doi: 10.1186/s12978-017-0323-4. PubMed PMID: 28511685; PubMed Central PMCID: PMCPMC5434569.

66. Sheferaw ED, Kim YM, van den Akker T, Stekelenburg J. Mistreatment of women in public health facilities of Ethiopia. *Reproductive health* (2019) 16(1):130. Epub 2019/08/29. doi: 10.1186/s12978-019-0781-y. PubMed PMID: 31455400; PubMed Central PMCID: PMCPMC6712647.

67. Silveira MF, Mesenburg MA, Bertoldi AD, De Mola CL, Bassani DG, Domingues MR, et al. The association between disrespect and abuse of women during childbirth and postpartum depression: Findings from the 2015 Pelotas birth cohort study. *J Affect Disord* (2019) 256:441-7. Epub 2019/06/30. doi: 10.1016/j.jad.2019.06.016. PubMed PMID: 31252237; PubMed Central PMCID: PMCPMC6880287.

68. Siraj A, Teka W, Hebo H. Prevalence of disrespect and abuse during facility based child birth and associated factors, Jimma University Medical Center, Southwest Ethiopia. *BMC pregnancy and childbirth* (2019) 19(1):185. Epub 2019/05/28. doi: 10.1186/s12884-019-2332-5. PubMed PMID: 31132988; PubMed Central PMCID: PMCPMC6537397.

69. Sjetne IS, Iversen HH, Kjollesdal JG. A questionnaire to measure women's experiences with pregnancy, birth and postnatal care: instrument development and assessment following a national survey in Norway. *BMC pregnancy and childbirth* (2015) 15:182. Epub 2015/08/22. doi: 10.1186/s12884-015-0611-3. PubMed PMID: 26294064; PubMed Central PMCID: PMCPMC4546178.

70. Souza KJ, Rattner D, Gubert MB. Institutional violence and quality of service in obstetrics are associated with postpartum depression. *Revista de saude publica* (2017) 51:69. Epub 2017/07/27. doi: 10.1590/S1518-8787.2017051006549. PubMed PMID: 28746574; PubMed Central PMCID: PMCPMC5510781.

71. Szebik I, Susanszky E, Szanto Z, Susanszky A, Rubashkin N. ETHICAL IMPLICATIONS OF OBSTETRIC CARE IN HUNGARY: Results from the Mother-Centred Pregnancy Care Survey. *European Journal of Mental Health* (2018) 13(1):51-69. doi: 10.5708/Ejmh.13.2018.1.5. PubMed PMID: WOS:000435209400005.

72. Taavoni S, Goldani Z, Rostami Gooran N, Haghani H. Development and Assessment of Respectful Maternity Care Questionnaire in Iran. *Int J Community Based Nurs Midwifery* (2018) 6(4):334-49. Epub 2018/11/23. PubMed PMID: 30465006; PubMed Central PMCID: PMCPMC6226608.

73. Tekle Bobo F, Kebebe Kasaye H, Etana B, Woldie M, Feyissa TR. Disrespect and abuse during childbirth in Western Ethiopia: Should women continue to tolerate? *PloS one* (2019) 14(6):e0217126. Epub 2019/06/08. doi: 10.1371/journal.pone.0217126. PubMed PMID: 31173588; PubMed Central PMCID: PMCPMC6555589.

74. Thies-Lagergren L, Johansson M. Intrapartum midwifery care impact Swedish couple's birth experiences - A cross-sectional study. *Women Birth* (2019) 32(3):213-20. Epub 2018/09/16. doi: 10.1016/j.wombi.2018.08.163. PubMed PMID: 30217554.

75. Ukke GG, Gurara MK, Boynito WG. Disrespect and abuse of women during childbirth in public health facilities in Arba Minch town, south Ethiopia - a cross-sectional study. *PloS one* (2019) 14(4):e0205545. Epub 2019/04/30. doi: 10.1371/journal.pone.0205545. PubMed PMID: 31034534; PubMed Central PMCID: PMCPMC6488058.

76. van der Kooy J, Valentine NB, Birnie E, Vujkovic M, de Graaf JP, Denktas S, et al. Validity of a questionnaire measuring the world health organization concept of health system responsiveness with respect to perinatal services in the Dutch obstetric care system. *BMC health services research* (2014) 14:622. Epub 2014/12/04. doi: 10.1186/s12913-014-0622-1. PubMed PMID: 25465053; PubMed Central PMCID: PMCPMC4265356.

77. van der Kooy J, Birnie E, Valentine NB, de Graaf JP, Denktas S, Steegers EAP, et al. Quality of perinatal care services from the user's perspective: a Dutch study applies the World Health Organization's responsiveness concept. *BMC pregnancy and childbirth* (2017) 17(1):327. Epub 2017/10/01. doi: 10.1186/s12884-017-1464-8. PubMed PMID: 28962611; PubMed Central PMCID: PMCPMC5622418.

78. Vardavaki Z, Martin CJH, Martin CR. Construct and content validity of the Greek version of the Birth Satisfaction Scale (G-BSS). *Journal of Reproductive and Infant Psychology* (2015) 33(5):488-503. doi: 10.1080/02646838.2015.1035235. PubMed PMID: WOS:000361965500005.

79. Vedam S, Stoll K, Taiwo TK, Rubashkin N, Cheyney M, Strauss N, et al. The Giving Voice to Mothers study: inequity and mistreatment during pregnancy and childbirth in the United States. *Reproductive health* (2019) 16(1):77. Epub 2019/06/12. doi: 10.1186/s12978-019-0729-2. PubMed PMID: 31182118; PubMed Central PMCID: PMCPMC6558766.

80. Vedam S, Stoll K, Rubashkin N, Martin K, Miller-Vedam Z, Hayes-Klein H, et al. The Mothers on Respect (MOR) index: measuring quality, safety, and human rights in childbirth. *SSM Popul Health* (2017) 3:201-10. Epub 2018/01/20. doi: 10.1016/j.ssmph.2017.01.005. PubMed PMID: 29349217; PubMed Central PMCID: PMCPMC5768993.

81. Wassihun B, Deribe L, Worede N, Gultie T. Prevalence of disrespect and abuse of women during child birth and associated factors in Bahir Dar town, Ethiopia. *Epidemiology and health* (2018) 40:e2018029. Epub 2018/07/31. doi: 10.4178/epih.e2018029. PubMed PMID: 30056644; PubMed Central PMCID: PMCPMC6178351.

82. Wassihun B, Zeleke S. Compassionate and respectful maternity care during facility based child birth and women's intent to use maternity service in Bahir Dar, Ethiopia. *BMC Pregnancy Childbirth* (2018) 18(1):294. Epub 2018/07/11. doi: 10.1186/s12884-018-1909-8. PubMed PMID: 29986659; PubMed Central PMCID: PMCPMC6038196.
