# Supplementary material for: A critical review: developing a birth integrity framework for epidemiological studies through meta-ethnography
Source: BMC Womens Health. 2023 Oct 10;23:530. doi: 10.1186/s12905-023-02670-z (PMC10565979; doi:10.1186/s12905-023-02670-z)
Supplement: Supplementary file 5 — Additional file 5: Process of identifying key concepts in conceptual clusters. Table S1. Disrespect and abuse (D&A), Mistreatment during facility-based childbirth (MisC). Table S2. Respectful maternity care (RMC). Table S3. Childbirth experiences (CE). Table S4. Maternal satisfaction (MS). Table S5. Obstetric violence. Table S6. Person-centered care (PCC). [file 12905_2023_2670_MOESM5_ESM.docx]

**Additional file 5: Process of identifying key concepts in conceptual clusters**

[**Table S1: Disrespect and abuse (D&A), Mistreatment during facility-based childbirth (MisC)** 1](#_Toc128735869)

[**Table S2: Respectful maternity care (RMC)** 3](#_Toc128735870)

[**Table S3: Childbirth experiences (CE)** 4](#_Toc128735871)

[**Table S4: Maternal satisfaction (MS)** 5](#_Toc128735872)

[**Table S5: Obstetric violence** 7](#_Toc128735873)

[**Table S6: Person-centered care (PCC)** 8](#_Toc128735874)

**Table S1: Disrespect and abuse (D&A), Mistreatment during facility-based childbirth (MisC)**

| **Key concept derived from conceptual ideas:** Disrespect and abuse reflect any form of inhumane treatment or uncaring behavior towards a woman during labor and birth. D&A represents a fundamental violation of women’s human rights and undermines the safety and effectiveness of health systems, e.g., through non-dignified care, non-consented care, neglect or abandonment, or lack of privacy. Mistreatment (MisC) in childbirth describes childbirth-related mistreatment at an interpersonal but also at the health-system level and comprises seven domains: 1. physical abuse, 2. sexual abuse, 3. verbal abuse, 4. stigma and discrimination, 5. failure to meet professional standards, 6. poor rapport between women and providers 7. health system conditions and constraints. Drivers of D&A/MisC can include systemic failures, such as overwhelmed health care administration, poor staffing, and inadequate infrastructure. | |
| --- | --- |
| **Study** | **Conceptual idea (individual studies in D&A and MisC cluster)** |
| **(1)** | Women experience ill treatment not only in violation of their autonomy and dignity but also as verbal insults, humiliation, discrimination, abandonment of care and physical assault during childbirth. (…) formally called these maltreatments Disrespect and Abuse (D&A) during childbirth and highlighted this as a main factor in the underutilization of health care facilities. Although an objective assessor reviewing statements about a woman’s experience during labor and birth may see that she has been a victim of D & A “experienced D & A”, the woman herself may not recognize that this was D & A “reported D & A. |
| **(2)** | N/A |
| **(3)** | “Bowser and Hill conducted a land- scape analysis identifying seven categories of disrespect and abuse: physical abuse, non-consented care, non-confidential care, non-dignified care, discrimination based on specific patient attributes, abandonment of care, and detention in facilities due to failure to pay (…)“. |
| **(4)** | “Disrespectful and abusive care includes impoliteness of care providers, inappropriate reprimands, shouting at the client, lack of empathy, refusal to assist, threatening clients for their non-compliance, and denying clients opportunities to choose or give an opinion on the care they are receiving”. |
| **(5)** | “These include physical abuse (beating, slapping and pinching), lack of consent for care (e.g. for Caesarean section or tubal ligation), non- confidential care (e.g. lack of physical privacy or sharing of confidential information), undignified care (e.g. shouting, scolding and demeaning comments), abandonment (e.g. being left alone during delivery), discrimination on the basis of ethnicity, age, or wealth, or detention in facilities for failure to pay user fees" following the Browser and Hill categorization (…)”. |
| **(6)** | “Disrespectful and abusive treatment during childbirth, such as physical abuse, abandonment, threatening and negative language, shouting and scolding, physical privacy violations, and non-consented care, has been observed in several resource-constrained contexts, including Tanzania (…)”. |
| **(7)** | “D&A is also a fundamental violation of women’s human rights and undermines the safety and effectiveness of health systems. (…) seven categories of disrespect and abuse (D&A) during childbirth emerged from qualitative and anecdotal reports: physical abuse, non-consented care, non-confidential care, non- dignified care, discrimination, abandonment, and detention in health care facilities (…)”. |
| **(8)** | “Disrespect and abuse are defined as any form of inhumane treatment or uncaring behavior toward a woman during labor and delivery (…). Laboring mothers may face various forms of disrespectful and abusive treatment during childbirth at a facility, including physical abuse, lack of consent for care, non-confidential care, undignified care, abandonment, discrimination, and detention in facilities for failure to pay user fees” (…). |
| **(9)** | “D&A has been acknowledged as a deficiency in the delivery of high quality maternal health services, threatening the ability of health systems to achieve good maternal health outcomes (…). D&A manifests as physical violence, harsh language, stigma and neglect suffered by women at the hands of health care providers (…). Drivers of D&A can include systemic failures, such as overwhelmed health care administration, poor staffing and supervisory structures and inadequate physical infrastructure (…). Women who experience D&A are more likely to report lower satisfaction with their birth experience and are less likely to seek facility-based delivery for future pregnancies (…)”. |
| **(10)** | “Disrespect and abuse during childbirth care is considered a form of violence that directly violates women’s rights as defined by the United Nations—i.e., the right to respect, timely care, autonomy, self-determination, and information during childbirth (…)”. |
| **(11)** | “ (…) 7 categories of disrespect and abuse during childbirth: physical abuse, non-consented care, non-confidential care, non-dignified care, discrimination based on specific patient attributes, abandonment of care, and detention in facilities (…)”. |
| **(12)** | “(…) physical abuse; non-consented care; non-confidential care; non-dignified care; discrimination; abandonment of care; and detention in facilities (…).The mistreatment of women during childbirth often occurs at the level of the interaction between women and healthcare providers but deficiencies in the health care system (e.g. lack of adequate personal and poor infrastructure) also contribute to its occurrence (…)”. |
| **(13)** | “Disrespect and abuse during childbirth is common throughout the world (…). It can occur at the level of contact between the client and the care provider, as well as through systemic failures at the health facility and health system level (…)“. |
| **(14)** | „While disrespect and abuse during delivery does not necessarily mean that respectful care was provided, it does mean that the fundamental human right of women to receive the highest attainable standard of care was violated (…)”. |
| **(15)** | “Three typologies of disrespectful care: 1. verbal abuse including threats of poor outcome, racially demeaning comments; sexually degreeding remarks. 2.stigma and discrimination: extra procedures because of race/ethnicity. 3. failure to meet professional standards of care: failure to secure fully informed consent or performing procedures explicitly against a women´s wishes (…)”. |
| **(16)** | „(…) domains of mistreatment, including physical, sexual, and verbal abuse, stigma and discrimination, failure to meet professional standards of care, poor rapport between women and providers, and health care-related conditions and constraints“. |
| **(17)** | „This mistreatment can have immediate and long-term consequences: for example, denial of pain relief medication, episiotomy (without anaesthesia) and physical abuse can cause extreme pain and suffering (…) It may also lead to adverse psychological effects such as re-traumatisation (…) post-traumatic stress symptoms, sleeping problems, poor self-rated health (…) and feelings of dehumanisation (…) that could result in a distorted body perception and fear of childbirth (…).These categories were reworked as follows: physical abuse, verbal abuse, right to information, non-consented care, non-confidential care, discrimination and abandonment of care.” |
| **(18)** | “Mistreatment can encompass a number of factors, including verbal and physical abuse, disrespect, and neglect of various forms (…)”. |
| **(19)** | “(…) ‘mistreatment’ and delineated the phenomena across seven dimensions: physical abuse, sexual abuse, verbal abuse, stigma and discrimination, failure to meet professional standards of care, poor rapport between women and providers, and poor conditions and constraints presented by the health system (…)”. |
| **(20)** | “Evidence suggests that women across the world experience mistreatment during childbirth, including physical abuse, verbal abuse, discrimination, non-consented procedures, and non-supportive care (…). Bowser and Hill’s landscape analysis (…) brought this issue to global attention and our mixed-methods systematic review developed a typology of what constitutes mistreatment”. |
| **(21)** | “Physical abuse included hitting, slapping or pinching. Verbal abuse included shouting, scolding, threatening to take women into the operating theatre or addressing women using insulting names. Failure to meet standards of care included neglecting women when they needed care at some point during labor and childbirth, ignoring women’s requests for pain relief, providing treatment without consent and providing care that violated privacy of women. Poor rapport between women and providers included not greeting women, not explaining the labor progress, not responding to women’s questions in a polite manner, not encouraging women to move around freely, not allowing women to bring a companion, not allowing women to give birth in their preferred birth position and not offering hot drinks or food after childbirth“(…)”. |
| **(22)** | „A growing body of literature suggests that fear of such mistreatment is a key impediment to timely acquisition of care and use of institutional facilities for childbirth, particularly among less educated and poor women, and is associated with poor birth outcomes for both mother and child (…) Such mistreatment can include a broad array of provide behaviors, from neglectful or non-consensual care to verbal or physical abuse against a woman during childbirth (…)”. |
| **(23)** | “Seven categories of disrespect and abuse during child- birth are physical abuse, non-dignified care, discrimination based on specific patient attributes, non-consented care, non-confidential care, abandonment of care and detention in facilities. However, Numerous factors (individual and community-level) may contribute to the experiences of disrespect and abuse. Lack of legal and ethical foundations to address D&A, normalizing D&A, lack of standards and accountability, lack of leadership commitment, and provider prejudice due to training and lack of resources are some among many factors (…)”. |
| **(24)** | “(…) seven categories of attributes that effectively defined disrespectful and abusive care in facility-based skilled childbirth: physical abuse, non-consented care, non-confidential care, non-dignified care, discrimination, abandonment/ neglect of care, and detention in facilities until hospital bills are paid (…)“. |
| **(25)** | “Women ́s experiences of disrespect and abuse often results from the nature of patient-provider interactions in the context of obstetric care and can be expressed as verbal, physical or sexual abuse, stigma and discrimination, neglect, and failure to meet standards of care and attention – such as privacy and confidentiality breaches, limiting access to information and medical procedures con- ducted without consent (…). They have also been linked to the institutional structures and processes that frame the practice of obstetric care in health systems and the persistence of structural gender inequalities in society being considered by some authors as a dimension of violence against women (…)”. |
| **(26)** | “(…) Types of D&A were then categorized as physical abuse, non-dignified care, abandonment, non-consented care, non-confidential care, detention and discrimination.” |
| **(27)** | „Measures of mistreatment for this assessment were collected during the birthing process and categorised using the WHO typology at the analysis (…) stage.  For example, three second order themes were assessed during admission: harsh language, lack of informed consent and lack of privacy. During delivery, four second order themes were examined: harsh language, use of force, unhygienic conditions (these were defined as the basic requirement a provider must adhere to as part of broader infection control practices regard- less of level of care), and lack of privacy. During immediate postpartum care, three second order themes were assessed: unhygienic conditions, lack of privacy and lack of informed consent”. |
| **(28)** | “Mistreatment comprises (…) seven domains: 1. physical abuse, such as slapping, 2. sexual abuse, such as rape, 3. verbal abuse, such as shouting, 4. stigma and discrimination, such as providing poor treatment due to HIV status, 5. failure to meet professional standards, such as neglect, 6. poor rapport between women and providers, such as dis- missal of women’s concerns, and 7. health system conditions and constraints, such as lack of privacy. Mistreatment is often justified as a means of punishment for patients’ misbehavior (…)“. |
| **(29)** | “A systematic review in the area of negligence and violations of childbirth led by Bohren et al. allowed a widening to the typology of these abuses (…). The review presented a detailed typology that was evidence based and comprehensively illustrated how women in perinatal care facilities can be mistreated on multiple levels: inter- actions between women and healthcare providers as well as system and organizational failures (…)“. |
| **(30)** | “(…) interactions or facility conditions that local consensus deems to be humiliating or undignified, and those interactions or conditions that are experienced as or intended to be humiliating or undignified (…)” |

**Table S2: Respectful maternity care (RMC)**

| **Key concept derived from conceptual ideas:** A universal human right that is due to every childbearing woman in every health system around the world in which the maternity care is expanded beyond the prevention of morbidity or mortality to encompass respect for women’s basic human rights. Components of RMC are: Freedom from harm and ill treatment; Right to information, informed consent and refusal, and respect for choices and preferences, including the right to companionship of choice whenever possible; Confidentiality, privacy; Dignity, respect; Equality, freedom from discrimination, equitable care; Right to timely health care and to the highest attainable level of health; and Liberty, autonomy, self-determination, and freedom from coercion. | |
| --- | --- |
| **Study** | **Conceptual idea (individual studies in RMC cluster)** |
| **(31)** | „Seven rights of childbearing women from Respectful Maternity Care Charter (…) Article 1. Every woman has the right to be free from harm and ill treatment., Article 2. Every woman has the right to information, informed consent and refusal, and respect for her choices and preferences, including companionship during maternity care. Article 3. Every woman has the right to privacy and confidentiality. Article 4. Every woman has the right to be treated with dignity and respect. Article 5. Every woman has the right to equality, freedom from discrimination, and equitable care. Article 6. Every woman has the right to healthcare and to the highest attainable level of health. Article 7. Every woman has the right to liberty, autonomy, self-determination, and freedom from coercion”. |
| **(32)** | “A universal human right that is due to every childbearing woman in every health system around the world in which the maternity care is expanded beyond the prevention of morbidity or mortality to encompass respect for women’s basic human rights, including respect for women’s autonomy, dignity, feelings, choices, and preferences, such as having a companion wherever possible (…)”. |
| **(33)** | “Respectful Maternity Care Charter defined seven rights of childbearing women(…): Freedom from harm and ill treatment; Right to information, informed consent and refusal, and respect for choices and preferences, including the right to companionship of choice whenever possible; Confidentiality, privacy; Dignity, respect; Equality, freedom from discrimination, equitable care; Right to timely health care and to the highest attainable level of health; and Liberty, autonomy, self-determination, and freedom from coercion.”. |
| **(34)** | “Respectful maternity care (RMC) during childbirth is an interaction between the client and the healthcare providers (HCPs) or facility conditions. It has a significant role in maternal mortality ratio reduction by enhancing clients’ inclination to deliver in health facilities. Furthermore, RMC is the standard of care for all women that encompasses women’s basic human rights”. |
| **(35)** | “Respectful maternity care encompasses physical and psychological care, communication and interactions, is influenced by structural, organisational and cultural systems, and financial issues and implies 'doing no harm'. The terms used to describe respectful care include both positive descriptions, such as ‘respectful’ and ‘humanised’, and negative descriptions, such as ‘disrespectful’, ‘obstetric violence’, ‘mistreatment’ and ‘abuse’”. |
| **(36)** | “The White Ribbon Alliance defines RMC as an approach that emphasizes the positive inter- personal interactions of women with health care providers and staff during labor, delivery, and the postpartum period. Absence of D&A by health care providers and other staff alone is not sufficient for provision of RMC; the RMC definition calls for fostering positive staff attitudes and behaviors that are conducive to improved satisfaction of women with their birth experience”. |
| **(37)** | “Over recent years, promotion of the usage of Respectful Maternity Care (RMC) has been developed gradually, emphasizing the importance of underlying professional ethics and considering psychological, social and cultural aspects of health care delivery as essential elements of care (…). While medical treatment is only one aspect of RMC, failure to focus on the well-being of women and newborns by imposing unnecessary or harmful practices can be considered abusive and disrespectful (…)”. |

**Table S3: Childbirth experiences (CE)**

| **Key concept derived from conceptual ideas:** Childbirth experiences and especially a woman’s relationship with her health care providers in maternity settings significantly impact her health. It has long-term implications for her future emotional, physical, and reproductive health and wellbeing. Negative CE increases the risk for postpartum depression, secondary fear of childbirth, and post-traumatic stress disorder. | |
| --- | --- |
| **Study** | **Conceptual idea (individual studies in CE cluster)** |
| **(38)** | “A positive childbirth experience is important for the woman’s wellbeing, facilitates the mother-child bonding and may have implications for the future health for both the mother and baby. On the contrary, a negative experience increases the risk for postpartum depression, secondary fear of childbirth and post-traumatic stress disorder”. |
| **(39)** | “A woman’s experience of labour and birth may have long-lasting and profound effects on her wellbeing and that of her baby and husband.2 Further, the childbirth experiences of primiparous women are especially important because of their impact on future births, most especially if the first birth is a caesarean section. There is also an impact on the nature of the birth stories that are told to subsequent generations. Negative childbirth experiences often lead women to prefer caesarean sections to vaginal birth (…)”. |
| **(40)** | “Women's relationship with health care providers in maternity settings during childbirth significantly impacts their physical, psychological, and emotional health during childbirth. (…) An important, but little understood component of the poor quality of care experienced by women during childbirth in facilities is disrespectful and abusive behavior by health care professionals and other facility staff”. |
| **(41)** | “Childbirth is a highly significant event for the mother, her family and the community (…) The experience of giving birth has long-term implications for mothers’ future emotional, physical and reproductive health and wellbeing.3,4 It has been shown that the experience of childbirth also has an impact on the birth partner’s future emotional, physical and reproductive health and wellbeing. Parents’ birth experiences affect bonding with their child which in turn may influence the child’s future health. (…) Quality of intrapartum care is understood as a resource structure of the care organisation in combination with parents’ preferences and therefore it is important to not only measure satisfaction but also to simultaneously measure the subjective importance accorded the care given”. |
| **(42)** | “While positive birth experiences contribute to women’s feeling of accomplishment and self-esteem and lead to psychological growth, empowerment, and easier adaptation to motherhood (…), negative experiences are associated with a number of complications such as postpartum anxiety, depression, post- traumatic stress syndrome (…) fear of childbirth (…), reduced future reproduction (…),and request for caesarean section (…) Four key dimensions of patient-centred care (…)have been identified as prominent aspects of the childbirth experience: the woman’s perceptions of intrapartum support, participation in decision-making, information, and control“. |
| **(43)** | “Positive experiences during this time can be looked back upon fondly, empowering the woman in her role as a mother, and strengthening her emotionally during her transition to motherhood (…) Conversely, a negative maternity experience may significantly increase the risk of negative health outcomes for the mother such as postnatal mental health disorders with possible long-lasting effects on the mother, the child, and the family system as a whole. The care a woman receives during the perinatal period can have a profound impact on her overall maternity experience, with potentially significant implications for her health and wellbeing both at the time and subsequently (…) In turn, this can impact on the mother-baby relationship and also on the health and wellbeing of the baby (…).A woman’s experiences and memories of maternity care might also influence her decision-making regarding future pregnancies, requests for medical intervention during future childbirth, as well as having an im- pact on future reproduction in general. Thus, it is necessary to monitor, evaluate and optimise the care that women and their families receive during this important time”. |
| **(44)** | “Collection of patient-reported outcomes, including patient experiences, is an important aspect of evaluations of health services. (…) These surveys call for descriptions of mainly non- technical aspects of the health-care services and may in- volve different target populations, such as the general population, broad groups of service users, or patients with specific conditions (…)”. |

**Table S4: Maternal satisfaction (MS)**

| **Key concept derived from conceptual ideas:** Maternal satisfaction refers to a woman’s subjective and dynamic evaluation of her birth experience. This multifaceted construct includes elements of perceived quality of care, coping efficacy, and reflections of the birth experience as a whole and in context. Low MS can affect the mother’s and infant’s health. Low levels of MS are associated with greater odds of postnatal depression, post-traumatic stress disorder, requests for future elective cesarean section, sterilization, and abortion. | |
| --- | --- |
| **Study** | **Conceptual idea (individual studies in MS cluster)** |
| **(45)** | “An important predictor of satisfaction is quality of care (contributors to poor quality of care (…): provider incompetency, lack of drugs and supplies, delay in referral, non cleanliness, and poor interaction between clients and healthcare providers (…). Women are more vocal about patient–provider communication and value good interaction with their provider (…). Mothers who are treated with respect, courtesy, and dignity are more likely to be satisfied with the obstetric care (…). The influences of the attitudes and behaviors of the caregivers are more powerful and obvious on subsequent satisfaction than the influences of pain relief, and intrapartum medical interventions (…) even with the evidence that the majority of women would want pain relief in labor (…). Moreover, it was concluded that poor sanitary condition of the health facilities and lack of basic amenities were the major cause of dissatisfaction (…)”. |
| **(46)** | “A woman’s satisfaction with the birth experience has been shown to influence her relationship with her infant, to affect her self-esteem and self-image, and influence her future childbirth expectations. Perceptions of being in control during childbirth have been recognised as the strongest component of women’s birth experiences, of their own behaviour during labour and their inter- action with care providers, contributing largely to women’s feelings of fulfilment and postpartum well-being. Satisfaction is also related to the caregiver’s attitude, good communication with care providers, and the responsiveness of staff to women’s needs. One report indicates that dissatisfaction with care and perceptions of diminished control over the process of childbirth have led to a preference for caesarean sections for future births”. |
| **(47)** | “Studies show that women who are satisfied with child- birth services tend to have better self-esteem and confidence, are faster in establishing a maternal–neonatal bond, and are more likely to breastfeed compared with women who are dissatisfied (…). Women who are dis- satisfied with their childbirth experiences are more prone to develop a fear of childbirth and postnatal depressive symptoms, and to face difficulties in breastfeeding and in performing baby and self-care (…)”. |
| **(48)** | “Women’s satisfaction with their childbirth experience also has implications for the health and well-being of a woman and her newborn. A woman’s satisfaction with her childbirth experience may have immediate and long-term effects on her health and her relationship with her infant, including: postpartum depression, post-traumatic stress disorder, future abortions, a lack of ability to resume sexual intercourse, preference for a caesarean section, negative feelings towards her infant, poor adaptation to the mothering role and breast-feeding problems (…)”. |
| **(49)** | “Every woman's perceptions of birth are important, which within this study is conceptualised as ‘birth satisfaction’. In terms of quantitative research, a woman's satisfaction with intrapartum care can only be considered high quality when gratification over what she received is measured as high (…).” |
| **(50)** | “Patients’ satisfaction with healthcare services is one of the measures for quality of care that has been shown to influence confidence in a health facility and the subsequent utilization of services from the facility (…). Patients’ satisfaction with quality of healthcare is the degree to which the patients’ desired expectations, goals, and preferences are provided by the healthcare service providers (…). Patients’ satisfaction and dissatisfaction with healthcare services indicate their perception about the strengths and weaknesses in the service delivery (…)”. |
| **(51)** | “The outcomes of health care delivery are measured in terms of effectiveness and efficiency but also in terms of the individual’s experience as a patient. This experience involves pain, autonomy, a feeling of physical and mental well-being and satisfaction with the favorable results achieved (...) and provides a unique opportunity to better understand satisfaction with the quality of the health care provided (…). Satisfaction with health care delivery is significantly associated with patients’ adherence to medical treatment (…), their quality of life) (…) or simply improvements in their health status (…). Therefore, patients’ experiences are increasingly being used inter- nationally as an indicator of the quality and performance of health systems (…), and thousands of surveys are used by health care providers, administrators or policymakers to assess the quality of care, make decisions about pro- visions and organization of health care services, avoid malpractice and support a competitive edge in the health care area (…)”. |
| **(52)** | “Patient satisfaction is a subjective and dynamic perception of the extent to which the expected health care is received (...). It is not important whether the patient is right or wrong, but what is important is how the patient feels”. |
| **(53)** | “Satisfaction, patient perceptions, and actual experiences of the care received are not synonymous concepts, although the terms are often used inter- changeably within one study and between studies (…) Most women report high satisfaction with maternity care, but when asked to consider particular aspects of that care they are more critical. During the intrapartum period, women are consistently dissatisfied with three dimensions of care: their perceived sense of control (…), support received from caregivers (…), and their experiences of managing pain (…). Dissatisfaction has been reported to be associated with operative delivery (especially emergency caesarean) and admission of the infant to neonatal intensive care (…)”. |
| **(54)** | “Satisfaction with maternity care is a multidimensional construct embracing satisfaction with self (personal control), and with the physical environment of delivery room and quality of care. Aspects of care that may influence client satisfaction include provider attitude, provider competence, outcome, physical environment, continuity of care, access, information, cost, bureaucracy and attention to psychosocial problems. Quality of care may not al- ways be linearly associated with the level of satisfaction as perceived by the clients; however client satisfaction an important determinant of utilization of health services and the choice of health facility (…). Women who are treated with respect, courtesy and dignity, and have trusting relationships with their care providers are more likely to be satisfied (…). Lack of involvement in decision making and inadequate information about their care are associated with dissatisfaction (…)”. |
| **(55)** | “Satisfaction with childbirth is the most important qualitative outcome in assessing childbirth experiences, given the fact that this experience affects their health and tehri relationship with their infant. (…) Five dimensions: the delivery experience (pain intensity, complications and length of labour), medical care, nursing care, information received and participation in the decision-making process, and physical aspects of the labour and delivery rooms. (…) identified the following features of obstetric care as influencing satisfaction with childbirth: explanation of procedures and involvement of mothers in administering or choosing them; support from the presence of a partner and qualified hospital staff; and physical comfort of the postnatal ward. (…) described factors contributing to a satisfying birth experience as follows: support, information, intervention, decision-making, control, pain relief and trial participation (…)”. |
| **(56)** | “Birth satisfaction refers to a woman’s satisfaction with her birth experience throughout labour, birth, and the immediate postpartum period (…). It is assessed by measuring the mother’s perceptions of care received, maternal control, personal support, medical interventions, and overall health (…) Birth satisfaction is an important construct, as unsatisfying birth experiences are associated with the occurrence of postpartum depression and even posttraumatic stress disorder (…). Research has consistently identified control as one of these factors that greatly affects a woman’s assessment of the quality of her birth Issues of control during pregnancy and childbirth manifest themselves in three ways. These include prenatal control of fetal health during the pregnancy, expectations of control for labour and birth, and actual control experienced during childbirth (…)”. |
| **(57)** | “Birth satisfaction represents a woman’s subjective and uniquely personal evaluation of her birth experience. This complex, multifaceted construct includes elements of perceived quality of care, coping efficacy and reflections of the birth experience as a whole and in context. Birth satisfaction is thus a retrospective reconstruction related directly to the salient events surrounding the experience of birth (…). The woman’s individual evaluation of her own birth experience is important, as this may be a potent indicator of perinatal mental health outcome; for example, birth trauma, which would be anticipated to be experienced as a negative event, may be associated with the experience and manifestation of postpartum post-traumatic stress disorder (…)”. |
| **(58)** | “(…) Parents’ satisfaction with given care has been described as an indicator of care quality,14 and may be used to improve healthcare. Dissatisfaction with given care has been related to professionals lacking skills, giving inadequate information, and professionals restricted in number. To increase satisfaction with given care, the care should be individualized. The most important determinants for patient satisfaction, in general, have been related to respect for patient preferences, and giving emotional and physical support. (…)”. |
| **(59)** | “Birth satisfaction: "tells how a woman feels about her birth experience, which requires the midwife to take into consideration her personal wants and needs within confines of safety and cost (…). Markers of ‘birth satisfaction’ include, for example (…): considering person-centred preparation for childbirth, providing respect and support throughout the birth process, maintaining open and honest communication, affording a comfortable environment in which the woman is less likely to lose control, offering acceptable methods of pain relief, minimising obstetric injury, and helping the woman to give birth in her desired position (…). Levels of ‘birth satisfaction’ can affect the mental health of both mother and infant, with a negative experience having the potential to reduce mother–infant attachment, reduce willingness to breast-feed, instigate sexual dysfunction, instigate infant neglect/abuse, result in postnatal depression (PND), post-traumatic stress disorder (PTSD) and request for future elective cesarean section (CS), and lead to requests for sterilisation and/or abortion (…)”. |
| **(60)** | “Studies show that women accessing modern institutional health care still face many challenges including disrespectful, abusive, and inhumane ways of treatment, especially during labor and delivery processes. Such treatment violates the right of women to respectful care, and can also threaten their rights to life, health, bodily integrity, and freedom from dis- crimination (…). Evidence has shown that dissatisfied mothers, especially in the developing world like Ethiopia, tend to prefer utilizing traditional means of health care, using modern health care services as a last resort (…)”. |

**Table S5: Obstetric violence**

| **Key concept derived from conceptual ideas:** OV addresses facets of dehumanized care and any action or omission by both health personnel and the health care system that physically or psychologically damaged or denigrated a woman. OV includes medical negligence, improper medication, pathologizing of/inconsideration for natural processes of childbirth, postpartum and female reproductive processes, and forced sterilization. OV links to the concepts of structural and gender violence. Structural violence includes the lack of access to health care services and any kind of health discrimination due to a woman’s education, poverty, ethnicity, or other social vulnerabilities. | |
| --- | --- |
| **Study** | **Conceptual idea (individual studies in OV cluster)** |
| **(61)** | „The term obstetric violence is used to describe the various forms of violence that occur in the care of pregnancy, childbirth, postpartum and abortion. It is understood by the appropriation of the body and the reproductive processes of women by health professionals who express themselves through dehumanizing relations, abuse of medicalization and pathologization of natural processes resulting in loss of autonomy and ability to freely decide on their body and sexuality and negatively impacting the quality of life of women (…)” |
| **(62)** | „The definition of obstetric violence is, “the acts of dehumanizing treatment, abuse of procedures, and loss of autonomy that affect the quality of life of women.” It is important to realize that obstetric violence is considered another form of gender violence against women (…)”. |
| **(63)** | “Obstetric violence as a specific type of gender violence affecting women, rather than as a problem of poor- quality health care service or mistreatment and abuse in health care services that might affect any patient (…) This perspective, which is adopted in our study, allows mistreatment and abuse to be studied in the broader context of the various types of violence suffered by women. (…) this phenomenon was legally defined in Venezuela as: the appropriation of a woman’s body and reproductive processes by personnel, expressed as dehumanizing treatment, an abuse of medication, and the pathologization of natural processes, bringing about a loss of autonomy and the capacity to freely decide about their bodies and sexuality, negatively impacting the quality of life of women (…) “. |
| **(64)** | “Obstetric violence (OV) is a specific type of violation of women’s rights in medical practice during health care related to the childbirth processes. Laboring mothers may be subjected to different forms of OV during facility child birth.. Such ill-treatments and abuses create a psychological distance between the women and care providers and then drive women away from formal health care systems in fear of being subjected to such violence and sometimes are a more prominent hindrance than geographical or financial barriers to maternal health service utilization”. |
| **(65)** | “(…) Among the problems related to the health of pregnant women, concerns have been raised more recently regarding certain practices adopted in medical assistance, referred to by specialists as ‘institutional violence in childbirth’ or ‘obstetric violence’ (…) institutional violence is defined as the failure to act or any type of omission in health care services. This ranges from the broad level of lack of access to these services to their bad quality. (…) Some epidemiological studies have associated the occurrence of psychiatric disorders in the puerperal period, among them postpartum depression, with elements related to obstetrical care (…) such as feeling of abandonment during delivery, inadequate pain management, frustration for having delivered via cesarean section when natural childbirth was possible, and the pregnant woman’s perception of the team who provided the care”. |
| **(66)** | „Obstetric violence, a specific type of violation of women's rights, includes the right to equality, freedom, information, integrity, health, and reproductive autonomy (…). In Ecuador, the latest definition of obstetric violence has been extended to include the concept of ‘gynecological-obstetric violence’. It includes: abuse; imposing cultural practices and non- consented scientific procedures; violation of professional secrecy; improper medicalization; inconsideration for natural processes of pregnancy, childbirth, and postpartum; forced sterilization; loss of autonomy and women's incapacity to freely decide over their body and their sexuality; all of which can have a negative impact on women's quality of life, especially in regards to their sexual and reproductive health”. |
| **(67)** | “The law defines obstetric violence as ‘any action or omission of action by health personnel that damages, injures, denigrates or causes the death of a woman during pregnancy, birth and the puerperal period’ (…). More specifically, the law penalises medical negligence, which is expressed as ‘(1) dehumanised care; (2) abuse of medication and pathologisation of natural processes; (3) use of a caesarean section even when the conditions for a natural birth exist; (4) use of contraceptive methods or sterilisation without voluntary consent, and (5) interference in the early attachment between the newborn and his or her mother without medical justification, denying the mother the possibility of carrying and nursing the newborn immediately after birth (…)”. |

**Table S6: Person-centered care (PCC)**

| **Key concept derived from conceptual ideas:** Person-centered care is respectful of and responsive to individual patient preferences and needs, ensuring that the patients’ values guide all clinical decisions. Elements of PCC are 1. treating the patient with respect, 2. providing care in a non-threatening manner, 3. working in collaboration as equal partners, and 4. giving priority to the patient’s preferences over that of the healthcare provider. | |
| --- | --- |
| **Study** | **Conceptual idea (individual studies in PCC cluster)** |
| **(68)** | “Person-centered maternity care (PCMC) refers to “maternity care that is respectful of and responsive to individual women and their families’ preferences, needs, and values” (…). The WHO recommendations highlight respectful maternity care, effective communication, and companionship during labor and childbirth as key dimensions of PCMC that should be provided to every women throughout labor and birth (…). These recommendations are based on a human rights-based approach, as well as on evidence of the potential impacts of these interventions to reducing maternal morbidity and mortality (…).” |
| **(69)** | “Person-centered maternity care is “respectful of and responsive to individual women and their families’ preferences, needs, and values”—in accordance with the Institute of Medicine's definition of person-centered care, (…). The concepts of respectful maternity care (RMC) are incorporated in PCMC as part of the broader interest in person-centered care, (…) and capture the experience dimensions in the WHO vision for quality of maternal and newborn health (…).” |
| **(70)** | “PCC, defined as ‘care that is respectful of and responsive to individual patient preferences, needs, and values and ensuring that patient values guide all clinical decisions’ (…)Aspects of patient-centered care such as patient-provider communication and patient involvement in decision making are associated with higher levels of patient satisfaction, more trust in the provider, and better treatment adherence (…); in some studies patient-centered care is also associated with better health outcomes (…)”. |
| **(71)** | „ (…) patient-centered care has been held up as the ideal model of patient-provider interaction in all types of health- care; the implementation of patient-centered care is now recognized as an integral component of care quality (…). In an approach consistent with patient-centered care, clinicians respect and take into account individual patients’ preferences and values, and involve patients in decision-making (…). Along with this shift toward patient-centered care, patients are increasingly viewed as consumers (...). In this model, healthcare providers are charged with providing adequate information to patients to enable them to make decisions that best fit their preferences, while patients are charged with active involvement in making decisions about their treatment and following through to implement treatment plans (…)” |
| **(72)** | “From the evidence, care provided at birth centres can be called women-centred care (WCC).The four elements of WCC were respect, safety, holism, and partnership and its goal is the general well-being of women, potentially leading to the woman’s empowerment (…). (…) basic attitudes to be important in providing WCC: (1) treating women with respect, (2) providing care in a non-threatening manner, (3) working in collaboration as equal partners, and (4) giving priority to the woman’s preferences over that of the health-care provider (…)”. |

1. Azhar Z, Oyebode O, Masud H. Disrespect and abuse during childbirth in district Gujrat, Pakistan: A quest for respectful maternity care. *PloS one* (2018) 13(7):e0200318. Epub 2018/07/12. doi: 10.1371/journal.pone.0200318. PubMed PMID: 29995939; PubMed Central PMCID: PMCPMC6040717.

2. Bekele W, Bayou NB, Garedew MG. Magnitude of disrespectful and abusive care among women during facility-based childbirth in Shambu town, Horro Guduru Wollega zone, Ethiopia. *Midwifery* (2020) 83:102629. Epub 2020/01/26. doi: 10.1016/j.midw.2020.102629. PubMed PMID: 31981935.

3. Bhattacharya S, Sundari Ravindran TK. Silent voices: institutional disrespect and abuse during delivery among women of Varanasi district, northern India. *BMC pregnancy and childbirth* (2018) 18(1):338. Epub 2018/08/22. doi: 10.1186/s12884-018-1970-3. PubMed PMID: 30126357; PubMed Central PMCID: PMCPMC6102865.

4. Gebremichael MW, Worku A, Medhanyie AA, Berhane Y. Mothers' experience of disrespect and abuse during maternity care in northern Ethiopia. *Global health action* (2018) 11(sup3):1465215. Epub 2018/06/05. doi: 10.1080/16549716.2018.1465215. PubMed PMID: 29860934; PubMed Central PMCID: PMCPMC5990935.

5. Kruk ME, Kujawski S, Mbaruku G, Ramsey K, Moyo W, Freedman LP. Disrespectful and abusive treatment during facility delivery in Tanzania: a facility and community survey. *Health policy and planning* (2018) 33(1):e26-e33. Epub 2018/01/06. doi: 10.1093/heapol/czu079. PubMed PMID: 29304252.

6. Kujawski S, Mbaruku G, Freedman LP, Ramsey K, Moyo W, Kruk ME. Association Between Disrespect and Abuse During Childbirth and Women's Confidence in Health Facilities in Tanzania. *Matern Child Health J* (2015) 19(10):2243-50. Epub 2015/05/21. doi: 10.1007/s10995-015-1743-9. PubMed PMID: 25990843.

7. Sando D, Ratcliffe H, McDonald K, Spiegelman D, Lyatuu G, Mwanyika-Sando M, et al. The prevalence of disrespect and abuse during facility-based childbirth in urban Tanzania. *BMC pregnancy and childbirth* (2016) 16:236. Epub 2016/08/21. doi: 10.1186/s12884-016-1019-4. PubMed PMID: 27543002; PubMed Central PMCID: PMCPMC4992239.

8. Wassihun B, Deribe L, Worede N, Gultie T. Prevalence of disrespect and abuse of women during child birth and associated factors in Bahir Dar town, Ethiopia. *Epidemiology and health* (2018) 40:e2018029. Epub 2018/07/31. doi: 10.4178/epih.e2018029. PubMed PMID: 30056644; PubMed Central PMCID: PMCPMC6178351.

9. Banks KP, Karim AM, Ratcliffe HL, Betemariam W, Langer A. Jeopardizing quality at the frontline of healthcare: prevalence and risk factors for disrespect and abuse during facility-based childbirth in Ethiopia. *Health policy and planning* (2018) 33(3):317-27. Epub 2018/01/09. doi: 10.1093/heapol/czx180. PubMed PMID: 29309598; PubMed Central PMCID: PMCPMC5886294.

10. Montesinos-Segura R, Urrunaga-Pastor D, Mendoza-Chuctaya G, Taype-Rondan A, Helguero-Santin LM, Martinez-Ninanqui FW, et al. Disrespect and abuse during childbirth in fourteen hospitals in nine cities of Peru. *International journal of gynaecology and obstetrics: the official organ of the International Federation of Gynaecology and Obstetrics* (2018) 140(2):184-90. Epub 2017/10/19. doi: 10.1002/ijgo.12353. PubMed PMID: 29044510.

11. Asefa A, Bekele D. Status of respectful and non-abusive care during facility-based childbirth in a hospital and health centers in Addis Ababa, Ethiopia. *Reproductive health* (2015) 12:33. Epub 2015/04/19. doi: 10.1186/s12978-015-0024-9. PubMed PMID: 25890317; PubMed Central PMCID: PMCPMC4403719.

12. Galle A, Manaharlal H, Cumbane E, Picardo J, Griffin S, Osman N, et al. Disrespect and abuse during facility-based childbirth in southern Mozambique: a cross-sectional study. *BMC pregnancy and childbirth* (2019) 19(1):369. Epub 2019/10/24. doi: 10.1186/s12884-019-2532-z. PubMed PMID: 31640603; PubMed Central PMCID: PMCPMC6805678.

13. Ukke GG, Gurara MK, Boynito WG. Disrespect and abuse of women during childbirth in public health facilities in Arba Minch town, south Ethiopia - a cross-sectional study. *PloS one* (2019) 14(4):e0205545. Epub 2019/04/30. doi: 10.1371/journal.pone.0205545. PubMed PMID: 31034534; PubMed Central PMCID: PMCPMC6488058.

14. Sethi R, Gupta S, Oseni L, Mtimuni A, Rashidi T, Kachale F. The prevalence of disrespect and abuse during facility-based maternity care in Malawi: evidence from direct observations of labor and delivery. *Reproductive health* (2017) 14(1):111. Epub 2017/09/08. doi: 10.1186/s12978-017-0370-x. PubMed PMID: 28877701; PubMed Central PMCID: PMCPMC5588731.

15. Morton CH, Henley MM, Seacrist M, Roth LM. Bearing witness: United States and Canadian maternity support workers' observations of disrespectful care in childbirth. *Birth* (2018) 45(3):263-74. Epub 2018/07/31. doi: 10.1111/birt.12373. PubMed PMID: 30058157.

16. Diamond-Smith N, Treleaven E, Murthy N, Sudhinaraset M. Women's empowerment and experiences of mistreatment during childbirth in facilities in Lucknow, India: results from a cross-sectional study. *BMC pregnancy and childbirth* (2017) 17(Suppl 2):335. Epub 2017/11/17. doi: 10.1186/s12884-017-1501-7. PubMed PMID: 29143668; PubMed Central PMCID: PMCPMC5688442.

17. Hameed W, Avan BI. Women's experiences of mistreatment during childbirth: A comparative view of home- and facility-based births in Pakistan. *PloS one* (2018) 13(3):e0194601. Epub 2018/03/17. doi: 10.1371/journal.pone.0194601. PubMed PMID: 29547632; PubMed Central PMCID: PMCPMC5856402.

18. Diamond-Smith N, Sudhinaraset M, Melo J, Murthy N. The relationship between women's experiences of mistreatment at facilities during childbirth, types of support received and person providing the support in Lucknow, India. *Midwifery* (2016) 40:114-23. Epub 2016/07/19. doi: 10.1016/j.midw.2016.06.014. PubMed PMID: 27428107.

19. Vedam S, Stoll K, Taiwo TK, Rubashkin N, Cheyney M, Strauss N, et al. The Giving Voice to Mothers study: inequity and mistreatment during pregnancy and childbirth in the United States. *Reproductive health* (2019) 16(1):77. Epub 2019/06/12. doi: 10.1186/s12978-019-0729-2. PubMed PMID: 31182118; PubMed Central PMCID: PMCPMC6558766.

20. Bohren MA, Mehrtash H, Fawole B, Maung TM, Balde MD, Maya E, et al. How women are treated during facility-based childbirth in four countries: a cross-sectional study with labour observations and community-based surveys. *Lancet* (2019) 394(10210):1750-63. doi: 10.1016/S0140-6736(19)31992-0. PubMed PMID: WOS:000496920300030.

21. Sheferaw ED, Kim YM, van den Akker T, Stekelenburg J. Mistreatment of women in public health facilities of Ethiopia. *Reproductive health* (2019) 16(1):130. Epub 2019/08/29. doi: 10.1186/s12978-019-0781-y. PubMed PMID: 31455400; PubMed Central PMCID: PMCPMC6712647.

22. Dey A, Shakya HB, Chandurkar D, Kumar S, Das AK, Anthony J, et al. Discordance in self-report and observation data on mistreatment of women by providers during childbirth in Uttar Pradesh, India. *Reproductive health* (2017) 14(1):149. Epub 2017/11/17. doi: 10.1186/s12978-017-0409-z. PubMed PMID: 29141640; PubMed Central PMCID: PMCPMC5688759.

23. Siraj A, Teka W, Hebo H. Prevalence of disrespect and abuse during facility based child birth and associated factors, Jimma University Medical Center, Southwest Ethiopia. *BMC pregnancy and childbirth* (2019) 19(1):185. Epub 2019/05/28. doi: 10.1186/s12884-019-2332-5. PubMed PMID: 31132988; PubMed Central PMCID: PMCPMC6537397.

24. Okafor, II, Ugwu EO, Obi SN. Disrespect and abuse during facility-based childbirth in a low-income country. *International journal of gynaecology and obstetrics: the official organ of the International Federation of Gynaecology and Obstetrics* (2015) 128(2):110-3. Epub 2014/12/06. doi: 10.1016/j.ijgo.2014.08.015. PubMed PMID: 25476154.

25. Silveira MF, Mesenburg MA, Bertoldi AD, De Mola CL, Bassani DG, Domingues MR, et al. The association between disrespect and abuse of women during childbirth and postpartum depression: Findings from the 2015 Pelotas birth cohort study. *J Affect Disord* (2019) 256:441-7. Epub 2019/06/30. doi: 10.1016/j.jad.2019.06.016. PubMed PMID: 31252237; PubMed Central PMCID: PMCPMC6880287.

26. Tekle Bobo F, Kebebe Kasaye H, Etana B, Woldie M, Feyissa TR. Disrespect and abuse during childbirth in Western Ethiopia: Should women continue to tolerate? *PloS one* (2019) 14(6):e0217126. Epub 2019/06/08. doi: 10.1371/journal.pone.0217126. PubMed PMID: 31173588; PubMed Central PMCID: PMCPMC6555589.

27. Abuya T, Sripad P, Ritter J, Ndwiga C, Warren CE. Measuring mistreatment of women throughout the birthing process: implications for quality of care assessments. *Reprod Health Matters* (2018) 26(53):48-61. Epub 2018/09/14. doi: 10.1080/09688080.2018.1502018. PubMed PMID: 30212308.

28. Bakker R, Sheferaw ED, Stekelenburg J, Yigzaw T, de Kroon MLA. Development and use of a scale to assess gender differences in appraisal of mistreatment during childbirth among Ethiopian midwifery students. *PloS one* (2020) 15(1):e0227958. Epub 2020/01/17. doi: 10.1371/journal.pone.0227958. PubMed PMID: 31945110; PubMed Central PMCID: PMCPMC6964878.

29. Baranowska B, Doroszewska A, Kubicka-Kraszynska U, Pietrusiewicz J, Adamska-Sala I, Kajdy A, et al. Is there respectful maternity care in Poland? Women's views about care during labor and birth. *BMC pregnancy and childbirth* (2019) 19(1):520. Epub 2019/12/25. doi: 10.1186/s12884-019-2675-y. PubMed PMID: 31870323; PubMed Central PMCID: PMCPMC6929297.

30. Asefa A, Bekele D, Morgan A, Kermode M. Service providers' experiences of disrespectful and abusive behavior towards women during facility based childbirth in Addis Ababa, Ethiopia. *Reproductive health* (2018) 15(1):4. Epub 2018/01/07. doi: 10.1186/s12978-017-0449-4. PubMed PMID: 29304814; PubMed Central PMCID: PMCPMC5756390.

31. Rosen HE, Lynam PF, Carr C, Reis V, Ricca J, Bazant ES, et al. Direct observation of respectful maternity care in five countries: a cross-sectional study of health facilities in East and Southern Africa. *BMC pregnancy and childbirth* (2015) 15(1):306. Epub 2015/11/26. doi: 10.1186/s12884-015-0728-4. PubMed PMID: 26596353; PubMed Central PMCID: PMCPMC4657214.

32. Wassihun B, Zeleke S. Compassionate and respectful maternity care during facility based child birth and women's intent to use maternity service in Bahir Dar, Ethiopia. *BMC pregnancy and childbirth* (2018) 18(1):294. Epub 2018/07/11. doi: 10.1186/s12884-018-1909-8. PubMed PMID: 29986659; PubMed Central PMCID: PMCPMC6038196.

33. Dynes MM, Twentyman E, Kelly L, Maro G, Msuya AA, Dominico S, et al. Patient and provider determinants for receipt of three dimensions of respectful maternity care in Kigoma Region, Tanzania-April-July, 2016. *Reproductive health* (2018) 15(1):41. Epub 2018/03/07. doi: 10.1186/s12978-018-0486-7. PubMed PMID: 29506559; PubMed Central PMCID: PMCPMC5838967.

34. Bante A, Teji K, Seyoum B, Mersha A. Respectful maternity care and associated factors among women who delivered at Harar hospitals, eastern Ethiopia: a cross-sectional study. *BMC pregnancy and childbirth* (2020) 20(1):86. Epub 2020/02/12. doi: 10.1186/s12884-020-2757-x. PubMed PMID: 32041564; PubMed Central PMCID: PMCPMC7011506.

35. Begley C, Sedlicka N, Daly D. Respectful and disrespectful care in the Czech Republic: an online survey. *Reproductive health* (2018) 15(1):198. Epub 2018/12/06. doi: 10.1186/s12978-018-0648-7. PubMed PMID: 30514394; PubMed Central PMCID: PMCPMC6280471.

36. Sheferaw ED, Bazant E, Gibson H, Fenta HB, Ayalew F, Belay TB, et al. Respectful maternity care in Ethiopian public health facilities. *Reproductive health* (2017) 14(1):60. Epub 2017/05/18. doi: 10.1186/s12978-017-0323-4. PubMed PMID: 28511685; PubMed Central PMCID: PMCPMC5434569.

37. Taavoni S, Goldani Z, Rostami Gooran N, Haghani H. Development and Assessment of Respectful Maternity Care Questionnaire in Iran. *Int J Community Based Nurs Midwifery* (2018) 6(4):334-49. Epub 2018/11/23. PubMed PMID: 30465006; PubMed Central PMCID: PMCPMC6226608.

38. Mukamurigo JU, Berg M, Ntaganira J, Nyirazinyoye L, Dencker A. Associations between perceptions of care and women's childbirth experience: a population-based cross-sectional study in Rwanda. *BMC pregnancy and childbirth* (2017) 17(1):181. Epub 2017/06/11. doi: 10.1186/s12884-017-1363-z. PubMed PMID: 28599645; PubMed Central PMCID: PMCPMC5466750.

39. Okumus F. Birth experiences of primiparous Turkish women: public and private hospitals. *Journal of Asian Midwives* (2017) 4(1):35-46. PubMed PMID: 124425275. Language: English. Entry Date: 20180117. Revision Date: 20190304. Publication Type: Article.

40. Alzyoud F, Khoshnood K, Alnatour A, Oweis A. Exposure to verbal abuse and neglect during childbirth among Jordanian women. *Midwifery* (2018) 58:71-6. Epub 2018/01/08. doi: 10.1016/j.midw.2017.12.008. PubMed PMID: 29306737.

41. Thies-Lagergren L, Johansson M. Intrapartum midwifery care impact Swedish couple's birth experiences - A cross-sectional study. *Women Birth* (2019) 32(3):213-20. Epub 2018/09/16. doi: 10.1016/j.wombi.2018.08.163. PubMed PMID: 30217554.

42. Overgaard C, Fenger-Gron M, Sandall J. The impact of birthplace on women's birth experiences and perceptions of care. *Soc Sci Med* (2012) 74(7):973-81. Epub 2012/02/14. doi: 10.1016/j.socscimed.2011.12.023. PubMed PMID: 22326105.

43. Redshaw M, Martin CR, Savage-McGlynn E, Harrison S. Women's experiences of maternity care in England: preliminary development of a standard measure. *BMC pregnancy and childbirth* (2019) 19(1):167. Epub 2019/05/16. doi: 10.1186/s12884-019-2284-9. PubMed PMID: 31088487; PubMed Central PMCID: PMCPMC6518811.

44. Sjetne IS, Iversen HH, Kjollesdal JG. A questionnaire to measure women's experiences with pregnancy, birth and postnatal care: instrument development and assessment following a national survey in Norway. *BMC pregnancy and childbirth* (2015) 15:182. Epub 2015/08/22. doi: 10.1186/s12884-015-0611-3. PubMed PMID: 26294064; PubMed Central PMCID: PMCPMC4546178.

45. Monazea EM, Al-Attar GS. Quality of delivery care in Assiut University Hospital, Egypt: mothers' satisfaction. *The Journal of the Egyptian Public Health Association* (2015) 90(2):64-71. Epub 2015/07/15. doi: 10.1097/01.EPX.0000466380.29269.4b. PubMed PMID: 26154833.

46. Kabakian-Khasholian T, Bashour H, El-Nemer A, Kharouf M, Sheikha S, El Lakany N, et al. Women's satisfaction and perception of control in childbirth in three Arab countries. *Reprod Health Matters* (2017) 25(sup1):16-26. Epub 2017/11/10. doi: 10.1080/09688080.2017.1381533. PubMed PMID: 29120285.

47. Jha P, Larsson M, Christensson K, Skoog Svanberg A. Satisfaction with childbirth services provided in public health facilities: results from a cross- sectional survey among postnatal women in Chhattisgarh, India. *Global health action* (2017) 10(1):1386932. Epub 2017/11/01. doi: 10.1080/16549716.2017.1386932. PubMed PMID: 29087240; PubMed Central PMCID: PMCPMC5678347.

48. Gungor I, Beji NK. Development and psychometric testing of the scales for measuring maternal satisfaction in normal and caesarean birth. *Midwifery* (2012) 28(3):348-57. Epub 2011/05/07. doi: 10.1016/j.midw.2011.03.009. PubMed PMID: 21546142.

49. Hollins Martin CJ, Martin CR. Development and psychometric properties of the Birth Satisfaction Scale-Revised (BSS-R). *Midwifery* (2014) 30(6):610-9. Epub 2013/11/21. doi: 10.1016/j.midw.2013.10.006. PubMed PMID: 24252712.

50. Gitobu CM, Gichangi PB, Mwanda WO. Satisfaction with Delivery Services Offered under the Free Maternal Healthcare Policy in Kenyan Public Health Facilities. *J Environ Public Health* (2018) 2018:4902864. Epub 2018/06/29. doi: 10.1155/2018/4902864. PubMed PMID: 29951103; PubMed Central PMCID: PMCPMC5987322.

51. Caballero P, Delgado-Garcia BE, Orts-Cortes I, Moncho J, Pereyra-Zamora P, Nolasco A. Validation of the Spanish version of Mackey childbirth satisfaction rating scale. *BMC pregnancy and childbirth* (2016) 16:78. Epub 2016/04/17. doi: 10.1186/s12884-016-0862-7. PubMed PMID: 27084092; PubMed Central PMCID: PMCPMC4833934.

52. Bitew K, Ayichiluhm M, Yimam K. Maternal Satisfaction on Delivery Service and Its Associated Factors among Mothers Who Gave Birth in Public Health Facilities of Debre Markos Town, Northwest Ethiopia. *Biomed Res Int* (2015) 2015:460767. Epub 2015/09/09. doi: 10.1155/2015/460767. PubMed PMID: 26347882; PubMed Central PMCID: PMCPMC4546969.

53. Haines HM, Hildingsson I, Pallant JF, Rubertsson C. The role of women's attitudinal profiles in satisfaction with the quality of their antenatal and intrapartum care. *J Obstet Gynecol Neonatal Nurs* (2013) 42(4):428-41. Epub 2013/06/19. doi: 10.1111/1552-6909.12221. PubMed PMID: 23773005.

54. Mehata S, Paudel YR, Dariang M, Aryal KK, Paudel S, Mehta R, et al. Factors determining satisfaction among facility-based maternity clients in Nepal. *BMC pregnancy and childbirth* (2017) 17(1):319. Epub 2017/09/28. doi: 10.1186/s12884-017-1532-0. PubMed PMID: 28946851; PubMed Central PMCID: PMCPMC5613378.

55. Conesa Ferrer MB, Canteras Jordana M, Ballesteros Meseguer C, Carrillo Garcia C, Martinez Roche ME. Comparative study analysing women's childbirth satisfaction and obstetric outcomes across two different models of maternity care. *BMJ open* (2016) 6(8):e011362. Epub 2016/08/28. doi: 10.1136/bmjopen-2016-011362. PubMed PMID: 27566632; PubMed Central PMCID: PMCPMC5013466.

56. Fair CD, Morrison TE. The relationship between prenatal control, expectations, experienced control, and birth satisfaction among primiparous women. *Midwifery* (2012) 28(1):39-44. Epub 2011/04/05. doi: 10.1016/j.midw.2010.10.013. PubMed PMID: 21458895.

57. Vardavaki Z, Martin CJH, Martin CR. Construct and content validity of the Greek version of the Birth Satisfaction Scale (G-BSS). *Journal of Reproductive and Infant Psychology* (2015) 33(5):488-503. doi: 10.1080/02646838.2015.1035235. PubMed PMID: WOS:000361965500005.

58. Johansson M, Hildingsson I. Intrapartum care could be improved according to Swedish fathers: mode of birth matters for satisfaction. *Women Birth* (2013) 26(3):195-201. Epub 2013/05/15. doi: 10.1016/j.wombi.2013.04.001. PubMed PMID: 23664433.

59. Goncu Serhatlioglu S, Karahan N, Hollins Martin CJ, Martin CR. Construct and content validity of the Turkish Birth Satisfaction Scale - Revised (T-BSS-R). *J Reprod Infant Psychol* (2018) 36(3):235-45. Epub 2018/03/20. doi: 10.1080/02646838.2018.1443322. PubMed PMID: 29553295.

60. Gashaye KT, Tsegaye AT, Shiferaw G, Worku AG, Abebe SM. Client satisfaction with existing labor and delivery care and associated factors among mothers who gave birth in university of Gondar teaching hospital; Northwest Ethiopia: Institution based cross-sectional study. *PloS one* (2019) 14(2):e0210693. Epub 2019/02/07. doi: 10.1371/journal.pone.0210693. PubMed PMID: 30726297; PubMed Central PMCID: PMCPMC6364872.

61. Da Silva MC, Feijó BDM, Pereira FANS, Guerra FJF, Santos ISd, Rodrigues GDO, et al. Parto e nascimento na região rural: a violência obstétrica. *Revista de Enfermagem UFPE on line* (2018) 12(9):2407-17. doi: 10.5205/1981-8963-v12i9a234440p2407-2417-2018. PubMed PMID: 131728246. Language: English. Entry Date: 20180919. Revision Date: 20181010. Publication Type: Article. Journal Subset: Mexico & Central/South America.

62. Brandao T, Canadas S, Galvis A, de Los Rios MM, Meijer M, Falcon K. Childbirth experiences related to obstetric violence in public health units in Quito, Ecuador. *International journal of gynaecology and obstetrics: the official organ of the International Federation of Gynaecology and Obstetrics* (2018) 143(1):84-8. Epub 2018/07/20. doi: 10.1002/ijgo.12625. PubMed PMID: 30025157.

63. Castro R, Frias SM. Obstetric Violence in Mexico: Results From a 2016 National Household Survey. *Violence Against Women* (2020) 26(6-7):555-72. Epub 2019/04/09. doi: 10.1177/1077801219836732. PubMed PMID: 30957706.

64. Mihret MS. Obstetric violence and its associated factors among postnatal women in a Specialized Comprehensive Hospital, Amhara Region, Northwest Ethiopia. *BMC Res Notes* (2019) 12(1):600. Epub 2019/09/20. doi: 10.1186/s13104-019-4614-4. PubMed PMID: 31533858; PubMed Central PMCID: PMCPMC6751597.

65. Souza KJ, Rattner D, Gubert MB. Institutional violence and quality of service in obstetrics are associated with postpartum depression. *Revista de saude publica* (2017) 51:69. Epub 2017/07/27. doi: 10.1590/S1518-8787.2017051006549. PubMed PMID: 28746574; PubMed Central PMCID: PMCPMC5510781.

66. Meijer M, Brandao T, Canadas S, Falcon K. Components of obstetric violence in health facilities in Quito, Ecuador: A descriptive study on information, accompaniment, and position during childbirth. *International journal of gynaecology and obstetrics: the official organ of the International Federation of Gynaecology and Obstetrics* (2020) 148(3):355-60. Epub 2019/12/01. doi: 10.1002/ijgo.13075. PubMed PMID: 31785159.

67. Montoya A, Fritz J, Labora A, Rodriguez M, Walker D, Trevino-Siller S, et al. Respectful and evidence-based birth care in Mexico (or lack thereof): An observational study. *Women Birth* (2020) 33(6):574-82. Epub 2020/03/01. doi: 10.1016/j.wombi.2020.02.011. PubMed PMID: 32111555.

68. Afulani PA, Diamond-Smith N, Phillips B, Singhal S, Sudhinaraset M. Validation of the person-centered maternity care scale in India. *Reproductive health* (2018) 15(1):147. Epub 2018/08/31. doi: 10.1186/s12978-018-0591-7. PubMed PMID: 30157877; PubMed Central PMCID: PMCPMC6114501.

69. Afulani PA, Feeser K, Sudhinaraset M, Aborigo R, Montagu D, Chakraborty N. Toward the development of a short multi-country person-centered maternity care scale. *International journal of gynaecology and obstetrics: the official organ of the International Federation of Gynaecology and Obstetrics* (2019) 146(1):80-7. Epub 2019/04/21. doi: 10.1002/ijgo.12827. PubMed PMID: 31004349.

70. Attanasio L, Kozhimannil KB. Patient-reported Communication Quality and Perceived Discrimination in Maternity Care. *Med Care* (2015) 53(10):863-71. Epub 2015/09/05. doi: 10.1097/MLR.0000000000000411. PubMed PMID: 26340663; PubMed Central PMCID: PMCPMC4570858.

71. Attanasio L, Hardeman R. Declined care and discrimination during the childbirth hospitalization. *Soc Sci Med* (2019) 232:270-7. Epub 2019/05/22. doi: 10.1016/j.socscimed.2019.05.008. PubMed PMID: 31112918.

72. Iida M, Horiuchi S, Porter SE. The relationship between women-centred care and women's birth experiences: a comparison between birth centres, clinics, and hospitals in Japan. *Midwifery* (2012) 28(4):398-405. Epub 2011/08/13. doi: 10.1016/j.midw.2011.07.002. PubMed PMID: 21835515.
