# Supplementary material for: A critical review: developing a birth integrity framework for epidemiological studies through meta-ethnography
Source: BMC Womens Health. 2023 Oct 10;23:530. doi: 10.1186/s12905-023-02670-z (PMC10565979; doi:10.1186/s12905-023-02670-z)
Supplement: Supplementary file 6 — Additional file 6. Measurement level: Process of identifying themes. [file 12905_2023_2670_MOESM6_ESM.docx]

**Additional file 6: Measurement level: Process of identifying themes**

**Process of identifying themes in items (example for RMC conceptual cluster)**

| **Study** | **Themes** | **Items (as stated in the original studies)** |
| --- | --- | --- |
| **(Bante et al., 2020)** | Empathy  Responsiveness  Physical abuse  Verbal abuse | The health worker showed his/her concern and empathy.  Some healthcare providers slapped me during delivery for different reasons.  The healthcare workers responded to my needs whether or not I asked.  Some health workers shouted at me because I haven’t done what I was told to do. |
| **(Rosen et al., 2015)** | Auditory and visual privacy | Delivery in rooms with auditory and visual privacy  Provider drapes client before delivery. |
| **(Dynes et al., 2018)** | Physical abuse | Did any of the health facility staff ever physically abuse you during your visit? By physical abuse, we mean, did they hit, slap, push, kick you, or use any other type of physical force against you (Absence of physical abuse)? |

| **Themes in study items (sorted by conceptual clusters)** | | | | | | | | | | | | | | | |
| --- | --- | --- | --- | --- | --- | --- | --- | --- | --- | --- | --- | --- | --- | --- | --- |
| **Themes** | **D&A, MISC** | **RMC** | **OV** | **PCC** | **CE** | **MS** | **Other** | **Themes** | **D&A, MISC** | **RMC** | **OV** | **PCC** | **CE** | **MS** | **Other** |
| Availability of health facility |  |  |  |  |  | x |  | Provision of written information |  |  |  |  |  |  | RESP |
| Availability of medical services in facility |  |  | x |  |  |  | RESP | Admission, service, and information management |  |  |  |  | x | x | RESP |
| Accessibility of health facility (from home) |  |  |  |  |  | x |  | Equipment to protect privacy | x | x | x |  |  |  | RESP |
| Conformity of childbirth expectations and experiences |  |  |  |  |  | x |  | Availability of pain relief medication and comfort measures | x |  |  |  |  | x |  |
| Staffing capacity | x |  |  | x | x | x |  | Medical assessment | x |  |  |  |  |  |  |
| Continuity and choice of the care provider |  |  |  |  |  | x | RESP | Comfortability and conditions of amenities |  |  |  |  |  | x | MW |
| Timely care | x | x |  | x |  | x | MW | Basic equipment | x |  |  | x |  | x |  |
| Detention in facility | x |  | x |  |  |  |  | Provision of translation | x |  |  |  |  |  |  |
| Information on individual proceeding and medical diagnosis | x | x |  |  | x | x | MES | General information on labor, birth, postpartum stage, and newborn care | x | x |  |  | x | x |  |
| Cleanliness and hygienic conditions of facility | x |  |  | x |  | x | RESP | Appropriate length of hospitalization |  |  |  |  | x |  |  |
| Hygienic practices | x |  |  |  |  |  |  | Information on choices | x |  |  |  |  |  | PVP |
| Provision of safe medical care |  |  |  |  | x | x | MES, PVP | Consistency of information |  |  |  |  |  | x |  |
| Adherence to medical guidelines and evidence-based care practices |  |  | x |  |  | x |  | Explanation of procedures or examinations before proceeding | x | x | x | x |  |  | MES |
| Competency of health provider |  |  |  |  | x | x |  | (Non-)Consented care | x |  | x | x |  | x | MES, IC, RESP |
| Encouragement to ask questions | x | x |  |  |  | x | SEC, RESP | Involvement and empowerment |  |  |  | x |  |  |  |
| (Non-) Discrimination based upon financial or insurance status | x | x |  | x |  |  | MES, MW, MOR | (Non-)Discrimination based upon individual’s attributes | x | x | x | x |  | x | MW, MOR |
| (Non-)Effective communication | x |  |  | x |  |  | PVP, RESP | (Non-) Discrimination based upon disagreement | x | x |  | x |  |  | MW, MOR |
| **Themes** | **D&A, MISC** | **RMC** | **OV** | **PCC** | **CE** | **MS** | **Other** | **Themes** | **D&A, MISC** | **RMC** | **OV** | **PCC** | **CE** | **MS** | **Other** |
| Engagement and Empathy |  | x |  | x |  |  |  | Internal control |  |  |  |  | x | x | SEC, RESP, SC |
| (Non-)Respectful communication | x |  | x | x | x | x | MW | Perceived societal discriminatory practice | x |  |  |  |  |  |  |
| Verbal abuse | x | x | x | x | x |  | MES | Bodily autonomy | x |  | x |  |  | x | IC |
| Emotional abuse | x |  |  | x | x |  |  | Parental rights | x |  | x |  |  | x |  |
| Presence and absence of health professionals | x | x | x |  | x | x |  | Provision/ denial of requested care or pain medication | x |  | x |  | x |  | RESP |
| Physical abuse (force) | x | x | x | x |  |  |  | Bribery | x |  |  |  |  |  | MES, RESP |
| Physical abuse (restraint) | x | x | x | x |  |  |  | (Non-)Respectful care | x | x | x | x | x | x | RESP |
| External control |  |  |  |  | x | x | SEC, RESP, SC | Attitudes towards childbirth |  |  |  |  |  | x |  |
| Sexual abuse | x |  |  |  |  |  | MES | Expectations of control |  |  |  |  |  | x |  |
| Visual privacy | x | x | x |  | x |  |  | Preferences and wishes | x |  | x |  |  |  |  |
| Cooperation between health professionals |  |  |  |  |  | x |  | Perception of violent birth experiences |  |  | x |  |  |  |  |
| Visual privacy | x | x | x |  | x |  |  | Coercion | x |  | x |  |  |  |  |
| Auditory privacy | x |  |  |  |  |  |  | Agentry |  |  |  | x |  | x |  |
| Stress, anxiety, fear |  |  |  |  | x | x | SEC, RESP, SC | Pain perception |  |  |  |  | x | x | SEC, RESP, SC |
| Satisfaction with self |  |  |  |  |  | x |  | Situational privacy | x |  |  |  | x | x | MES, RESP |
| (Non-)Confidential handling of sensitive data | x |  | x | x |  |  | MES, RESP | Engagement and Empathy |  | x |  | x | x |  |  |
| Bonding and attachment |  |  |  | x |  |  |  | Depression |  |  |  |  |  | x |  |
| Pain management | x |  |  | x |  | x | SEC | Security, safety, trust, feeling of being seen | x | x |  | x | x | x | SEC, RESP, SC |
| Posttraumatic stress |  |  |  |  |  |  | SEC | Attitudes towards gender norms |  |  |  | x |  |  |  |
