# Supplementary material for: A critical review: developing a birth integrity framework for epidemiological studies through meta-ethnography
Source: BMC Womens Health. 2023 Oct 10;23:530. doi: 10.1186/s12905-023-02670-z (PMC10565979; doi:10.1186/s12905-023-02670-z)
Supplement: Supplementary file 3 — Additional file 3. PRISMA 2020 flow diagram and adapted PRISMA for reporting systematic reviews reporting of qualitative and quantitative evidence. [file 12905_2023_2670_MOESM3_ESM.docx]

**Additional file 3: PRISMA 2020 flow diagram and adapted PRISMA for reporting systematic reviews reporting of qualitative and quantitative evidence**

**Identification of studies via databases and registers**

Records removed *before screening*:

Duplicate records removed (n = **473**)

Records identified from:

Pubmed, PsychInfo, Embase, CINAHL (n =**9626** (8689 + 937 in updated search))

**Identification**

Records screened

(n = 9153)

Records excluded**

(n = 9046)

**Screening**

Studies excluded:

On (medical) intervention (n = 7)

Out-of-hospital birth (n =1)

Language (n = 3)

Not on topic (n=6)

Study design (n=3)

Fulltexts not available (n=5)

Studies assessed for eligibility

(n = 107)

Studies included in review

(n = 82 )

**Included**

*Consider, if feasible to do so, reporting the number of records identified from each database or register searched (rather than the total number across all databases/registers).

**If automation tools were used, indicate how many records were excluded by a human and how many were excluded by automation tools.

*From:* Page MJ, McKenzie JE, Bossuyt PM, Boutron I, Hoffmann TC, Mulrow CD, et al. The PRISMA 2020 statement: an updated guideline for reporting systematic reviews. BMJ 2021;372:n71. doi: 10.1136/bmj.n71

For more information, visit: <http://www.prisma-statement.org/>

**Adapted PRISMA for reporting systematic reviews of qualitative and quantitative evidence**

| **Section/topic** | **Description** | |
| --- | --- | --- |
| **TITLE** | | |
| Title | Propose a short take-home title. The title should explicitly state that the review included different type of evidence. | See page 1. We identify the review as critical review and state that we included epidemiological studies. |
| **ABSTRACT** | | |
| Structured summary | Provide a structured summary including, as applicable: background; objectives; data sources; study eligibility criteria, participants, and interventions; study appraisal and synthesis methods; results; limitations; conclusions and implications of key findings; systematic review registration number. | See abstract (page 1) |
| **INTRODUCTION AND OBJECTIVES** | | |
| Rationale | Describe the rationale for the review (e.g., a health problem) in the context of what is already known (e.g., an existing literature review paper or a reference book chapter). | See page 2-3 |
| Objectives | Formulate questions and/or objectives (qualitative, quantitative or both) being addressed by your review. | See page 3 |
| **METHODS** | | |
| Protocol and registration | Indicate if a review protocol exists, if and where it can be accessed (e.g., Web address), and, if available, provide registration information including registration number. | No registered review protocol exists. |
| Justification | Justify the use of a review of qualitative and quantitative  evidence. | This review used a qualitative method to assess concepts and methods used in quantitative studies. |
| Eligibility criteria | Specify the inclusion and exclusion criteria and the rationale for supporting these criteria. | See page 3-4 and appendix data file A |
| Information sources | Describe all information sources (e.g., databases with dates of coverage, contact with study authors to identify additional studies) in the search and date last searched. | See page 3 and appendix data file A, were applicable. |
| Search | Present full electronic search strategy for at least one database (e.g., in an appendix), including any limits used, such that it could be repeated.  Describe the process for removing duplicates. | See appendix data file A |
| Study selection |  | See page 3 and 4 and appendix file B + file C (PRISMA Flowchart) |
| Data collection process | Describe the process for selecting studies (e.g., screening based on titles and abstracts, and eligibility based on full-text, number of reviewers, software used).  Describe the method of data extraction from included studies (e.g., number of reviewers involved, piloted forms, etc.).  List the data extracted. | See appendix file A |
| Appraisal | Describe the process for appraising included studies (e.g., tools used, number of reviewers involved), and specifically for assessing the methodological quality or risk of bias of included qualitative, quantitative, and mixed methods studies.  Specify how results of this appraisal are used in the synthesis. For example, for descriptive purpose (include all studies with description of their methodological quality or risk of bias) or for analytical purpose (contrast synthesis of ‘lower quality’ studies vs. ‘higher quality’ studies using sensitivity analysis). | Not applied as the critical review was not focused on outcomes. |
| Synthesis | Describe the synthesis design used. Describe and justify the synthesis method(s) used (e.g., quantitative content analysis, meta-analysis, thematic synthesis, etc.). | See page 4-5 and Table 1. |
| Additional analysis | Describe methods of additional analyses (e.g., sensitivity or subgroup analyses), if done. | Not applied. |
| **RESULTS** | | |
| Study selection | Give numbers of studies screened, assessed for eligibility, and included in the review, with reasons for exclusions at each stage.  Summarize in a flow diagram (see Appendices). | See page 7 andappendix file C (PRISMA Flowchart) |

| Study characteristics |  | | For each study, present characteristics for which data were extracted (e.g., tables of characteristics of included studies – see Appendices) and provide the citations. | | See appendix file D. |
| --- | --- | --- | --- | --- | --- |
|  |  | | Specify common information across all included studies. | |  |
|  |  | | Describe the studies including their heterogeneity (variability associated with differences between studies). | | Not applied due to study objective |
| Result of appraisal |  | | Present data on the methodological quality or risk of bias of included studies based on the appraisal done. | | Not applied due to study objective |
| Results of synthesis |  | | Present results of synthesis. | | See pages 7-22, Tables 2-5 and Figure 1 |
|  |  | | If qualitative synthesis: In the text, briefly summarize the main themes or categories and refer to the appendix. | |  |
|  |  | | Appendix (table, figure, or matrix): For each study, present the themes or categories identified | |  |
|  |  | |  | |  |
| Additional analysis |  | | Give results of additional analyses, if done (e.g., sensitivity or subgroup analyses). | | Not applied. |
| **DISCUSSION** | |  | | | |
| Summary of evidence |  | | Provide an overall summary of results (take-home messages) from the qualitative and/or quantitative synthesis. | | See page 26-30, |
|  |  | | State the main results for each main theme or category, and/or key process/outcome variable. | |  |
|  |  | | Consider their relevance and importance for knowledge users (e.g., health care providers, managers, and decision/policy makers). | |  |
|  |  | | Take into account the methodological quality across studies (when applicable). | |  |
|  |  | | Describe insight gained from the integration of qualitative and quantitative evidence. | |  |
| Contribution |  | | Describe the contribution of the review (compared to what is already known) with respect to Review methods, Scientific knowledge, Practice, program planning and evaluation, policy making, or anything else | | See page 27-30 |
| Limitations |  | | Specify any element that may affect the cumulative evidence. | | Not applicable |
|  |  | | Discuss limitations at the study and process/outcome levels (e.g., lack of rich data for qualitative synthesis, methodological quality/risk of bias, and their potential consequences on the results). | | Not applicable |
|  |  | | Discuss limitations at the review level (e.g., dependent reviewers, incomplete retrieval of relevant studies - selective publication of reports regarding studies with positive results), and limited reporting (selective reporting of information about included studies)), and their potential consequences on the results. | | See pages 31-21 |
| Conclusions |  | | Provide a general interpretation of the results in the context of other evidence, including implications for knowledge users (e.g., major recommendation). | | See page 32 |
|  |  | | State implications for future research. | |  |
| **ACKNOWLEDGEMENTS** | |  | | | |
| Acknowledgements |  | | Describe sources of funding and other support (e.g., supply of data) and the role of funders in the review. | | See page 37 |
|  |  | | Acknowledge any information about potential conflict of interest. | |  |
| **REFERENCES** | | | | | |
| References | List all the references cited in the text. | | | | See pages 34-40 |
| **APPENDICES (tables, figures, boxes, …)** | | | | | |
| Flow diagram |  | | | | |
| Table of characteristics of included studies | Summarize key characteristics of the included studies in a table such as:   - Source (first author, year), - Study design, - Number of participants and participants (e.g., age, sex, other), - Setting (e.g., types and number of organizations), - Intervention/exposure (if applicable), or relevant - considerations for non-intervention studies, - Outcomes (if applicable), or relevant considerations for - non-intervention studies, - Quality rating (if applicable), - Further comments on key characteristics of the study - (according to the review question),   Different tables may be needed: For example, tables for quantitative studies, for qualitative studies, and for mixed methods studies. | | | Appendix file D | |
| Tables and figures on results of the synthesis | Provide illustrations for results of the synthesis such as:   - Quantitative synthesis: statistical summary, descriptive table, forest plots, etc. - Qualitative synthesis: list of categories, list of themes and subthemes, concept maps, framework, etc. | | | See Tables 2-5, Figure 1 | |

*Adapted from:* Moher D, Liberati A, Tetzlaff J, Altman DG, The PRISMA Group (2009).

Preferred Reporting Items for Systematic Reviews and Meta-Analyses: The PRISMA Statement. PLoS Med 6(6): e1000097. doi:10.1371/journal.pmed1000
